# Adherence to medications, lifestyle advice, and self-monitoring for type 2 diabetes and hypertension in sub-Saharan Africa: A systematic review, meta-analysis, and interactive network analysis

**DOI:** 10.1371/journal.pmed.1005189

**Published:** 2026-08-03

**Authors:** Angeliki Apostolou, Reuben Simfukwe, Adrias Saint, Bridget Mushani, Hannah Chekhchar, Pearl Aovare, Kouamivi M. Agboyibor, Karlijn A. C. Meeks, George F. Mkoma, Charles Agyemang, Felix P. Chilunga

**Affiliations:** 1 Department of Public and Occupational Health, Amsterdam Public Health Research Institute, Amsterdam University Medical Centers, University of Amsterdam, Amsterdam, The Netherlands; 2 Clinical Medicine Program, Kamuzu University of Health Sciences, Blantyre, Malawi; 3 Clinical Research Education and Management Services (CREAMS), Queen Elizabeth Central Hospital, Blantyre, Malawi; 4 Department of Population, Family and Reproductive Health, School of Public Health, University of Ghana, Accra, Ghana; 5 Department of Noncommunicable Disease, World Health Organization (WHO) HQ, Geneva, Switzerland; 6 Division of Endocrinology, Diabetes and Nutrition, Department of Medicine, University of Maryland School of Medicine, Baltimore, Maryland, United States of America; 7 Department of Epidemiology Research, Statens Serum Institut, Copenhagen, Denmark; 8 Department of Public Health, Section of Health Services Research, University of Copenhagen, Copenhagen, Denmark; 9 Division of Endocrinology, Diabetes and Metabolism, Department of Medicine, The Johns Hopkins University School of Medicine, Baltimore, Maryland, United States of America; Wuqu’ Kawoq | Maya Health Alliance, GUATEMALA

## Abstract

**Background:**

Type 2 diabetes and hypertension are major public health challenges in sub-Saharan Africa, yet treatment control remains poor. Patient adherence to key treatment pillars (medication, lifestyle advice, and self-monitoring) is essential for treatment control, yet adherence across these pillars has not been comprehensively synthesized. We conducted a systematic review and meta-analysis to map adherence to medication, lifestyle advice, and self-monitoring for type 2 diabetes and hypertension in sub-Saharan Africa, and applied a network analysis to identify cross-cutting determinants as priority candidates for future interventional studies.

**Methods and findings:**

We searched six academic databases, and Google Scholar, for observational and interventional studies in adults with type 2 diabetes or hypertension in sub-Saharan Africa published between January 1, 2004, and May 14, 2026. Risk of bias was assessed using Newcastle–Ottawa Scale and Cochrane RoB 2. We used random-effects models to pool adherence proportions and conducted subgroup analyses and meta-regressions. Cross-cutting determinants were identified using interactive network analysis with noteworthiness scores. We included 312 studies with 108,014 participants from 28 countries. Pooled adherence was 67% (95% CI [60, 73]) for antidiabetic medications, 51% (95% CI [44, 58]) for antihypertensive medications, 44% (95% CI [38, 49]) for dietary recommendations, 42% (95% CI [37, 47]) for physical activity, 85% (95% CI [82, 88]) for alcohol abstinence, 95% (95% CI [94, 96]) for smoking cessation, 18% (95% CI [12, 27]) for glucose monitoring, and 28% (95% CI [16, 45]) for blood pressure monitoring. Overall self-care adherence was 37% (95% CI [30, 46]) for type 2 diabetes and 35% (95% CI [29, 42]) for hypertension. Heterogeneity was high (*I*^2^ > 95%, *p* < 0.001 throughout). Education, self-efficacy, and social support emerged as cross-cutting determinants most consistently associated with adherence. A key limitation is the high statistical heterogeneity, which persisted despite random-effects modeling and subgroup analyses.

**Conclusions:**

Adherence across treatment pillars is suboptimal. Education, self-efficacy, and social support represent priority candidates for future interventional studies aimed at improving adherence. The review was registered with PROSPERO (CRD42024626793).

## Author summary

**Why was this study done?:**

- Type 2 diabetes and high blood pressure are rising rapidly in sub-Saharan Africa, but most patients cannot keep their condition under control.
- Good control depends on patients sticking to three parts of treatment: taking medication, following lifestyle advice (diet, exercise, avoiding smoking and alcohol), and monitoring their condition at home.
- Evidence on adherence across all three parts had not been brought together, nor had factors been identified that could be tested in future studies to improve adherence.

**What did the researchers do and find?:**

- We reviewed 312 studies involving more than 108,000 people from 28 sub-Saharan African countries to estimate adherence to each part of treatment and identify the factors most consistently associated with it.
- Fewer than half of patients followed all parts of treatment together; adherence was lowest for diet, exercise, and home monitoring, and highest for avoiding alcohol and smoking, with better adherence among women, younger adults, urban residents, and people in East Africa.
- Education, confidence in managing one’s own health, and social support stood out as the factors most consistently associated with better adherence across all parts of treatment.

**What do these findings mean?:**

- Adherence to treatment for diabetes and high blood pressure in sub-Saharan Africa is suboptimal and has not improved over the past two decades, signaling an urgent need for action from policymakers and health systems.
- Education, patient confidence, and social support are the most promising candidates for future interventional studies aiming to improve adherence across all parts of treatment.
- A main limitation is that most included studies were observational and relied on patients reporting their own adherence, so the identified factors should be seen as priorities for future testing rather than proven causes.

## Abstract 3

In a systematic review and meta-analysis, Angeliki Aposolou, Reuben Simfukwe and colleagues assessed adherence to different aspects of type 2 diabetes and hypertension treatment in sub-Saharan Africa.

## Introduction

Cardiometabolic diseases such as type 2 diabetes and hypertension are a major public health challenge in sub-Saharan Africa [1–3]. While infectious diseases were the predominant health burden before the 1990s [4], the prevalence of cardiometabolic diseases has risen sharply over the past three decades, contributing substantially to morbidity and mortality [1–3]. For example, the prevalence of type 2 diabetes among adults has nearly doubled, increasing from 6.4% in 1990 to 10.5% in 2021 [1], while hypertension now affects about 37.4% of adults [5]. This rapid rise has been attributed to a complex interplay of factors, including rapid urbanization, lifestyle transitions, adverse early-life exposures, population aging, and limited access to preventive and curative health services [2,6].

Although interventions have been introduced at the global level (e.g., World Health Organisation HEARTS technical Packages for hypertension and diabetes, Global noncommunicable disease (NCD)s best buys) [7,8], the regional level (e.g., African Union’s strategic frameworks on NCDs) [9], the national level (e.g., integration of diabetes and hypertension care into primary healthcare programs) [10], and the community level (e.g., task-shifting to nurses and community health workers) [11], the control of type 2 diabetes and hypertension in clinical practice remains a major challenge. For instance, a meta-analysis of 74 studies in sub-Saharan Africa showed that only 30% of patients with type 2 diabetes achieve adequate glucose control [12], and nearly half of those treated for hypertension remain uncontrolled [13]. Complications are also common, with one in three patients (~35%) affected by diabetic eye disease, chronic kidney disease, stroke, or heart failure [14]. This contrasts sharply with high-income countries, where more than 75% of patients achieve good type 2 diabetes and hypertension control [15,16], and complication rates are below 10% [15].

One major factor for achieving control of type 2 diabetes and hypertension is patient adherence to the treatment [17–19]. The treatment for cardiometabolic diseases is long-term and often lifelong and rests on three key pillars: (i) pharmacological therapy (glucose-, blood pressure-, and lipid-lowering medication, often in combination), (ii) lifestyle modification (dietary change, physical activity, weight management, and reduction of harmful behaviors such as smoking and excessive alcohol use), and (iii) self-monitoring and home-based care (tracking blood glucose, blood pressure, and weight, and adjusting behaviors or treatment when necessary) [20,21]. Patients are therefore required to adhere to all three pillars to achieve better disease control and prevent complications [20,21].

While adherence to all cardiometabolic disease treatment pillars is central to cardiometabolic disease control, especially in sub-Saharan Africa where prevalence is rising and control remains poor [1,2,12], region-wide data remains scarce. To date, pooled estimates exist only for antihypertensive medication adherence (~44%) [22]. However, there is no pooled data on adherence to type 2 diabetes medications, lifestyle advice, or self-monitoring practices, nor on adherence to the overall treatment strategy (comprehensive self-care) in the region. Similarly, little is known about the cross-cutting determinants that shape adherence across all treatment pillars and that could serve as central targets for intervention studies. This gap also extends to relevant subgroups defined by rural versus urban residence, sex, age, and geographic regions (East, West, Central, and Southern Africa).

To fill this large gap and provide actionable evidence for health professionals and policymakers, we conducted a comprehensive mapping of patient adherence across medication, lifestyle advice, and self-monitoring for type 2 diabetes and hypertension in sub-Saharan Africa. We also aimed to provide pooled evidence on adherence to all treatment pillars (i.e., overall self-care, reported by studies using composite multi-domain adherence tools). Additionally, we used an interactive network analysis to identify cross-cutting determinants most consistently associated with adherence across all three treatment pillars, which represent priority candidates for future interventional studies aimed at improving adherence.

## Methodology

### Protocol and registration

This systematic review and meta-analysis was conducted in accordance with the Preferred Reporting Items for Systematic Reviews and Meta-Analyses (PRISMA) 2020 guidelines, and is reported in accordance with the PRISMA guideline (S1 Checklist). The study protocol was prospectively registered in the International Prospective Register of Systematic Reviews (PROSPERO; registration number CRD42024626793).

### Eligibility criteria

We included original quantitative studies conducted in sub-Saharan Africa that assessed adherence to at least one of the three main treatment pillars of cardiometabolic disease in adults (≥18 years) diagnosed with type 2 diabetes, hypertension, or both. The three treatment pillars were defined as: (i) medication adherence, referring to the use of prescribed antihypertensive or antidiabetic medication; (ii) lifestyle adherence, including dietary modification, adequate physical activity, non-smoking, and avoidance of harmful alcohol consumption; and (iii) self-monitoring adherence, referring to home-based monitoring of blood pressure and/or blood glucose. Eligible study designs were observational (cross-sectional, cohort, or case–control), interventional, and mixed-methods studies (the quantitative part only). We excluded systematic reviews, meta-analyses, case reports, qualitative studies, and studies without relevant adherence outcomes. We restricted the search to publications in English between 1 January 2004 and 14 May 2026 to capture both earlier trends in adherence patterns during the rapid rise of cardiometabolic diseases in Africa (2004–2014) and more recent, policy-relevant evidence from the past decade (2015–2026 (May)).

### Information sources and search strategy

We systematically searched six electronic databases: PubMed, Embase, Cochrane Library, Web of Science, African Journals Online (AJOL), and CINAHL. To complement the peer-reviewed evidence, we searched Google Scholar to capture studies not yet indexed in major databases, as literature suggests that only about one-quarter of sub-Saharan African biomedical journals are indexed in international databases [23,24]. In addition, the reference lists of all included studies and relevant systematic reviews were hand-searched to identify further eligible studies. The search strategy combined controlled vocabulary terms (MeSH and Emtree) with free-text keywords for type 2 diabetes, hypertension, adherence, compliance, and sub-Saharan Africa. To ensure comprehensive coverage, the names of all countries in the region were included. Full electronic search strategies for each database are provided in S1 Appendix.

### Study selection and data extraction

All retrieved records were imported into Covidence (Veritas Health Innovation, Melbourne, Australia) for automatic deduplication and screening. A pilot screening of the first 20 articles was conducted by the search team to standardize the extraction process (RS, AS, AA, BM and FPC). Title and abstract screening was performed independently in reviewer pairs (RS and AS; AA and BM), followed by full-text review of potentially eligible articles. Disagreements at either stage were resolved through discussion within the reviewer pairs, and when consensus could not be reached, they were resolved in consultation with the senior reviewer (FPC). Reasons for exclusion at the full-text stage were recorded in detail.

Data extraction was conducted in Covidence using a standardized and piloted form. Two reviewers from the assigned pairs independently extracted data from each study. Extracted variables included study characteristics (author, year, country, design, setting, and sample size), participant characteristics (mean or median age and sex distribution), adherence measurement methods (e.g., Morisky Medication Adherence Scale, Hill-Bone Compliance Scale, Summary of Diabetes Self-Care Activities scale), prevalence of adherence within each treatment pillar (medication, lifestyle, self-monitoring, and overall self-care [i.e., adherence to all three pillars, extracted from studies that directly measured adherence across all three pillars combined using composite multi-domain adherence assessment tools]), and determinants of adherence (including reported effect estimates such as odds ratios, beta coefficients, hazard ratios, and correlation coefficients). Disagreements in data extraction were resolved through discussion within reviewer pairs, with unresolved issues referred to the senior reviewer (FPC). For longitudinal cohort studies and randomized controlled trials, adherence prevalence was extracted from baseline measurements to ensure comparability with cross-sectional studies, whereas effect estimates for determinants of adherence were taken from follow-up, as this is when the effect of the determinant or intervention is captured by design and best reflects the temporal or post-intervention association.

### Meta-analysis of adherence proportions

For the included studies, we conducted meta-analyses of adherence proportions using random-effects logistic-normal generalized linear mixed models (GLMM) in R (version 4.3.2) with the *meta* and *metafor* packages. Proportions were pooled on the logit scale and back-transformed for interpretation. Between-study heterogeneity was quantified using the *I*^2^ statistic, with 95% prediction intervals reported where appropriate.

Subgroup analyses were conducted by disease type (type 2 diabetes versus hypertension), treatment pillar (medication, lifestyle, self-monitoring, overall self-care), participant age (≥50 years versus < 50 years; cutoff informed by major aging studies in sub-Saharan Africa such as WHO-SAGE and INDEPTH, where the cutoff may take into account the region’s lower life expectancy than in high-income settings) [25,26], proportion of women in the sample (≥50% versus < 50%; distinguishing predominantly male versus female study populations), study setting (urban, rural, or mixed), geographic subregion (West, East, Central, Southern Africa), and study period (2004–2014 versus 2015–2026). To complement these descriptive comparisons, meta-regression was performed using the same variables as study-level covariates. Age (continuous, per 10 years), proportion of women (continuous, per 10%), and study period (continuous, per 10 years) were modeled to retain statistical power, while the remaining variables were retained as categorical as they are naturally categorical. Pooled estimates with 95% confidence intervals (CIs) were presented in forest plots.

### Bias assessment for included studies

The methodological quality of included studies was assessed by design. Cohort studies were evaluated using the Newcastle–Ottawa Scale (NOS, maximum 9 stars), cross-sectional studies with the NOS adapted for cross-sectional designs (maximum 10 stars) and randomized controlled trials with the Cochrane Risk of Bias 2 (RoB 2) tool. For the NOS, studies were classified as low risk of bias (7–9 stars for cohort NOS; 7–10 stars for adapted NOS), moderate risk (5–6 stars for both), or high risk (0–4 stars for both). RoB 2 assessments were summarized as low risk, some concerns, or high risk of bias. Two reviewer teams (RS and AS; AA and BM) independently performed all assessments, with disagreements resolved by consensus or the senior reviewer (FPC). Publication bias was assessed in R (version 4.3.2) using the *metafor* package. Funnel plots were visually inspected for asymmetry, and Egger’s regression test was applied to statistically evaluate small-study effects.

### Sensitivity analyses

Sensitivity analyses were conducted to test the robustness of the pooled adherence estimates by excluding studies at high risk of bias and by restricting analyses to those that used standardized, validated adherence tools. Commonly used instruments included the Morisky Medication Adherence Scale (MMAS-8), the Hill-Bone Compliance Scale, and the Summary of Diabetes Self-Care Activities (SDSCA). Details of the instruments used in each study, and whether a standardized tool was applied, are provided in S2 Appendix.

### Interactive network analysis

As a large number of determinants have been reported across studies, we conducted an interactive network analysis in R (version 4.3.2) to map and prioritize those that appear most consistently across studies in the same direction, providing a structured overview of potential targets to inform the design of future interventional studies aimed at improving adherence. The full workflow, including data processing and visualization code, is available on GitHub (https://github.com/hannahchek/networkanalysis). Determinants and their effect estimates (odds ratios [ORs], relative risks [RRs], prevalence ratios [PRs], hazard ratios [HRs], *β* coefficients, or correlation coefficients) were extracted together with their linked outcomes, grouped into the three treatment pillars (medication, lifestyle, self-monitoring) [27,28]. For HRs, we first approximated RRs using the VanderWeele method incorporating the baseline event rate, and then converted these RRs to ORs using the Zhang and Yu formula [27,29]. All effect measures were converted into ORs using established methods (e.g., Zhang and Yu for RRs, Chinn for correlations). For determinants reported as categorical variables with multiple ordered levels (e.g., education with categories of none, primary, secondary, tertiary), we extracted the OR comparing the highest versus the lowest reported category (e.g., tertiary versus no education) to capture the maximum contrast and ensure consistency across studies. A harmonized dataset was then created that retained for each determinant the OR, comparison, direction of effect, and associated outcome.

To guide interpretation, we developed a composite Noteworthiness Score that ranked determinant–outcome associations by combining two components: the absolute magnitude of the effect size and the number of studies supporting the association (https://github.com/hannahchek/networkanalysis). Each component was converted into percentile ranks and then blended, with a weighting of 60% for effect size and 40% for study count (Noteworthiness = 0.6 × rank(|log(OR)|) + 0.4 × rank(*n*)). This balance was chosen after testing multiple alternatives (e.g., 50/50, 70/30, and 80/20): equal weighting tended to reward frequently reported but weak effects, while higher weighting for effect size overemphasized large but less replicated associations. The 60/40 scheme minimized both risks, produced stable rankings under sensitivity checks, and ensured that associations were prioritized when they were both strong and consistently replicated. Scores ranged from 0.06 to 0.85. To interpret the rankings, we inspected the distribution of Noteworthiness Scores and identified a tight upper-tail cluster of associations (scores 0.775–0.850) separated from the descending tail by the largest gap in the upper distribution (rank 5–6, Δ = 0.025). This cluster fell within the top 5% of the distribution, and scores in this region were therefore interpreted as the most influential within the network.

### Use of artificial intelligence tools

During the preparation of this work, the authors used Claude (Anthropic) for language editing. It was not used to generate data, perform analyses, or draw conclusions. All output was reviewed and verified by the authors, who take full responsibility for the final manuscript.

## Results

### Study selection

**Fig 1:**
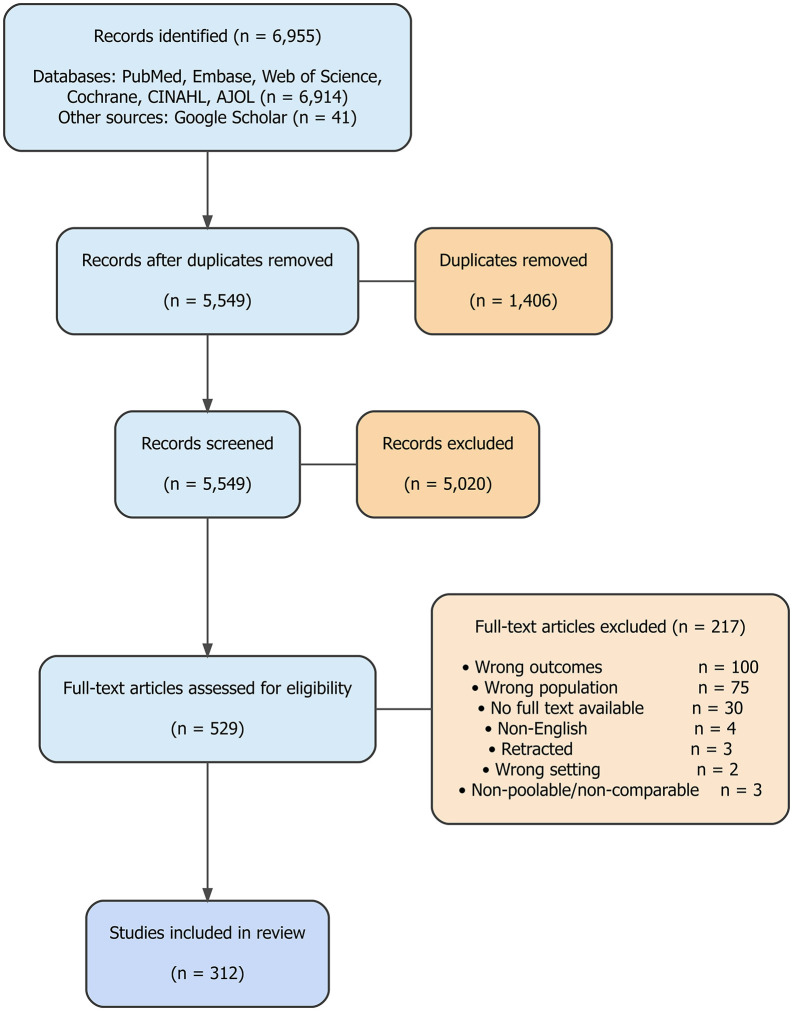
PRISMA 2020 flow diagram of study selection for the systematic review and meta-analysis of adherence to medication, lifestyle advice, and self-monitoring for type 2 diabetes and hypertension in sub-Saharan Africa. The flow diagram illustrates the identification, screening, eligibility assessment, and inclusion of studies. The number of records identified from each information source is shown, including six academic databases (PubMed, Embase, Web of Science, Cochrane Library, CINAHL [Cumulative Index to Nursing and Allied Health Literature], and AJOL [African Journals Online]) and a Google Scholar search engine query, followed by duplicates removed before screening, records excluded at title and abstract screening, full-text articles assessed for eligibility, full-text exclusions with reasons, and the final number of studies included in the review. n, number of records or studies; PRISMA, Preferred Reporting Items for Systematic Reviews and Meta-Analyses.

The search identified 6,955 records (6,914 from academic databases and 41 from Google Scholar). After removing 1,406 duplicates, 5,549 records were screened, and 5,020 were excluded at title/abstract stage. Of 529 full texts assessed, 217 were excluded in total. Reasons were; wrong outcomes (100), wrong population (75), no full text (30), non-English (4), retracted (3), wrong setting (2) and non-comparable (3). A total of 312 studies met eligibility criteria and were included in the review (Fig 1).

### Study characteristics

The 312 included studies comprised 108,014 participants across 28 sub-Saharan African countries, with Ethiopia (43.9%) and Nigeria (19.6%) contributing the largest share, followed by Ghana (8.3%) and South Africa (8.3%; Table 1; S2 Appendix). Most studies were published in or after 2014 (91.3%) and were predominantly cross-sectional (88.5%), with fewer cohort (3.5%), randomized controlled trial (2.2%), case–control (1.3%), quasi-experimental (2.9%), and mixed-methods (1.6%, quantitative part only extracted) designs. Nearly half were conducted in urban settings (46.5%), 44.6% in mixed urban–rural populations, and 9.0% in rural settings. Sample sizes ranged from 24 to 2,870, the pooled mean participant age was 55.6 years (with medians treated as means where necessary), and the mean female proportion across studies was 57.2%.

**Table 1:** Characteristics of the studies included in the analysis.

Characteristic	Description	*n* (%)/Values
Total participants	All included studies (312 studies)	108,014
Publication period	Studies published before 2014	27 (8.7%)
	Studies published in or after 2014	285 (91.3%)
Countries (28 in total)	Ethiopia	137 (43.9%)
	Nigeria	61 (19.6%)
	South Africa	26 (8.3%)
	Ghana	25 (8.0%)
	Other countries + multi-country studies	63 (20.2%)
Study design	Cross-sectional	276 (88.5%)
	Cohort study (prospective + retrospective)	11 (3.5%)
	Case–control study	4 (1.3%)
	Randomized controlled trial (RCT)	7 (2.2%)
	Non-randomized experimental study (quasi-experimental, pre–post, etc.)	9 (2.9%)
	Mixed-methods study (quantitative part only extracted)	5 (1.6%)
Study setting	Mixed urban–rural	139 (44.6%)
	Urban	145 (46.5%)
	Rural	28 (9.0%)
Sample size per study	Range across individual studies	24–2,870 participants
Pooled mean age	Reported in 246 studies	55.6 years
Pooled mean women %	Reported in 304 studies	57.2%

Summary of study characteristics from 312 included studies across 28 sub-Saharan African countries. Values are presented as number (percentage) unless otherwise stated. Pooled mean age and pooled mean proportion of women are calculated only from studies reporting these numbers. Unique countries represented are Benin, Botswana, Burkina Faso, Cameroon, Côte d’Ivoire, Democratic Republic of the Congo, Republic of the Congo (Congo-Brazzaville), Eritrea, Ethiopia, Gabon, The Gambia, Ghana, Guinea, Kenya, Lesotho, Malawi, Mauritania, Mozambique, Namibia, Nigeria, Rwanda, Senegal, Somalia, South Africa, Tanzania, Togo, Uganda, and Zimbabwe.

### Pooled adherence across treatment pillars and overall

**Fig 2:**
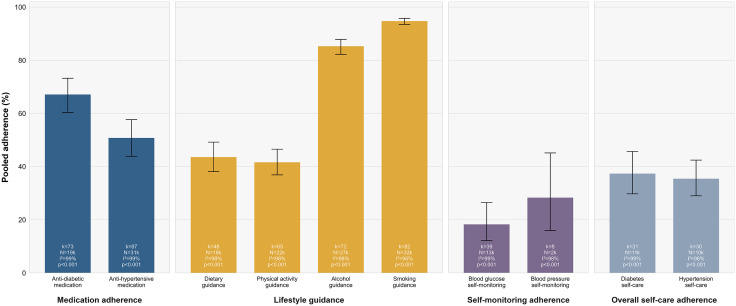
Pooled adherence proportions across treatment pillars for type 2 diabetes and hypertension in sub-Saharan Africa. Bar chart showing pooled adherence proportions (%) with 95% confidence intervals (error bars) across the three treatment pillars and for overall self-care adherence. The medication adherence panel shows anti-diabetic and anti-hypertensive medication adherence; the lifestyle guidance panel shows adherence to dietary recommendations, physical activity, alcohol abstinence, and smoking cessation; the self-monitoring panel shows adherence to blood glucose and blood pressure self-monitoring; and the overall self-care panel shows pooled adherence across all three pillars combined, separately for diabetes and hypertension. Pooled estimates were derived from random-effects logistic-normal generalized linear mixed models (GLMM) using the logit transformation. *I*^2^, heterogeneity statistic representing the percentage of variation attributable to between-study heterogeneity; *k*, number of studies contributing to the pooled estimate; *N*, total number of participants contributing to the pooled estimate; p, p-value for heterogeneity from Cochran’s *Q* test.

Pooled adherence was 67% (95% CI [60, 73], *I*^2^ = 99%, *p* < 0.001) for anti-diabetic medication and 51% (95% CI [44, 58], *I*^2^ = 99%, *p* < 0.001) for antihypertensive medication. Lifestyle adherence was lower overall, with 44% (95% CI [38, 49], *I*^2^ = 98%, *p* < 0.001) for dietary recommendations and 42% (95% CI [37, 47], *I*^2^ = 98%, *p* < 0.001) for physical activity, though adherence was higher for alcohol use recommendations at 85% (95% CI [82, 88], *I*^2^ = 98%, *p* < 0.001) and for tobacco abstinence at 95% (95% CI [94, 96], *I*^2^ = 96%, *p* < 0.001). Self-monitoring adherence was poorest, at 18% (95% CI [12, 27], *I*^2^ = 99%, *p* < 0.001) for blood glucose and 28% (95% CI [16, 45], *I*^2^ = 98%, *p* < 0.001) for blood pressure. Overall self-care adherence, as directly assessed in studies using composite multi-domain adherence tools, was 37% (95% CI [30, 46], *I*^2^ = 99%, *p* < 0.001) among patients with type 2 diabetes and 35% (95% CI [29, 42], *I*^2^ = 98%, *p* < 0.001) among those with hypertension (Fig 2, S3 Appendix).

### Subgroup analyses and meta-regressions by study characteristics

We report subgroup analyses summaries in Appendices S4 to S7 and the forest plots in Appendix S8. Anti-diabetic adherence was similar in younger and older populations, but was higher in female-majority than in male-majority study populations, in urban than in rural settings, and in Central/Southern and East Africa (vs. West Africa). Antihypertensive adherence followed a broadly similar pattern, being higher in female-majority than in male-majority study populations and in urban than in rural settings, and additionally higher in younger than in older populations and in East Africa (vs. West and Central/Southern Africa). For both medication classes, adherence was similar in more recent studies (2015–2026) and earlier studies (2004–2014, Appendix S4 and S8). Meta-regression with age (continuous, per 10 years), proportion of women (continuous, per 10%), and study period (continuous, per 10 years) showed the same directional patterns but did not confirm them as statistically significant (Table 2).

**Table 2:** Meta–regressions of pooled adherence by study characteristics.

Adherence categories	Study characteristics	*β* coefficient	95% CI	*P*-value
**Medications (pooled adherence)**				
**Anti–diabetic medications**				
	Mean age of participants (per 10 years)	−0.53	[−1.10, 0.05]	0.074
	Mean percentage of females (per 10%)	−0.07	[−0.34, 0.20]	0.609
	Study year (per 10-year increase)	0.30	[−0.44, 1.03]	0.428
	Region: Southern/Central Africa vs. East Africa	0.30	[−0.63, 1.24]	0.524
	Region: West Africa vs. East Africa	−0.63	[−1.28, 0.03]	0.060
	Urban vs. rural	0.38	[−1.06, 1.83]	0.604
**Anti–hypertensive medications**				
	Mean age of participants (per 10 years)	−0.18	[−0.74, 0.37]	0.521
	Mean percentage of females (per 10%)	0.12	[−0.13, 0.36]	0.349
	Study year (per 10-year increase)	0.02	[−0.62, 0.66]	0.950
	Region: Southern/Central Africa vs. East Africa	−0.33	[−1.06, 0.41]	0.388
	Region: West Africa vs. East Africa	−0.57	[−1.19, 0.04]	0.066
	Urban vs. rural	0.20	[−0.88, 1.27]	0.720
Lifestyle recommendations (pooled adherence)				
**Dietary recommendations**				
	Mean age of participants (per 10 years)	0.11	[−0.45, 0.67]	0.707
	Mean percentage of females (per 10%)	−0.01	[−0.28, 0.26]	0.946
	Study year (per 10-year increase)	−0.38	[−1.21, 0.44]	0.362
	Region: Southern/Central Africa vs. East Africa	−0.08	[−1.21, 1.05]	0.890
	Region: West Africa vs. East Africa	−0.34	[−1.08, 0.40]	0.371
	Urban vs. rural	0.50	[−0.70, 1.70]	0.413
**Physical activity recommendations**				
	Mean age of participants (per 10 years)	0.04	[−0.32, 0.39]	0.843
	Mean percentage of females (per 10%)	0.03	[−0.18, 0.23]	0.785
	Study year (per 10-year increase)	−0.56	[−1.27, 0.16]	0.129
	Region: Southern/Central Africa vs. East Africa	−0.47	[−1.10, 0.16]	0.140
	Region: West Africa vs. East Africa	0.44	[−0.24, 1.11]	0.208
	Urban vs. rural	−0.13	[−1.12, 0.87]	0.802
**Alcohol use recommendations**				
	Mean age of participants (per 10 years)	0.56	[0.17, 0.95]	0.005
	Mean percentage of females (per 10%)	0.29	[0.05, 0.53]	0.019
	Study year (per 10-year increase)	0.27	[−0.41, 0.95]	0.430
	Region: Southern/Central Africa vs. East Africa	0.38	[−0.43, 1.19]	0.360
	Region: West Africa vs. East Africa	0.30	[−0.32, 0.92]	0.346
	Urban vs. rural	−0.85	[−1.84, 0.15]	0.095
**Tobacco smoking recommendations**				
	Mean age of participants (per 10 years)	0.72	[0.29, 1.16]	0.001
	Mean percentage of females (per 10%)	0.32	[0.09, 0.54]	0.005
	Study year (per 10-year increase)	−0.31	[−0.93, 0.31]	0.325
	Region: Southern/Central Africa vs. East Africa	−0.78	[−1.52, -0.04]	0.038
	Region: West Africa vs. East Africa	0.40	[−0.18, 0.97]	0.177
	Urban vs. rural	0.77	[−0.12, 1.66]	0.090
[Self, monitoring (pooled adherence)]				
**Self–monitoring of blood glucose**				
	Mean age of participants (per 10 years)	0.01	[−1.13, 1.16]	0.985
	Mean percentage of females (per 10%)	−0.66	[−1.15, -0.17]	0.009
	Study year (per 10-year increase)	1.32	[0.12, 2.52]	0.031
	Region: Southern/Central Africa vs. East Africa	−2.19	[−3.71, -0.66]	0.005
	Region: West Africa vs. East Africa	−0.49	[−1.72, 0.74]	0.432
	Urban vs. rural	NA	NA	NA
**Self–monitoring of blood pressure**				
	Mean age of participants (per 10 years)	0.58	[−0.72, 1.89]	0.382
	Mean percentage of females (per 10%)	0.13	[−0.47, 0.73]	0.676
	Study year (per 10-year increase)	−1.15	[−5.50, 3.20]	0.603
	Region: Southern/Central Africa vs. East Africa	NA	NA	NA
	Region: West Africa vs. East Africa	1.70	[0.76, 2.64]	<0.001
	Urban vs. rural	NA	NA	NA
[Overall self, care adherence (pooled adherence, all three treatment pillars)]				
**Overall self–care for diabetes (all three pillars)**				
	Mean age of participants (per 10 years)	0.31	[−0.53, 1.15]	0.470
	Mean percentage of females (per 10%)	−0.48	[−0.82, -0.14]	0.006
	Study year (per 10-year increase)	−0.15	[−1.22, 0.92]	0.785
	Region: Southern/Central Africa vs. East Africa	−1.48	[−2.27, -0.69]	<0.001
	Region: West Africa vs. East Africa	NA	NA	NA
	Urban vs. rural	0.05	[−1.06, 1.17]	0.923
**Overall self–care for hypertension (all three pillars)**				
	Mean age of participants (per 10 years)	−0.31	[−0.97, 0.36]	0.368
	Mean percentage of females (per 10%)	−0.28	[−0.62, 0.06]	0.105
	Study year (per 10-year increase)	0.13	[−0.97, 1.23]	0.814
	Region: Southern/Central Africa vs. East Africa	−1.06	[−2.21, 0.09]	0.070
	Region: West Africa vs. East Africa	0.07	[−0.87, 1.02]	0.884
	Urban vs. rural	−0.06	[−1.15, 1.04]	0.918

Meta-regression models were fitted as univariable random-effects meta-regressions using logistic-normal generalized linear mixed models (GLMM), with each study characteristic entered as a fixed-effect moderator on the logit scale; where a GLMM did not converge, the estimate was obtained from a logit-scale inverse-variance meta-regression with the Hartung-Knapp adjustment. *β* coefficients and 95% confidence intervals (CI) are reported on the logit scale. Continuous covariates were rescaled per 10 units (mean age per 10 years, percentage of females per 10 percentage points, study year per 10-year increase). Reference categories were East Africa for region and rural for residence; mixed-residence studies were excluded from the urban vs. rural contrast. Results marked in bold are statistically significant (*p* < 0.05, from the Wald test for each meta-regression moderator). NA indicates insufficient studies for comparison in one or more groups. Regions were defined according to the UN geoscheme; Southern and Central Africa were merged owing to few studies and shared contexts (e.g., SADC membership), while East and West Africa were analyzed separately given sufficient studies and distinct epidemiological settings.

For lifestyle, adherence to diet and physical activity recommendations was low across all groups, with dietary adherence somewhat lower in rural than in urban populations, while adherence to alcohol and tobacco recommendations was consistently high, particularly in older adults, in female-majority study populations, and, for alcohol, in rural populations (Appendix S5 and S8). Meta-regression confirmed that higher mean participant age and a higher proportion of female participants were positively associated with adherence to both alcohol and tobacco recommendations, and that adherence to tobacco recommendations was lower in Southern/Central Africa than in East Africa (Table 2).

For self-monitoring, adherence was poor overall (S6 and S8 Appendices). Blood glucose monitoring was lower in female-majority study populations and in Central/Southern Africa, while blood pressure monitoring was higher in older, female-majority, and urban populations. Meta-regression showed that adherence to blood glucose monitoring increased with later study period but was lower with a higher proportion of female participants and lower in Southern/Central Africa than in East Africa, while adherence to blood pressure monitoring was higher in West Africa than in East Africa (Table 2).

For overall self-care, adherence among people with type 2 diabetes varied substantially across subgroups, being higher in older adults, in male-majority study populations, in East Africa (vs. Central/Southern Africa), and in more recent studies (S7 and S8 Appendices). In contrast, adherence among people with hypertension was lower in older adults, in female-majority study populations, and in Central/Southern Africa (vs. East and West Africa). Meta-regression showed that overall self-care adherence in type 2 diabetes was lower with a higher proportion of female participants and lower in Southern/Central Africa than in East Africa, whereas no study characteristic was significantly associated with overall self-care adherence in hypertension (Table 2).

### Sensitivity analyses

Sensitivity analyses showed that the main findings were robust. Excluding studies at high risk of bias did not materially alter the pooled estimates (S9 and S10 Appendices). Medication adherence was 64% (95% CI [55, 73], *I*^2^ = 99%, *p* < 0.001) for anti-diabetic medication and 49% (95% CI [40, 58], *I*^2^ = 99%, *p* < 0.001) for antihypertensive medication. Lifestyle adherence was 47% (95% CI [41, 53], *I*^2^ = 98%, *p* < 0.001) for diet recommendations and 42% (95% CI [36, 47], *I*^2^ = 98%, *p* < 0.001) for physical activity, while remaining high at 85% (95% CI [81, 89], *I*^2^ = 98%, *p* < 0.001) for alcohol and 94% (95% CI [93, 96], *I*^2^ = 96%, *p* < 0.001) for tobacco recommendations. Self-monitoring adherence was 19% (95% CI [11, 29], *I*^2^ = 99%, *p* < 0.001) for blood glucose and 20% (95% CI [12, 32], *I*^2^ = 97%, *p* < 0.001) for blood pressure. Overall self-care adherence was 40% (95% CI [32, 48], *I*^2^ = 98%, *p* < 0.001) for type 2 diabetes and 35% (95% CI [28, 41], *I*^2^ = 97%, *p* < 0.001) for hypertension.

Restricting analyses to studies using standardized adherence tools (i.e., excluding yes/no adherence assessments) yielded comparable results (S11 and S12 Appendices). Medication adherence was 60% (95% CI [52, 67], *I*^2^ = 99%, *p* < 0.001) for anti-diabetic medication and 46% (95% CI [37, 56], *I*^2^ = 99%, *p* < 0.001) for antihypertensive medication. Lifestyle adherence was 44% (95% CI [34, 54], *I*^2^ = 99%, *p* < 0.001) for diet recommendations and 42% (95% CI [36, 49], *I*^2^ = 98%, *p* < 0.001) for physical activity, while remaining high at 86% (95% CI [76, 92], *I*^2^ = 99%, *p* < 0.001) for alcohol recommendations (no studies using validated tools reported on tobacco use). Self-monitoring adherence was 26% (95% CI [16, 40], *I*^2^ = 98%, *p* < 0.001) for blood glucose (no studies using validated tools reported on blood pressure self-monitoring). Overall self-care adherence was 36% (95% CI [28, 46], *I*^2^ = 99%, *p* < 0.001) for type 2 diabetes and 36% (95% CI [29, 43], *I*^2^ = 98%, *p* < 0.001) for hypertension.

### Interactive network analysis

**Fig 3:**
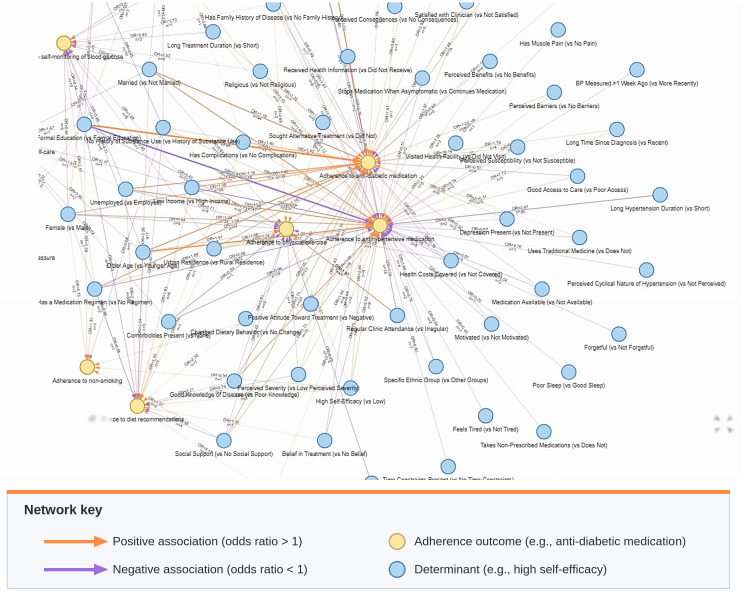
Snapshot of the interactive network graph mapping determinants of adherence across treatment pillars for type 2 diabetes and hypertension in sub-Saharan Africa. Two-dimensional snapshot of the interactive network graph showing the relationships between determinants of adherence and adherence outcomes across the three treatment pillars (medication, lifestyle, self-monitoring) for type 2 diabetes and hypertension. Yellow nodes represent adherence outcomes (e.g., adherence to anti-hypertensive medication, anti-diabetic medication, diet recommendations, blood glucose self-monitoring, non-alcohol consumption, non-smoking, and overall self-care). Blue nodes represent determinants of adherence, expressed as categorical contrasts (e.g., “No Formal Education vs. Formal Education”). Edges connecting nodes show the direction of association between each determinant and outcome: purple edges represent positive associations and orange edges represent negative associations, with edge labels showing the corresponding odds ratio. The full interactive three-dimensional version of the network, including all 169 determinant–outcome associations and Noteworthiness Scores, is available at https://hannahchek.github.io/networkanalysis/. BP, blood pressure; OR, odds ratio; vs, versus.

Given the large number of determinants reported across the included studies, we used the interactive network analysis to identify those that appear most consistently across studies in the same direction, pointing to priority candidates for future interventional studies. A total of 169 determinant–outcome associations were incorporated into the analysis. Cross-cutting determinants most consistently associated with adherence across multiple treatment pillars were identified within the top 5th percentile of all Noteworthiness Scores (NS; approximately 0.70), based on the score distribution and clustering observed in the rank plot. Lack of formal education consistently reduced adherence to diet recommendations, physical activity, and blood glucose monitoring (NS = 0.82–0.83). Positive determinants included good disease knowledge (NS = 0.79), high self-efficacy (NS = 0.78), and a positive attitude toward treatment (NS = 0.83). Being married improved dietary recommendation adherence (NS = 0.81), while membership in a diabetic association promoted anti-diabetic medication adherence (NS = 0.74). Negative experiences, such as side effects, forgetfulness, and stopping medication when asymptomatic, also scored above 0.70, indicating substantial adverse effects. An interactive 3D version of the network graph can be accessed on our online GitHub platform (https://hannahchek.github.io/networkanalysis/). A sample of the network map is provided in Fig 3.

### Bias and heterogeneity

Observational studies were generally of high quality, with 95% rated as low or moderate risk, providing a robust real-world evidence base (S13 and S14 Appendices). In contrast, the eight randomized controlled trials were weaker, with four rated high risk, three raising some concerns, and only one rated low risk, underscoring the comparatively limited strength of experimental evidence (S13 and S14 Appendices).

Publication bias was assessed using funnel plots and Egger’s regression test (S15 and S16 Appendices). Several outcomes showed no significant small-study effects, including anti-hypertensive medication (*p* = 0.273), dietary guidance (*p* = 0.070), physical activity guidance (*p* = 0.070), blood pressure self-monitoring (*p* = 0.818), and overall hypertension self-care (*p* = 0.097), suggesting minimal risk of publication bias for these domains. However, funnel-plot asymmetry was detected for anti-diabetic medication (*p* = 0.002), alcohol guidance (*p* < 0.001), smoking guidance (*p* < 0.001), blood glucose self-monitoring (*p* < 0.001), and overall diabetes self-care (*p* = 0.014), indicating possible small-study effects. Sensitivity analyses excluding high-risk-of-bias studies and restricting to standardized measurement tools confirmed that pooled estimates for the major pillars of adherence (medication, diet, and self-monitoring) remained stable, suggesting that publication bias is unlikely to have materially influenced the main conclusions.

Heterogeneity was consistently high across pooled analyses and persisted after subgroup and sensitivity analyses. This likely reflects genuine variation in adherence patterns related to differences in populations, measurement tools, and health system contexts across sub-Saharan Africa, rather than methodological flaws.

## Discussion

We provide a comprehensive mapping of adherence to treatment for type 2 diabetes and hypertension in sub-Saharan Africa across the three key treatment pillars of medications, lifestyle behaviors, and self-monitoring, and identified cross-cutting determinants as priority candidates for future interventional studies. Overall adherence to comprehensive self-care (all three pillars assessed using composite multi-domain tools) was very low. Medication adherence was slightly higher for type 2 diabetes than hypertension but suboptimal for both. Adherence to diet and physical activity was poor, while adherence to alcohol and smoking recommendations was high, and self-monitoring was the weakest pillar. Subgroup and meta-regression analyses revealed varying patterns across pillars by age, sex, residence, geography, and study period. Education, self-efficacy, and social support emerged as cross-cutting determinants most consistently associated with adherence across domains.

Type 2 diabetes and hypertension require sustained adherence across key treatment pillars: medications, lifestyle modification, and self-monitoring to achieve durable control and avert complications [20,21]. In sub-Saharan Africa, however, evidence has been fragmented, largely limited to single pillars such as antihypertensive medications [22], with little synthesis across the others or attention to shared determinants. By pooling data from more than 100,000 participants across 28 countries, this study provides a comprehensive mapping of adherence within each pillar and across all pillars together, and identifies cross-cutting determinants as priority candidates for future interventional studies.

We found that pooled adherence proportions to medications were 67% for type 2 diabetes and 51% for hypertension. These levels are suboptimal when compared with high-income countries, where adherence is generally above 70% [27,28], but mirror other low- and middle-income countries, where adherence to medication is usually around half of the studied populations [29,30]. The hypertension pooled proportions of 51% are also close to the previous meta-analysis in sub-Saharan Africa (from inception to 2023) that reported pooled proportions of 44% [22]. The higher adherence observed for type 2 diabetes compared with hypertension may be partly explained by the symptomatic nature of hyperglycemia, which reinforces treatment continuation, whereas hypertension remains largely asymptomatic and may potentially be deprioritised by patients [31,32]. Within the medication pillar, access to medication and other medication-related factors such as drug stock-outs, out-of-pocket costs, polypharmacy, side effects may play a more specific role [33–36]. For example, surveys in Ghana and Nigeria report that up to half of patients discontinue medication due to affordability [33], while facility audits in Ethiopia and Uganda show that essential cardiometabolic disease medications are unavailable up to 40% of the time [34,35].

We found that adherence to lifestyle recommendations was low for diet (44%) and physical activity (42%) recommendations but high for alcohol (85%) and smoking (95%) recommendations. This contrast may reflect differences in behavioral demand: abstaining from alcohol and tobacco often aligns with prevailing social or religious norms [37,38]. More than 80% of adults in sub-Saharan Africa report religion as central to daily life [39], with large Muslim and Christian populations placing explicit restrictions on alcohol and tobacco use [39]. By contrast, diet and physical activity require sustained behavioral change [40]. Dietary modification may require foods that are less affordable, less preferred by families, or incompatible with local cooking practices [41], while increasing physical activity often requires disrupting established routines [41]. Factors such as religion and the demands of sustained behavioral change may help explain why adherence to diet and physical activity remains lower than for alcohol and smoking [41].

We found that self-monitoring was the weakest pillar, with adherence at 18% for blood glucose and 28% for blood pressure. In high-income countries, more than 70% of patients with type 2 diabetes perform regular glucose and blood pressure monitoring at home [42,43]. The particularly low levels in sub-Saharan Africa may be partly due to the high costs of devices and strips, absence of reimbursement, limited patient education, and weak integration of self-monitoring into primary care [44,45]. Surveys in Kenya and Tanzania indicate that fewer than one in five patients with diabetes can afford glucose strips on a monthly basis, while home blood pressure monitors remain largely hospital-based [46–48]. Without such tools, patients and providers lack the feedback needed to adjust therapy in real time, which may contribute to poor disease control and preventable complications.

When all three pillars were considered together (i.e., all three pillars assessed using composite multi-domain tools), we found that comprehensive self-care was achieved by only 37% of patients with type 2 diabetes and 35% with hypertension. These figures are strikingly low, given the sharp rise in cardiometabolic disease burden and the expansion of national and regional NCD programmes [49]. In high-income countries, adherence to comprehensive self-care often exceeds 70% [50,51]. The much lower levels in sub-Saharan Africa may partly reflect health systems still oriented toward acute care [52], limited integration of chronic NCD management into primary care [53], and insufficient continuity of care and financial protection [54].

Subgroup and meta-regression analyses showed that adherence patterns varied across pillars and population groups. Antihypertensive medication adherence was higher in younger adults, women, urban residents, and East Africa; adherence to alcohol and tobacco recommendations was higher in older adults and women, and, for alcohol, in rural populations; dietary adherence was higher in urban populations; and overall hypertension self-care was higher in younger adults and men, and lower in Central/Southern Africa than in East and West Africa. These subgroup differences in adherence may reflect both health system and contextual factors. For example, women’s more frequent contact with health services, particularly through reproductive and child healthcare, can build familiarity with chronic care routines [55]. Younger adults may have greater health literacy, digital access, and fewer comorbidities, supporting adherence to diet, exercise, and monitoring recommendations [56]. Urban residents benefit from shorter travel distances, steadier drug supply, stronger diagnostic capacity, and greater availability of supportive environments for healthy living, while rural populations face long travel times, limited providers, higher costs, and more frequent stock-outs [57]. Regional variation may reflect differences in health system investment and NCD programme maturity [53,58].

While adherence is affected by a wide range of individual, social, and system-level factors, these cannot realistically be addressed one by one. Identifying determinants that appear most consistently across studies and influence multiple pillars (cross-cutting determinants) offers a more efficient strategy with potential for broader impact. In this study, education, self-efficacy, and social support emerged as cross-cutting factors most consistently associated with adherence across medications, lifestyle behaviors, and self-monitoring. This pattern points to these determinants as priority candidates for future interventional studies, which could test whether targeting them delivers multi-pillar benefits. In sub-Saharan Africa, where health literacy remains low and structured patient education is limited, such determinants may be even more decisive [59]. Future interventions studies strengthening patient knowledge, building confidence in disease self-management, and shifting treatment perceptions could therefore represent some of the promising directions levers for improving adherence in this region.

Our findings have important implications for policy and clinical practice. The rising burden of type 2 diabetes and hypertension in sub-Saharan Africa, amidst persistently poor control rates, underscores an urgent gap: patient adherence to treatment has not yet been given sufficient emphasis within regional policies or clinical guidelines [19,60]. Our study demonstrates that adherence is suboptimal across and within all three key treatment pillars (medication, lifestyle advice, self-monitoring), and that for most domains there is no clear evidence of improvement over recent decades in the region. These findings demand urgent and deliberate action from policymakers (WHO, Africa Centres for Disease Control and Prevention, national governments), guideline committees and clinical leaders to elevate adherence as a central pillar in cardiometabolic disease control strategies—because without significantly improved adherence, the potential of treatments, lifestyle interventions and monitoring cannot be realized. We identify cross-cutting determinants, namely education, self-efficacy, and social support, as priority targets for future interventional studies aimed at improving adherence across all three pillars. These should form the foundation of future adherence-enhancement strategies. Simultaneously, pillar-specific strategies (e.g., technological innovations such as digital self-monitoring tools, simplified fixed-dose medication regimens, community-based lifestyle programmes) should be evaluated to complement those foundational levers.

Moreover, our subgroup findings highlight important equity and targeting considerations: adherence was lower among older adults for antihypertensive medication and hypertension self-care, among rural populations for dietary adherence, and in Central and Southern Africa for physical activity, self-monitoring and comprehensive self-care, although these gradients were not uniform across pillars. This signals the need for future targeted policy designs and resource allocation to disadvantaged populations and geographies.

This study has both strengths and limitations. Its key strengths are its scale, a synthesis of adherence to treatment for type 2 diabetes and hypertension in sub-Saharan Africa, and its scope, encompassing all three treatment pillars and more than 100,000 participants from 28 countries. The integration of subgroup and meta-regression analyses enabled identification of groups with lower adherence in specific pillars, including older patients and rural populations for particular outcomes. The use of a 3D network analysis allowed identification of cross-cutting determinants most consistently associated with adherence across multiple pillars, providing priority candidates for future interventional studies. Limitations should be considered in interpreting the findings. First, adherence was primarily assessed through self-report, which may overestimate true levels. Second, heterogeneity across studies was substantial (*I*^2^ > 95%) and was addressed using random-effects models, which are designed for such variability, with prediction intervals reported where appropriate. Extensive efforts to identify the sources of heterogeneity, including subgroup analyses, meta-regressions, sensitivity analyses restricted to studies at low risk of bias, and sensitivity analyses restricted to studies using standardized adherence tools, did not identify a clear source, suggesting that this reflects genuine variation in adherence across populations, measurement tools, and health system contexts in sub-Saharan Africa rather than methodological flaws. The pooled estimates should therefore be interpreted as central summary measures of adherence in the region, with the subgroup and sensitivity analyses providing the context for understanding the variation around them. Third, geographic coverage was uneven, with Ethiopia and Nigeria contributing disproportionately and Central Africa and Southern Africa less represented; however, regional analyses did not show marked differences, supporting robustness of the overall conclusions. Fourth, the majority of included studies were cross-sectional, which precludes causal inference for the determinants of adherence. The cross-cutting determinants identified in the network analysis should therefore be interpreted as priority candidates for future interventional studies rather than as established causal targets. Lastly, given the reasonable number of studies contributing to each meta-regression model, non-significant findings likely reflect chance variation around a null effect, and should be interpreted with caution as they may not be reproducible in independent studies.

Adherence across all treatment pillars for type 2 diabetes and hypertension remains markedly suboptimal in sub-Saharan Africa, with fewer than half of patients achieving comprehensive self-care. Within individual pillars, adherence is lowest for diet, physical activity, and self-monitoring, while adherence to alcohol and tobacco recommendations is comparatively high. Subgroup analyses revealed higher adherence among women, younger adults, urban residents, and populations in East Africa, with no evidence of improvement over the past decade. Education, self-efficacy, and social support emerged as cross-cutting determinants most consistently associated with adherence across multiple pillars, and represent priority candidates for future interventional studies aimed at improving adherence in the region.

## Supporting information

S1 Appendix Literature search strategy. Documents the systematic search across six databases (PubMed, Embase, Cochrane Library, Web of Science, AJOL, CINAHL) plus Google Scholar gray literature, restricted to English-language publications from January 2004 to May 2026. Search terms were structured around four domains: diseases (type 2 diabetes, hypertension), adherence behaviors, determinants, and geography (sub-Saharan Africa), combined with Boolean operators. The full PubMed search string is reproduced to enable replication.
(PDF)

S2 Appendix Characteristics of included studies. Master extraction table for all 312 included studies, recording country, residence setting, year, design, duration, population, sample size, age and sex distribution, adherence outcomes with their operational definitions, and the determinants of adherence examined. Provided as a separate spreadsheet because of its size.
(XLSX)

S3 Appendix Forest plots of pooled adherence for each treatment strategy component. Random-effects meta analyses of the prevalence of adherence to anti-diabetic medications, anti-hypertensive medications, dietary guidance, physical activity guidance, alcohol guidance, smoking guidance, blood glucose self-monitoring, blood pressure self-monitoring, overall diabetes self-care, and overall hypertension self-care. Each plot shows individual study estimates with 95% confidence intervals and the pooled diamond, with *I*^2^ and *τ*^2^ summarizing between-study heterogeneity. Heterogeneity *p* values, where shown, are from Cochran’s Q test.
(PDF)

S4 Appendix Subgroup analyses of medication adherence (summary table). Pooled adherence to anti diabetic and anti-hypertensive medications stratified by mean age (under 50 versus 50 years and above), proportion of female participants (under 50% versus 50% and above), residence (urban, rural, or mixed), geographical region within sub-Saharan Africa, and publication period. Tests whether medication adherence varies systematically across patient and contextual subgroups. Differences across subgroups were tested using random-effects meta-regression (GLMM; Wald test).
(PDF)

S5 Appendix Subgroup analyses of lifestyle adherence (summary table). Pooled adherence to dietary, physical activity, alcohol, and smoking guidance stratified by the same five moderators as S4 Appendix. Identifies population and context characteristics that explain variation in lifestyle adherence. Differences across subgroups were tested using random-effects meta-regression (GLMM; Wald test).
(PDF)

S6 Appendix Subgroup analyses of self-monitoring adherence (summary table). Pooled adherence to blood glucose and blood pressure self-monitoring stratified by the same five moderators. Highlights where home self-monitoring is more or less common. Differences across subgroups were tested using random-effects meta-regression (GLMM; Wald test).
(PDF)

S7 Appendix Subgroup analyses of overall self-care adherence (summary table). Pooled composite diabetes and hypertension self-care adherence stratified by the same five moderators. Shows which subgroups demonstrate stronger overall self-care. Differences across subgroups were tested using random-effects meta-regression (GLMM; Wald test).
(PDF)

S8 Appendix Subgroup forest plots for each adherence outcome. Companion figures to Appendices 4–7. For every adherence outcome, studies are stratified by age, sex distribution, residence, geographical region, and publication period. Visualizes the subgroup pooled estimates so heterogeneity between subgroups can be inspected directly. Heterogeneity p values, where shown, are from Cochran’s Q test.
(PDF)

S9 Appendix Sensitivity analysis restricted to low risk of bias studies (summary table). Pooled adherence re estimated for each of the 10 outcomes using only studies rated low risk on the Newcastle–Ottawa Scale (7 stars or more) or Cochrane RoB 2. Tests whether the main pooled estimates are robust to exclusion of higher risk studies.
(PDF)

S10 Appendix Forest plots of the low risk of bias sensitivity analysis. Study level forest plots for each adherence outcome restricted to studies at low risk of bias. Lets readers see how pooled estimates and between-study spread change when only the most rigorous studies are pooled.
(PDF)

S11 Appendix Sensitivity analysis restricted to studies using validated adherence measurement tools (summary table). Pooled adherence re-estimated using only studies that measured adherence with a validated instrument such as MMAS 4 or MMAS 8, Hill Bone, SDSCA, H SCALE, PDAQ, IPAQ, or GPAQ. Tests whether measurement tool quality changes the conclusions.
(PDF)

S12 Appendix Forest plots of the validated tool sensitivity analysis. Study level forest plots for the adherence outcomes with sufficient validated tool studies. Blood pressure self-monitoring is omitted because too few studies used a validated instrument. Shows how pooled estimates shift when only studies with validated measurement are included.
(PDF)

S13 Appendix Study level risk of bias judgements for all included studies. Line-by-line consensus ratings for all 312 studies: Newcastle–Ottawa Scale star score and category for observational studies and Cochrane RoB 2 overall judgement for randomized controlled trials. Allows the quality rating of any individual study to be audited. Provided as a separate file.
(CSV)

S14 Appendix Risk of bias summary by adherence outcome. Cross tabulations of NOS judgements (low 7 stars or more, moderate 5–6 stars, high 4 stars or fewer) for observational studies and Cochrane RoB 2 judgements (low, some concerns, high) for randomized controlled trials, broken down by adherence outcome. Summarizes the overall methodological quality of the evidence base.
(PDF)

S15 Appendix Assessment of publication bias using Egger’s regression test (summary table). For each of the 10 adherence outcomes, reports the pooled estimate alongside Egger’s t statistic, degrees of freedom, and p value. Provides a quantitative screen for funnel plot asymmetry and small study effects.
(PDF)

S16 Appendix Funnel plots for each adherence outcome. Funnel plots paired with the Egger’s test results in S15 Appendix, one per adherence outcome. Each plot shows study adherence (logit scale) against standard error, with the pooled estimate and 95% pseudo confidence region overlaid. Asymmetry suggesting possible publication bias was observed for anti-diabetic medications, alcohol, smoking, blood glucose self-monitoring, and overall diabetes self-care. Other outcomes showed no significant asymmetry (Egger’s regression test).
(PDF)

S1 Checklist PRISMA 2020 checklist. Completed PRISMA 2020 checklist, mapping each item to its location in the manuscript. The PRISMA 2020 checklist is reproduced under the Creative Commons Attribution (CC BY 4.0) license from: Page MJ, McKenzie JE, Bossuyt PM, and colleagues. The PRISMA 2020 statement: an updated guideline for reporting systematic reviews. BMJ 2021;372:n71. https://doi.org/10.1136/bmj.n71. Available from: https://www.prisma-statement.org/.
(DOCX)

## Abbreviations

CIs: confidence intervals
GLMM: generalized linear mixed models
HRs: hazard ratios
MMAS-8: Morisky Medication Adherence Scale
NCD: noncommunicable disease
NOS: Newcastle–Ottawa Scale
ORs: odds ratios
PRs: prevalence ratios
PRISMA: Preferred Reporting Items for Systematic Reviews and Meta-Analyses
RRs: relative risks
SDSCA: Summary of Diabetes Self-Care Activities
