# Supplementary material for: Adherence to medications, lifestyle advice, and self-monitoring for type 2 diabetes and hypertension in sub-Saharan Africa: A systematic review, meta-analysis, and interactive network analysis
Source: PLoS Med. 2026 Aug 3;23(8):e1005189. doi: 10.1371/journal.pmed.1005189 (PMC13460740; doi:10.1371/journal.pmed.1005189)
Supplement: Supplementary file 1 [file pmed.1005189.s001.pdf]

Appendix 1. Literature search strategy

Information Sources

We systematically searched published and grey literature to identify studies on patient adherence to multidimensional treatment strategies for type 2 diabetes and hypertension in sub-Saharan Africa. We searched six major electronic databases: PubMed, Embase, Cochrane Library, Web of Science, African Journals Online (AJOL), and CINAHL. To capture additional sources not indexed in these databases, we also searched Google Scholar for grey literature, including theses, conference proceedings, and technical reports. The search covered the period 1 January 2004 to 14 May 2026 and included only English-language studies.

Search Strategy

We structured the search strategy around four conceptual domains. The first domain targeted diseases of interest, namely type 2 diabetes, and hypertension. The second domain captured treatment and adherence strategies, including medication adherence, lifestyle modification, self-monitoring, and self-care. In this review, we defined self-care broadly to include patients' own health management activities, such as regular home monitoring of blood pressure and blood glucose, weight tracking, diet adjustments, and other self-management practices undertaken outside the formal health system. The third domain addressed the determinants of adherence, covering both structural and individual-level factors such as barriers, facilitators, predictors, enablers, health literacy, access to care, and socioeconomic and cultural influences. The fourth domain specified the geographic scope, restricted to sub-Saharan Africa, captured using the MeSH term Africa South of the Sahara in combination with the names of all 46 countries in the region.

We combined these domains systematically using Boolean operators (AND/OR) and adapted the strategy to the indexing structure and syntax of each database to ensure both sensitivity and precision.

PubMed (MEDLINE)

In PubMed, we combined MeSH terms with free-text keywords. We paired disease terms such as *"Diabetes Mellitus, Type 2"* and "*Hypertension"* with adherence concepts including "*Medication Adherence", "Non-adherence", "Patient Compliance", "Self-Care", "Lifestyle", "Exercise", "Diet", "Health Behavior", "Self-Management", "Blood Glucose Self-Monitoring",* and "*Blood Pressure Monitoring, Ambulatory"*. We captured determinants using both controlled vocabulary ("*Risk Factors", "Health Literacy", "Health Services Accessibility", "Socioeconomic Factors", "Cultural Characteristics")* and free-text keywords such as "*barriers," "facilitators," "predictors," and "enablers."* To define the geographic setting, we applied "Africa South of the Sahara" and the names of all 46 sub-Saharan African countries. We restricted results to English-language human studies published between 2004 and 2026.

Embase

We based the Embase search on the same conceptual framework as PubMed but adapted it to Emtree terms and database-specific syntax. We included terms such as 'type 2 diabetes mellitus'/exp, 'hypertension'/exp, 'medication compliance'/exp, 'risk factor'/exp, along with their free-text equivalents. As in PubMed, we combined adherence-related terms, determinants, and the names of all 46 sub-Saharan African countries with Boolean operators. We restricted results to English-language studies published between 2004 and 2026.

Cochrane Library

We searched the CENTRAL trials register and the Cochrane Database of Systematic Reviews. We applied MeSH terms and free-text keywords spanning the four domains across all text fields to maximise sensitivity. We also screened the reference lists of relevant systematic reviews to identify additional studies. We restricted results to English-language publications from 2004 to 2026.

Web of Science

In Web of Science, we searched topic fields (title, abstract, and keywords) using disease, adherence, determinant, and regional terms. We also performed citation tracking of included studies to identify additional relevant publications. We restricted results to English-language studies published between 2005 and 2026.

African Journals Online (AJOL)

Because AJOL exclusively indexes African journals, we used simplified keyword searches such as *"diabetes adherence," "hypertension compliance," and "self-care."* We manually screened titles and abstracts for relevance. We restricted results to English-language publications from 2004 to 2026.

CINAHL

In CINAHL, we combined subject headings such as "*Diabetes Mellitus, Type 2+", "Hypertension+",* and *"Patient Compliance"* with free-text terms for adherence, determinants, and sub-Saharan Africa. We applied filters to restrict results to peer-reviewed, English-language publications within the 2004–2026 period.

Google Scholar (Grey Literature)

We searched Google Scholar to capture grey literature such as these, reports, and conference proceedings. We applied simplified keyword searches (e.g., *"adherence diabetes," "hypertension treatment adherence," "self-care cardiometabolic"*). For each query, we screened the first 200 results, consistent with best practice for systematic reviews. We included only English-language sources published between 2004 and 2026.

PUBMED BOOLEAN SEARCH STRING

(
("Diabetes Mellitus, Type 2"[MeSH Terms] OR "type 2 diabetes"[Title/Abstract] OR diabetes[Title/Abstract])
OR
("Hypertension"[MeSH Terms] OR "high blood pressure"[Title/Abstract])
)
AND
(
"Medication Adherence"[MeSH Terms] OR "Patient Compliance"[MeSH Terms] OR "Self Care"[MeSH Terms]
OR "Life Style"[MeSH Terms] OR "Diet"[MeSH Terms] OR "Exercise"[MeSH Terms] OR "Health Behavior"[MeSH Terms]
OR "Blood Glucose Self-Monitoring"[MeSH Terms] OR "Blood Pressure Monitoring, Ambulatory"[MeSH Terms]
OR "Self-Management"[MeSH Terms] OR treatment adherence[Title/Abstract] OR adherence[Title/Abstract]
OR non-adherence[Title/Abstract] OR nonadherence[Title/Abstract] OR compliance[Title/Abstract]
OR "self monitoring"[Title/Abstract] OR "self management"[Title/Abstract]
OR "lifestyle modification"[Title/Abstract] OR "home-based care"[Title/Abstract] OR "self-care"[Title/Abstract]
)
AND
(
determinants[Title/Abstract] OR predictors[Title/Abstract] OR barriers[Title/Abstract]
OR facilitators[Title/Abstract] OR enablers[Title/Abstract] OR "Risk Factors"[MeSH Terms]
OR "Health Knowledge, Attitudes, Practice"[MeSH Terms] OR "Attitude to Health"[MeSH Terms]
OR "Health Literacy"[MeSH Terms] OR "Health Services Accessibility"[MeSH Terms]
OR "Socioeconomic Factors"[MeSH Terms] OR "Cultural Characteristics"[MeSH Terms]
)
AND
(
"Africa South of the Sahara"[MeSH Terms] OR "sub-Saharan Africa"[Title/Abstract] OR SSA[Title/Abstract]
OR Angola OR Benin OR Botswana OR "Burkina Faso" OR Burundi OR Cameroon OR "Cape Verde" OR "Central African Republic"
OR Chad OR Comoros OR Congo OR "Democratic Republic of the Congo" OR Djibouti OR "Equatorial Guinea" OR Eritrea
OR Eswatini OR Ethiopia OR Gabon OR Gambia OR Ghana OR Guinea OR "Guinea-Bissau" OR "Ivory Coast" OR "Côte d'Ivoire"
OR Kenya OR Lesotho OR Liberia OR Madagascar OR Malawi OR Mali OR Mauritania OR Mauritius OR Mozambique OR Namibia
OR Niger OR Nigeria OR Rwanda OR "Sao Tome" OR "São Tomé and Príncipe" OR Senegal OR Seychelles OR "Sierra Leone"

OR Somalia OR "South Africa" OR "South Sudan" OR Tanzania OR Togo OR Uganda OR Zambia OR Zimbabwe
)
AND
("2004/01/01"[PDAT] : "2026/05/14"[PDAT])
AND English[lang]
