# Supplementary material for: Adherence to medications, lifestyle advice, and self-monitoring for type 2 diabetes and hypertension in sub-Saharan Africa: A systematic review, meta-analysis, and interactive network analysis
Source: PLoS Med. 2026 Aug 3;23(8):e1005189. doi: 10.1371/journal.pmed.1005189 (PMC13460740; doi:10.1371/journal.pmed.1005189)
Supplement: Supplementary file 2 [file pmed.1005189.s003.pdf]

Appendix 3a. Anti-diabetic medications

Random-effects meta-analysis | k = 73 studies | Total N = 19,316 | Events = 12,046

Events / N	Study	Proportion [95% CI]
82 / 469	Kamuhabwa 2014	17% [14%, 21%]
30 / 155	Bizu 2016	19% [13%, 26%]
60 / 303	Jackson 2015	20% [15%, 25%]
93 / 403	Sendekie 2022	23% [19%, 28%]
66 / 273	Mariye 2019	24% [19%, 30%]
22 / 89	Ikolaba 2023	25% [16%, 35%]
66 / 226	Adisa 2016	29% [23%, 36%]
63 / 188	Kretchy 2020	34% [27%, 41%]
142 / 422	Wondm 2024	34% [29%, 38%]
136 / 400	Tefera 2020	34% [29%, 39%]
116 / 321	Araya 2020	36% [31%, 42%]
115 / 309	Kassahun 2016a (N=309)	37% [32%, 43%]
77 / 200	Bruce 2015	38% [32%, 46%]
158 / 404	Owolabi 2024	39% [34%, 44%]
82 / 200	Yusuff 2008	41% [34%, 48%]
32 / 75	Ojieabu 2017	43% [31%, 55%]
131 / 289	Waari 2018	45% [39%, 51%]
89 / 195	Aminde 2019	46% [39%, 53%]
141 / 305	Dimore 2023	46% [41%, 52%]
93 / 200	Murwanashyaka 2022	46% [39%, 54%]
86 / 176	Adisa 2013	49% [41%, 56%]
170 / 347	Wabe 2011	49% [44%, 54%]
193 / 376	Lalam 2026	51% [46%, 56%]
91 / 172	Adisa 2014	53% [45%, 61%]
110 / 201	Ipingbemi 2021	55% [48%, 62%]
103 / 188	Aje 2024	55% [47%, 62%]
80 / 146	Ali 2017	55% [46%, 63%]
169 / 305	Hadush 2024	55% [50%, 61%]
153 / 270	Mwangasha 2021	57% [51%, 63%]
108 / 182	Tewabe 2018	59% [52%, 67%]
69 / 114	Adisa 2011	61% [51%, 70%]
252 / 411	Zemen 2025	61% [56%, 66%]
118 / 191	Boshe 2021b (N=191)	62% [54%, 69%]
186 / 300	Ibrahim 2021	62% [56%, 68%]
124 / 200	Adisa 2017	62% [55%, 69%]
157 / 248	Doya 2024	63% [57%, 69%]
148 / 228	Fekadu 2019	65% [58%, 71%]
126 / 191	Boshe 2021a (N=191)	66% [59%, 73%]
265 / 396	Mutyambizi 2020	67% [62%, 72%]
125 / 186	Sefah 2025	67% [60%, 74%]
196 / 285	Kassahun 2016b (N=285)	69% [63%, 74%]
49 / 71	Tarwirei 2013	69% [57%, 79%]
137 / 196	Adegbola 2016	70% [63%, 76%]
194 / 275	Ayele 2019b (N=275)	71% [65%, 76%]
562 / 794	Simegn 2023	71% [67%, 74%]
153 / 215	Rwegerera 2014	71% [65%, 77%]
258 / 359	Tusa 2020	72% [67%, 76%]
105 / 145	Omotosho 2024	72% [64%, 80%]
87 / 120	Pascal 2012	72% [64%, 80%]
268 / 357	Demoz 2020	75% [70%, 79%]
302 / 402	Zwane 2023	75% [71%, 79%]
247 / 327	AT Kassaw 2025	76% [71%, 80%]
202 / 267	Teklay 2013	76% [70%, 81%]
67 / 87	Billoro 2022	77% [67%, 85%]
261 / 332	Edmealem 2020	79% [74%, 83%]
305 / 371	Mekonnen 2021	82% [78%, 86%]
289 / 348	Enikuomehin 2021	83% [79%, 87%]
434 / 521	Bagonza 2015	83% [80%, 86%]
251 / 300	Berhe 2017	84% [79%, 88%]
163 / 194	Sorato 2016	84% [78%, 89%]
279 / 330	Afaya 2020	85% [80%, 88%]
155 / 178	Sodipo 2021	87% [81%, 92%]
51 / 58	Gurmu 2023	88% [77%, 95%]
125 / 140	Bofu 2024	89% [83%, 94%]
288 / 320	Ayele 2019a (N=320)	90% [86%, 93%]
95 / 102	Woldu 2014	93% [86%, 97%]
378 / 403	Gulentie 2020	94% [91%, 96%]
404 / 422	Bonger 2018	96% [93%, 97%]
144 / 150	Sookan 2022	96% [91%, 99%]
351 / 363	Alenbalu 2024	97% [94%, 98%]
311 / 320	Berhe 2012	97% [95%, 99%]
91 / 93	Gemeda 2022	98% [92%, 100%]
207 / 207	Degefa 2020	100% [98%, 100%]
	**Pooled**	**67% [60%, 73%] I^2 = 99% (k = 73)**

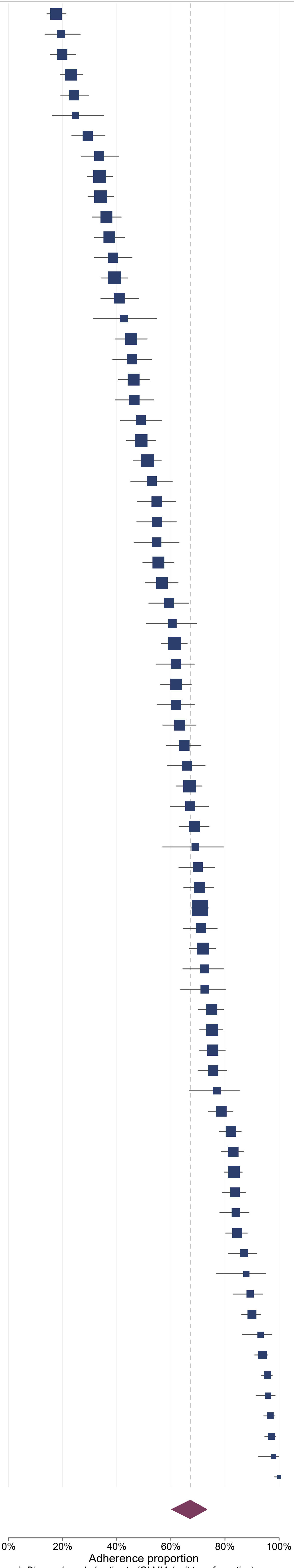

Squares: study estimates (size ∝ inverse SE). Horizontal lines: 95% CI (Clopper–Pearson). Diamond: pooled estimate (GLMM, logit transformation).

Appendix 3b. Anti-hypertensive medications

Random-effects meta-analysis | k = 87 studies | Total N = 31,072 | Events = 15,577

Events / N	Study	Proportion [95% CI]
5 / 500	Oluwole 2019	1% [0%, 2%]
6 / 400	Makukule 2023	2% [1%, 3%]
10 / 329	Okello 2016	3% [1%, 6%]
6 / 148	Adeoye 2019	4% [2%, 9%]
27 / 400	Kretchy 2014 (Validated scale)	7% [4%, 10%]
24 / 284	Onyinye 2021	8% [5%, 12%]
54 / 605	Adisa 2018	9% [7%, 11%]
52 / 378	Desta 2024	14% [10%, 18%]
19 / 112	Mugwano 2016	17% [11%, 25%]
64 / 358	Kretchy 2020	18% [14%, 22%]
194 / 925	Berhe 2017	21% [18%, 24%]
91 / 335	Mebrahtu 2021	27% [22%, 32%]
80 / 252	Okwuonu 2015	32% [26%, 38%]
59 / 183	Adidja 2018	32% [26%, 40%]
119 / 357	Boima 2015	33% [28%, 38%]
32 / 96	Otenyo 2018	33% [24%, 44%]
782 / 2198	MacquartdeTerline 2019	36% [34%, 38%]
356 / 989	Tsadik 2020	36% [33%, 39%]
119 / 330	Fekadu 2020	36% [31%, 41%]
153 / 422	Onwukwe 2022	36% [32%, 41%]
145 / 399	Atibila 2022	36% [32%, 41%]
156 / 422	Abdisa 2022 (Validated scale)	37% [32%, 42%]
41 / 102	Mukora-Mutseyekwa 2013	40% [31%, 50%]
130 / 322	Abdulwahed 2020	40% [35%, 46%]
112 / 277	Attahiru 2023	40% [35%, 46%]
109 / 269	Osei-Yeboah 2019	41% [35%, 47%]
98 / 241	Weldegebreal 2019	41% [34%, 47%]
281 / 678	Obirikorang 2018	41% [38%, 45%]
155 / 370	Adebolu 2014	42% [37%, 47%]
97 / 221	Akoko 2017	44% [37%, 51%]
33 / 74	Mbaseege 2025	45% [33%, 57%]
181 / 395	Lulebo 2015	46% [41%, 51%]
86 / 187	Adegoke 2022	46% [39%, 53%]
191 / 410	Simegn 2021	47% [42%, 52%]
215 / 450	Kebede 2024	48% [43%, 53%]
58 / 120	Nashilongo 2017	48% [39%, 58%]
198 / 405	Tebelu 2023	49% [44%, 54%]
196 / 400	Yusuff 2007	49% [44%, 54%]
212 / 422	Abdisa 2022 (H-SCALE)	50% [45%, 55%]
224 / 440	Osamor 2011	51% [46%, 56%]
224 / 440	Osamor 2015	51% [46%, 56%]
219 / 422	Melaku 2022	52% [47%, 57%]
190 / 366	Andualem 2021	52% [47%, 57%]
81 / 153	Tamuno 2012	53% [45%, 61%]
137 / 251	Rampamba 2018	55% [48%, 61%]
266 / 480	Assefa 2023	55% [51%, 60%]
168 / 300	Maginga 2016	56% [50%, 62%]
53 / 93	Xiong 2023	57% [46%, 67%]
103 / 180	Onwukwe 2012	57% [50%, 65%]
145 / 250	Adisa 2017	58% [52%, 64%]
120 / 201	Mungati 2014	60% [53%, 67%]
214 / 357	Yousuf 2025	60% [55%, 65%]
60 / 100	Akpa 2005	60% [50%, 70%]
173 / 286	Asgedom 2016	60% [55%, 66%]
257 / 420	Ayodapo 2020	61% [56%, 66%]
173 / 280	Asgedom 2018	62% [56%, 68%]
235 / 380	DiendÃ©rÃ© 2024	62% [57%, 67%]
211 / 341	Labata 2019	62% [56%, 67%]
216 / 348	Olowe 2017	62% [57%, 67%]
255 / 402	Rashid 2024	63% [59%, 68%]
225 / 351	Amadu 2025	64% [59%, 69%]
45 / 70	Thinyane 2015	64% [52%, 75%]
248 / 384	Ambaw 2012	65% [60%, 69%]
161 / 249	Gebreyohannes 2019	65% [58%, 71%]
65 / 100	Peltzer 2004	65% [55%, 74%]
275 / 409	Mekonnen 2017	67% [62%, 72%]
198 / 286	Hareri 2013	69% [64%, 75%]
160 / 223	Kinfe 2020	72% [65%, 78%]
319 / 440	Menanga 2016	72% [68%, 77%]
285 / 393	Elias 2025	73% [68%, 77%]
293 / 396	Aberhe 2020	74% [69%, 78%]
237 / 320	Gebremichael 2019	74% [69%, 79%]
195 / 258	Ike 2010	76% [70%, 81%]
281 / 370	Muhe 2025	76% [71%, 80%]
86 / 112	Sibomana 2019	77% [68%, 84%]
653 / 849	Pallangyo 2022	77% [74%, 80%]
303 / 392	Teshome 2018	77% [73%, 81%]
78 / 100	Omolase 2011	78% [69%, 86%]
136 / 172	Berisa 2018	79% [72%, 85%]
235 / 284	Wahab 2017	83% [78%, 87%]
96 / 115	Wake 2021	83% [75%, 90%]
195 / 229	Kimani 2019	85% [80%, 89%]
351 / 400	Nakwafila 2022	88% [84%, 91%]
330 / 370	Sarkodie 2020	89% [86%, 92%]
332 / 360	Fentaw 2022	92% [89%, 95%]
738 / 785	Kuria 2018	94% [92%, 96%]
387 / 400	Okunrinboye 2019	97% [95%, 98%]
	**Pooled**	**51% [44%, 58%] I² = 99% (k = 87)**

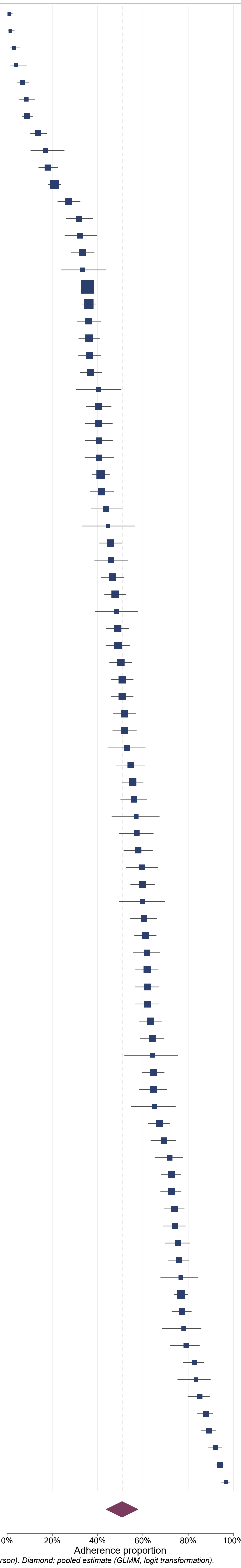

Squares: study estimates (size ∝ inverse SE). Horizontal lines: 95% CI (Clopper–Pearson). Diamond: pooled estimate (GLMM, logit transformation).

Appendix 3c. Dietary recommendations

Random-effects meta-analysis | k = 48 studies | Total N = 18,184 | Events = 8,049

Events / N	Study	Proportion [95% CI]
36 / 480	Assefa 2023	8% [5%, 10%]
38 / 422	Melaku 2022	9% [6%, 12%]
38 / 398	Duodu 2025	10% [7%, 13%]
39 / 191	Boshe 2021	20% [15%, 27%]
101 / 419	Bonger 2018	24% [20%, 28%]
100 / 399	Owolabi 2024	25% [21%, 30%]
82 / 320	Ayele 2018	26% [21%, 31%]
113 / 399	Abera 2024	28% [24%, 33%]
99 / 348	Enikuomehin 2021	28% [24%, 34%]
92 / 300	Berhe 2017	31% [25%, 36%]
127 / 403	Gulentie 2020	32% [27%, 36%]
74 / 228	Fekadu 2019	32% [26%, 39%]
127 / 386	Feleke 2022	33% [28%, 38%]
150 / 421	Tirfessa 2023	36% [31%, 40%]
215 / 600	Hailu 2025	36% [32%, 40%]
151 / 421	Abose 2024	36% [31%, 41%]
144 / 401	Demilew 2018	36% [31%, 41%]
48 / 130	Adje 2022	37% [29%, 46%]
115 / 307	Adem 2020	37% [32%, 43%]
172 / 406	Saliya 2024	42% [38%, 47%]
150 / 354	Jemal 2022	42% [37%, 48%]
169 / 387	Bekele 2024	44% [39%, 49%]
189 / 411	Zemen 2025	46% [41%, 51%]
388 / 840	Zeleke 2025	46% [43%, 50%]
149 / 322	Erkocho 2022	46% [41%, 52%]
197 / 422	Belay 2021	47% [42%, 52%]
203 / 422	Zinab 2019	48% [43%, 53%]
278 / 576	Mekonnen 2021	48% [44%, 52%]
284 / 570	MolalignTakele 2021	50% [46%, 54%]
72 / 142	Wilson 2024	51% [42%, 59%]
413 / 794	Simegn 2023	52% [48%, 56%]
185 / 354	Wolderufael 2021	52% [47%, 58%]
113 / 207	Degefa 2020	55% [48%, 62%]
180 / 328	Zewdie 2022	55% [49%, 60%]
290 / 497	Belew 2025	58% [54%, 63%]
209 / 352	Worsa 2021	59% [54%, 65%]
116 / 194	Sorato 2016	60% [53%, 67%]
226 / 364	Muhe 2025	62% [57%, 67%]
246 / 396	Mutyambizi 2020	62% [57%, 67%]
255 / 406	Atinafu 2025	63% [58%, 68%]
202 / 320	Gebremichael 2019	63% [58%, 68%]
206 / 322	ZelekeNegera 2020	64% [58%, 69%]
60 / 93	Gemeda 2022	65% [54%, 74%]
185 / 286	Hareri 2013	65% [59%, 70%]
272 / 398	Gebermariam 2020	68% [64%, 73%]
279 / 404	Tibebu 2017	69% [64%, 74%]
242 / 343	Atosona 2024	71% [65%, 75%]
230 / 301	Andualem 2020	76% [71%, 81%]
	**Pooled**	**44% [38%, 49%] I^2 = 98% (k = 48)**

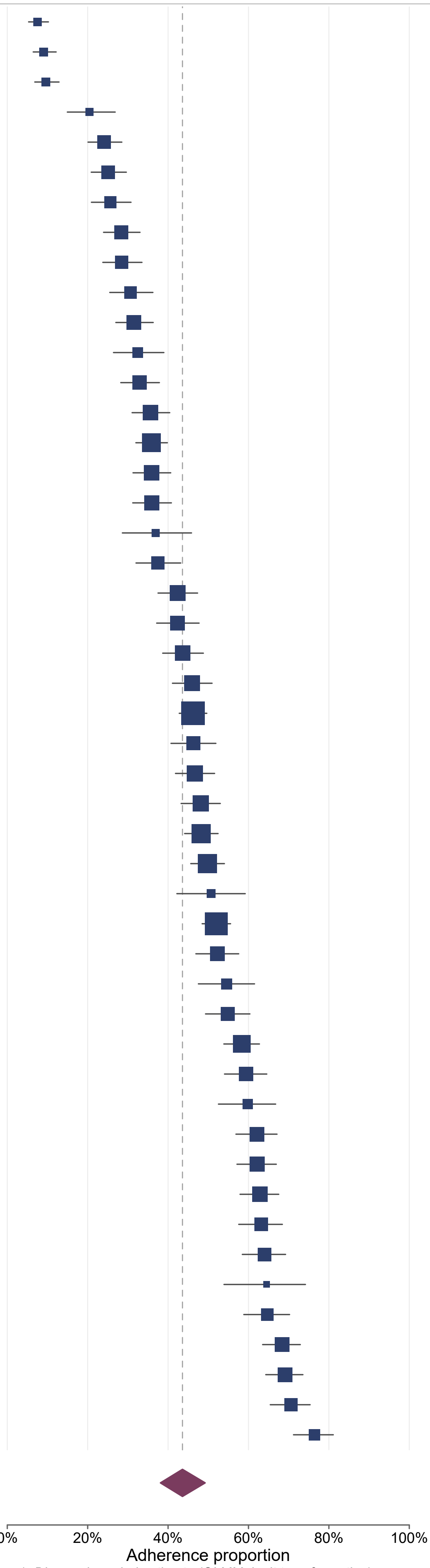

Squares: study estimates (size ∝ inverse SE). Horizontal lines: 95% CI (Clopper–Pearson). Diamond: pooled estimate (GLMM, logit transformation).

Appendix 3d. Physical activity recommendations

Random-effects meta-analysis | k = 65 studies | Total N = 22,438 | Events = 9,589

Events / N	Study	Proportion [95% CI]
36 / 396	Mutyambizi 2020	9% [6%, 12%]
44 / 406	Saliya 2024	11% [8%, 14%]
25 / 191	Boshe 2021	13% [9%, 19%]
54 / 402	Zwane 2023	13% [10%, 17%]
33 / 205	Buda 2017	16% [11%, 22%]
83 / 480	Assefa 2023	17% [14%, 21%]
54 / 305	Dimore 2023	18% [14%, 22%]
78 / 398	Duodu 2025	20% [16%, 24%]
81 / 384	WorkuKassahun 2020	21% [17%, 26%]
68 / 314	Kitaw 2023	22% [17%, 27%]
31 / 140	Bofu 2024	22% [16%, 30%]
99 / 400	Makukule 2023	25% [21%, 29%]
105 / 422	Melaku 2022	25% [21%, 29%]
125 / 422	Abdisa 2022	30% [25%, 34%]
121 / 391	Hussen 2020	31% [26%, 36%]
110 / 350	Tozivepi 2021	31% [27%, 37%]
127 / 404	Tibebu 2017	31% [27%, 36%]
128 / 399	Owolabi 2024	32% [28%, 37%]
30 / 93	Gemeda 2022	32% [23%, 43%]
127 / 378	Desta 2024	34% [29%, 39%]
115 / 322	ZelekeNegera 2020	36% [30%, 41%]
120 / 328	Kindarara 2025	37% [31%, 42%]
118 / 311	Shilole 2024 [SURFACED]	38% [33%, 44%]
122 / 320	Ayele 2018	38% [33%, 44%]
81 / 207	Degefa 2020	39% [32%, 46%]
129 / 328	Zewdie 2022	39% [34%, 45%]
120 / 301	Andualem 2020	40% [34%, 46%]
161 / 403	Gulentie 2020	40% [35%, 45%]
39 / 97	Taylor 2023	40% [30%, 51%]
340 / 840	Zeleke 2025	40% [37%, 44%]
135 / 330	Philbert 2023	41% [36%, 46%]
246 / 600	Hailu 2025	41% [37%, 45%]
168 / 405	Tebelu 2023	41% [37%, 46%]
125 / 286	Hareri 2013	44% [38%, 50%]
176 / 398	Geremew 2023	44% [39%, 49%]
174 / 392	Teshome 2018	44% [39%, 49%]
128 / 286	Asgedom 2016	45% [39%, 51%]
153 / 341	Labata 2019	45% [40%, 50%]
124 / 276	Niriayo 2019	45% [39%, 51%]
77 / 170	Shiriyedeve 2019	45% [38%, 53%]
64 / 141	Night 2023	45% [37%, 54%]
188 / 411	Zemen 2025	46% [41%, 51%]
194 / 419	Bonger 2018	46% [41%, 51%]
158 / 320	Gebremichael 2019	49% [44%, 55%]
284 / 570	MolalignTakele 2021	50% [46%, 54%]
37 / 74	Mbaseege 2025	50% [38%, 62%]
98 / 194	Sorato 2016	51% [43%, 58%]
196 / 387	Bekele 2024	51% [46%, 56%]
181 / 354	Wolderufael 2021	51% [46%, 56%]
203 / 392	Gelaw 2021	52% [47%, 57%]
424 / 794	Simegn 2023	53% [50%, 57%]
47 / 84	Banson 2023	56% [45%, 67%]
300 / 497	Belew 2025	60% [56%, 65%]
262 / 422	Debalke 2022	62% [57%, 67%]
299 / 450	Kebede 2024	66% [62%, 71%]
222 / 332	Edmealem 2020	67% [62%, 72%]
214 / 320	Ayele 2019	67% [61%, 72%]
233 / 348	Enikuomehin 2021	67% [62%, 72%]
82 / 119	Oyewole 2014	69% [60%, 77%]
104 / 150	Osei-Yeboah 2019	69% [61%, 77%]
267 / 374	Mbambo 2019	71% [67%, 76%]
313 / 423	Oluma 2020 [SURFACED]	74% [70%, 78%]
222 / 300	Berhe 2017	74% [69%, 79%]
247 / 322	Abdulwahed 2020	77% [72%, 81%]
340 / 420	Belay 2023	81% [77%, 85%]
	**Pooled**	**42% [37%, 47%] I² = 98% (k = 65)**

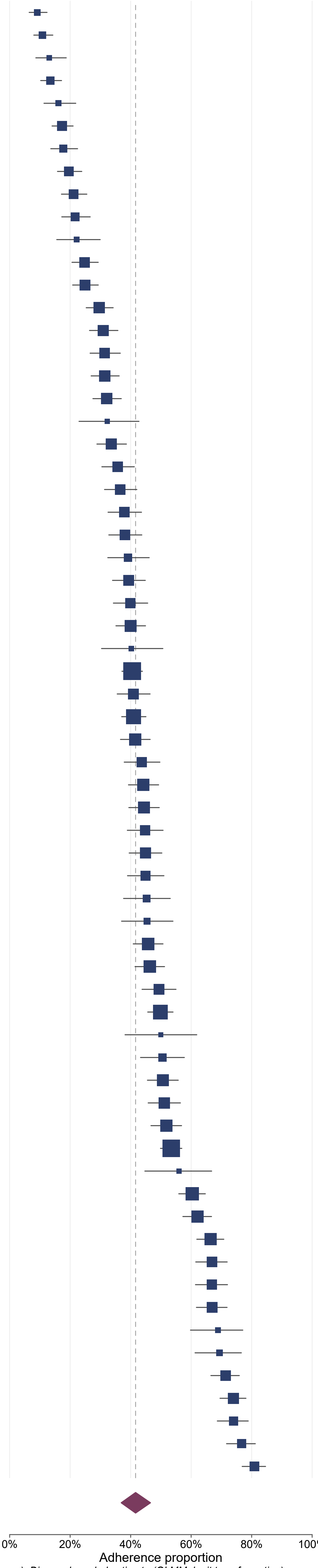

Squares: study estimates (size ∝ inverse SE). Horizontal lines: 95% CI (Clopper–Pearson). Diamond: pooled estimate (GLMM, logit transformation).

Appendix 3e. Alcohol recommendations

Random-effects meta-analysis | k = 72 studies | Total N = 26,546 | Events = 21,887

Events / N	Study	Proportion [95% CI]
43 / 121	Mosha 2009	36% [27%, 45%]
178 / 423	Belay 2023	42% [37%, 47%]
205 / 450	Kebede 2024	46% [41%, 50%]
91 / 156	Belayneh 2021	58% [50%, 66%]
245 / 400	Makukule 2023	61% [56%, 66%]
266 / 421	Gebreyesus 2021	63% [58%, 68%]
252 / 398	Geremew 2023	63% [58%, 68%]
244 / 380	Diendéré 2024	64% [59%, 69%]
257 / 392	Gelaw 2021	66% [61%, 70%]
269 / 402	Zwane 2023	67% [62%, 71%]
215 / 320	Gebremichael 2019	67% [62%, 72%]
284 / 422	Abdisa 2022	67% [63%, 72%]
69 / 102	Woldu 2014	68% [58%, 77%]
256 / 378	Desta 2024	68% [63%, 72%]
253 / 371	Mekonnen 2021 (N=371)	68% [63%, 73%]
190 / 276	Niriayo 2019	69% [63%, 74%]
278 / 384	WorkuKassahun 2020	72% [68%, 77%]
302 / 404	Tibebu 2017	75% [70%, 79%]
297 / 391	Aberhe 2020	76% [71%, 80%]
292 / 384	Emire 2022	76% [71%, 80%]
411 / 539	Hellebo 2024	76% [72%, 80%]
93 / 121	Ike 2010	77% [68%, 84%]
526 / 678	Obirikorang 2018	78% [74%, 81%]
264 / 335	Mebrahtu 2021	79% [74%, 83%]
312 / 391	Hussen 2020	80% [75%, 84%]
285 / 348	Enikuomehin 2021	82% [77%, 86%]
333 / 405	Tebelu 2023	82% [78%, 86%]
247 / 297	Fekadu 2020	83% [78%, 87%]
238 / 286	Asgedom 2016	83% [78%, 87%]
373 / 440	Hulzebosch 2015	85% [81%, 88%]
358 / 422	Melaku 2022	85% [81%, 88%]
340 / 399	Abera 2024	85% [81%, 89%]
269 / 315	Mwimo 2021	85% [81%, 89%]
212 / 248	Doya 2024	85% [80%, 90%]
351 / 405	Abose 2024	87% [83%, 90%]
198 / 228	Fekadu 2019	87% [82%, 91%]
199 / 229	Kimani 2019	87% [82%, 91%]
80 / 92	Olaitan 2018	87% [78%, 93%]
237 / 271	Hussen 2017	87% [83%, 91%]
267 / 305	Hadush 2024	88% [83%, 91%]
313 / 357	Demoz 2020	88% [84%, 91%]
301 / 341	Labata 2019	88% [84%, 91%]
99 / 112	Sibomana 2019	88% [81%, 94%]
354 / 400	Tefera 2020	88% [85%, 91%]
507 / 570	Molalign Takele 2021	89% [86%, 91%]
89 / 100	Katende-Kyenda 2025	89% [81%, 94%]
376 / 422	Belay 2021	89% [86%, 92%]
515 / 576	Mekonnen 2021 (N=576)	89% [87%, 92%]
374 / 418	Demoz 2019	89% [86%, 92%]
88 / 98	Asante 2024	90% [82%, 95%]
185 / 205	Buda 2017	90% [85%, 94%]
399 / 440	Osamor 2015	91% [88%, 93%]
320 / 352	Worsa 2021	91% [87%, 94%]
342 / 375	Feleke 2022	91% [88%, 94%]
286 / 312	Dawite 2023	92% [88%, 94%]
205 / 223	Patrick 2021	92% [88%, 95%]
316 / 343	Atosona 2024	92% [89%, 95%]
377 / 409	Shimels 2021	92% [89%, 95%]
2652 / 2870	Sarfo 2018	92% [91%, 93%]
734 / 785	Kuria 2018	94% [92%, 95%]
182 / 194	Sorato 2016	94% [89%, 97%]
283 / 301	Andualem 2020	94% [91%, 96%]
339 / 360	Fentaw 2022	94% [91%, 96%]
132 / 140	Odusola 2015	94% [89%, 98%]
307 / 325	Kassahun 2016	94% [91%, 97%]
133 / 140	Bofu 2024	95% [90%, 98%]
381 / 400	Gebreziher 2024	95% [93%, 97%]
307 / 322	Abdulwahed 2020	95% [92%, 97%]
103 / 107	Omoronyia 2021	96% [91%, 99%]
343 / 350	Tozivepi 2021	98% [96%, 99%]
392 / 396	Mutyambizi 2020	99% [97%, 100%]
74 / 74	Mbaseege 2025	100% [95%, 100%]
	**Pooled**	**85% [82%, 88%] I^2 = 98% (k = 72)**

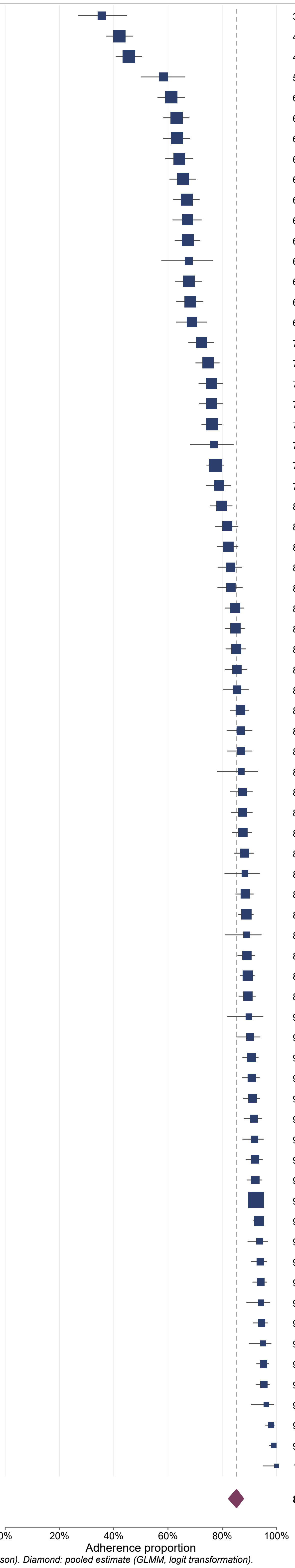

Squares: study estimates (size ∝ inverse SE). Horizontal lines: 95% CI (Clopper–Pearson). Diamond: pooled estimate (GLMM, logit transformation).

Appendix 3f. Smoking recommendations

Random-effects meta-analysis | k = 82 studies | Total N = 31,985 | Events = 29,670

Events / N	Study	Proportion [95% CI]
265 / 402	Zwane 2023	66% [61%, 71%]
272 / 384	WorkuKassahun 2020	71% [66%, 75%]
300 / 411	Zemen 2025	73% [68%, 77%]
407 / 539	Hellebo 2024	76% [72%, 79%]
320 / 391	Hussen 2020	82% [78%, 86%]
86 / 102	Woldu 2014	84% [76%, 91%]
319 / 378	Desta 2024	84% [80%, 88%]
338 / 400	Makukule 2023	84% [81%, 88%]
337 / 396	Mutyambizi 2020	85% [81%, 88%]
222 / 260	Ike 2010	85% [80%, 89%]
362 / 422	Abdisa 2022	86% [82%, 89%]
357 / 416	Tibebu 2017	86% [82%, 89%]
277 / 322	ZelekeNegera 2020	86% [82%, 90%]
148 / 172	Berisa 2018	86% [80%, 91%]
333 / 384	Emire 2022	87% [83%, 90%]
236 / 271	Hussen 2017	87% [82%, 91%]
331 / 380	Diendéré 2024	87% [83%, 90%]
88 / 101	Nkosi 2010	87% [79%, 93%]
198 / 226	Jackson 2021	88% [83%, 92%]
350 / 399	Abera 2024	88% [84%, 91%]
350 / 398	Geremew 2023	88% [84%, 91%]
740 / 840	Zeleke 2025	88% [86%, 90%]
328 / 371	Mekonnen 2021 (N=371)	88% [85%, 91%]
270 / 301	Andualem 2020	90% [86%, 93%]
248 / 276	Niriayo 2019	90% [86%, 93%]
67 / 74	Mbaseege 2025	91% [81%, 96%]
369 / 405	Tebelu 2023	91% [88%, 94%]
192 / 210	Buda 2017	91% [87%, 95%]
403 / 440	Hulzebosch 2015	92% [89%, 94%]
210 / 229	Kimani 2019	92% [87%, 95%]
623 / 678	Obirikorang 2018	92% [90%, 94%]
322 / 348	Enikuomehin 2021	93% [89%, 95%]
900 / 968	Berhe 2017	93% [91%, 95%]
213 / 228	Fekadu 2019	93% [89%, 96%]
319 / 341	Labata 2019	94% [90%, 96%]
395 / 422	Melaku 2022	94% [91%, 96%]
396 / 422	Mekonnen 2020	94% [91%, 96%]
287 / 305	Hadush 2024	94% [91%, 96%]
106 / 112	Sibomana 2019	95% [89%, 98%]
236 / 249	Gebreyohannes 2019	95% [91%, 97%]
2086 / 2198	Cavagna 2021	95% [94%, 96%]
95 / 100	Katende-Kyenda 2025	95% [89%, 98%]
429 / 450	Kebede 2024	95% [93%, 97%]
143 / 149	Odusola 2018	96% [91%, 99%]
382 / 398	Gebermariam 2020	96% [94%, 98%]
406 / 423	Belay 2023	96% [94%, 98%]
242 / 252	Mogre 2020	96% [93%, 98%]
346 / 360	Fentaw 2022	96% [94%, 98%]
330 / 343	Atosona 2024	96% [94%, 98%]
103 / 107	Omoronyia 2021	96% [91%, 99%]
323 / 335	Mebrahtu 2021	96% [94%, 98%]
386 / 400	Gebreziher 2024	96% [94%, 98%]
425 / 440	Osamor 2015	97% [94%, 98%]
464 / 480	Assefa 2023	97% [95%, 98%]
117 / 121	Mosha 2009	97% [92%, 99%]
277 / 286	Asgedom 2016	97% [94%, 99%]
339 / 350	Tozivepi 2021	97% [94%, 98%]
761 / 785	Kuria 2018	97% [95%, 98%]
300 / 309	Kassahun 2016	97% [95%, 99%]
306 / 315	Mwimo 2021	97% [95%, 99%]
241 / 248	Doya 2024	97% [94%, 99%]
313 / 322	Abdulwahed 2020	97% [95%, 99%]
319 / 328	Kindarara 2025	97% [95%, 99%]
389 / 399	Gelaw 2021	97% [95%, 99%]
395 / 405	Abose 2024	98% [96%, 99%]
213 / 218	Ndege 2014	98% [95%, 99%]
563 / 576	Mekonnen 2021 (N=576)	98% [96%, 99%]
218 / 223	Patrick 2021	98% [95%, 99%]
414 / 422	Belay 2021	98% [96%, 99%]
314 / 320	Berhe 2012	98% [96%, 99%]
184 / 187	Mogre 2017	98% [95%, 100%]
385 / 391	Aberhe 2020	98% [97%, 99%]
138 / 140	Bofu 2024	99% [95%, 100%]
387 / 392	Teshome 2018	99% [97%, 100%]
91 / 92	Olaitan 2018	99% [94%, 100%]
327 / 330	Fekadu 2020	99% [97%, 100%]
354 / 357	Demoz 2020	99% [98%, 100%]
355 / 357	Demoz 2019	99% [98%, 100%]
2856 / 2870	Sarfo 2018	100% [99%, 100%]
194 / 194	Sorato 2016	100% [98%, 100%]
100 / 100	Oguntibeju 2012	100% [96%, 100%]
140 / 140	Lopez 2025	100% [97%, 100%]
	**Pooled**	**95% [94%, 96%] I^2 = 96% (k = 82)**

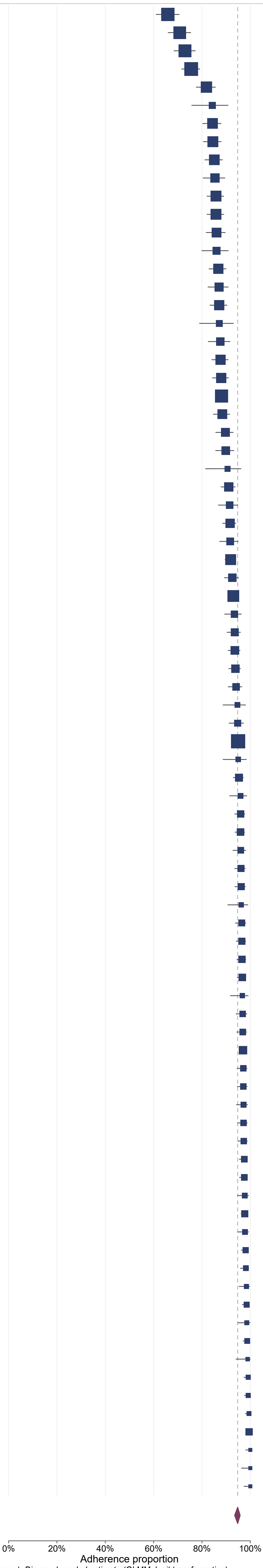

Squares: study estimates (size ∝ inverse SE). Horizontal lines: 95% CI (Clopper–Pearson). Diamond: pooled estimate (GLMM, logit transformation).

Appendix 3g. Blood glucose self-monitoring

Random-effects meta-analysis | k = 39 studies | Total N = 12,508 | Events = 3,099

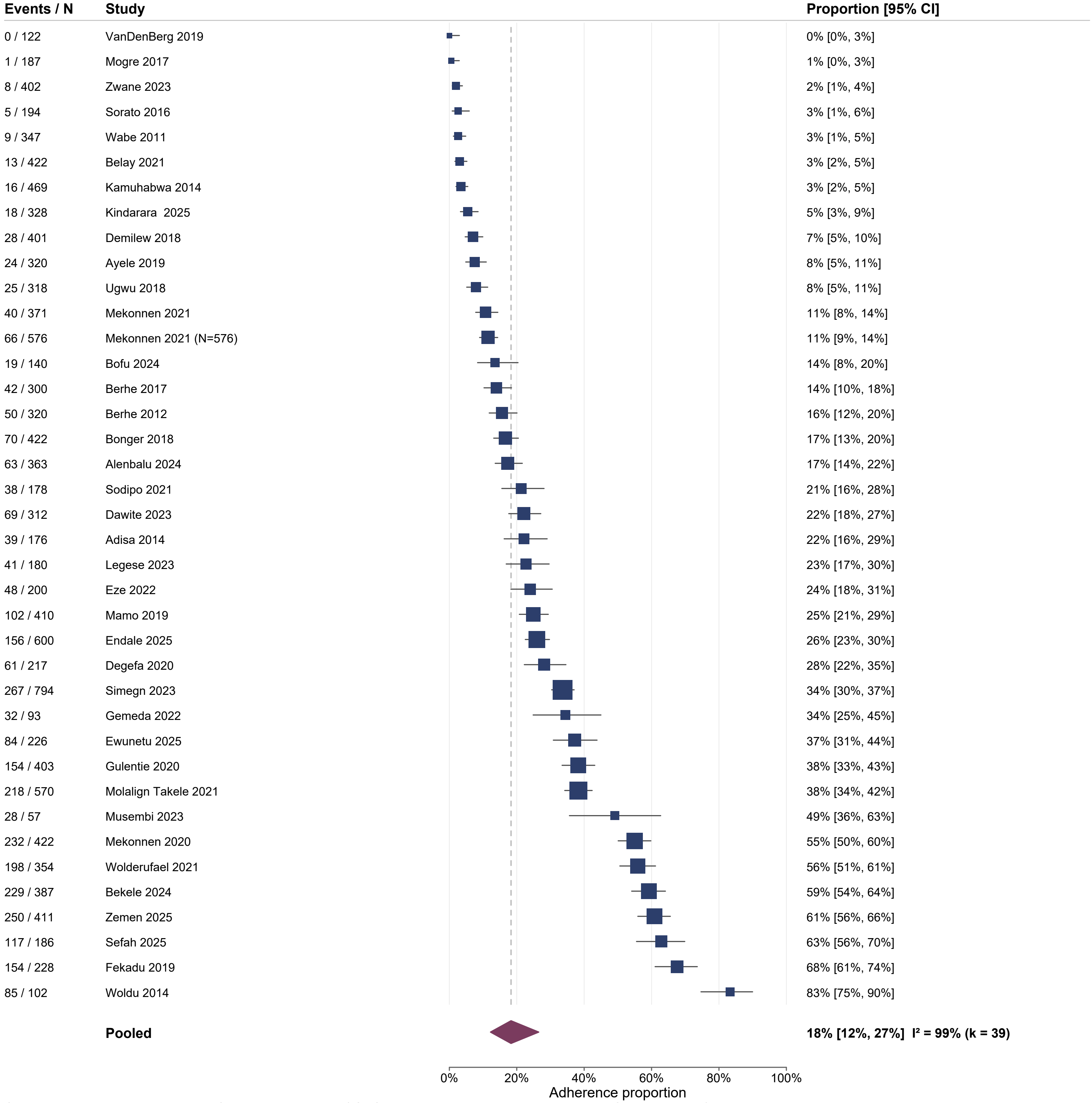

Squares: study estimates (size ∝ inverse SE). Horizontal lines: 95% CI (Clopper–Pearson). Diamond: pooled estimate (GLMM, logit transformation).

Appendix 3h. Blood pressure self-monitoring

Random-effects meta-analysis | k = 8 studies | Total N = 2,354 | Events = 616

Events / N	Study	Proportion [95% CI]
31 / 400	Wake 2020	8% [5%, 11%]
30 / 336	Edmealem 2023	9% [6%, 13%]
80 / 322	Abdulwahed 2020	25% [20%, 30%]
108 / 400	Gebreziher 2024	27% [23%, 32%]
92 / 330	Philbert 2023	28% [23%, 33%]
118 / 281	Amorha 2019	42% [36%, 48%]
78 / 178	Sodipo 2021	44% [36%, 51%]
79 / 107	Omoronyia 2021	74% [64%, 82%]
	**Pooled**	**28% [16%, 45%] I² = 98% (k = 8)**

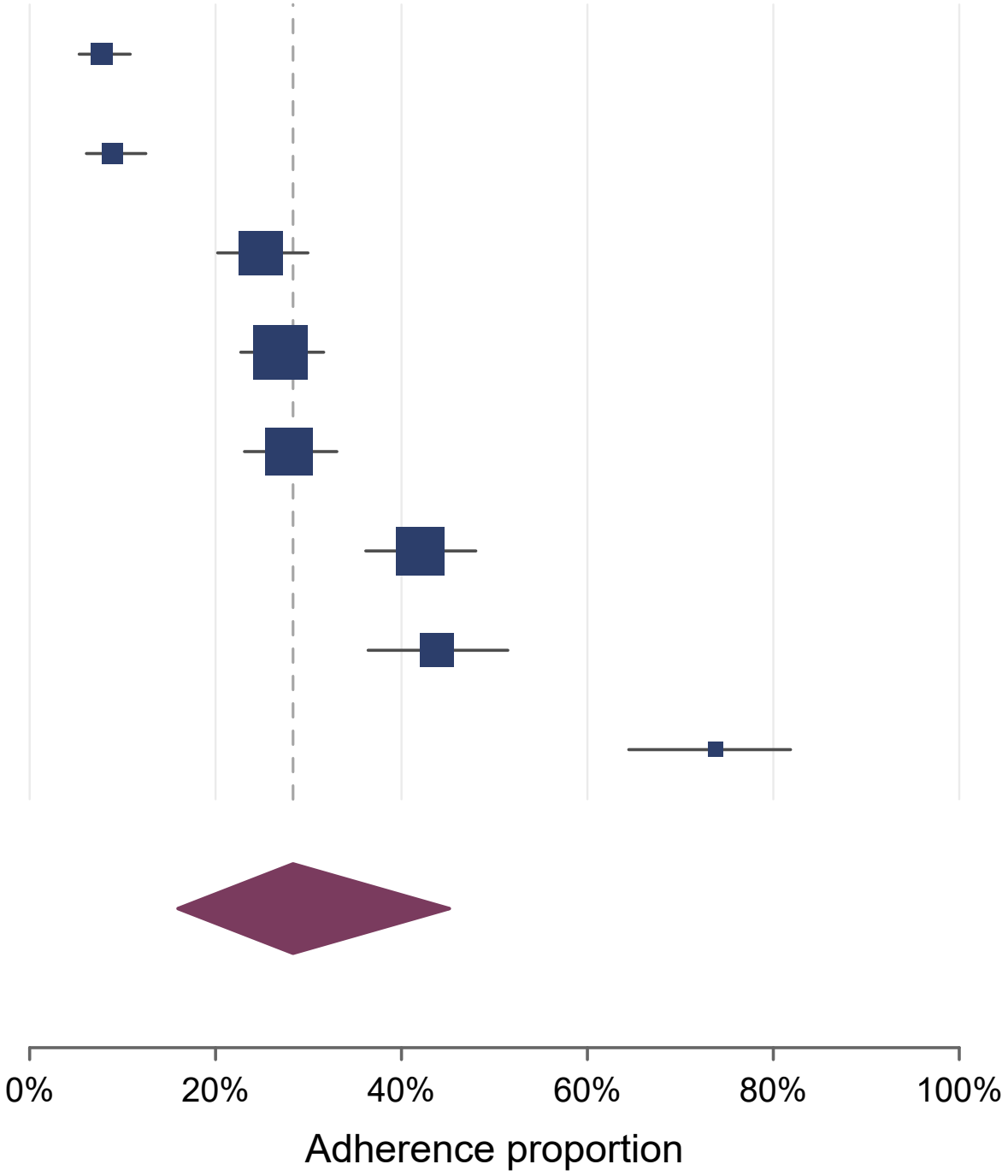

Squares: study estimates (size ∝ inverse SE). Horizontal lines: 95% CI (Clopper–Pearson). Diamond: pooled estimate (GLMM, logit transformation).

Appendix 3i. Overall diabetes self-care

Random-effects meta-analysis | k = 31 studies | Total N = 11,350 | Events = 4,610

Events / N	Study	Proportion [95% CI]
5 / 217	Okonta 2014	2% [1%, 5%]
20 / 402	Zwane 2023	5% [3%, 8%]
12 / 140	Bofu 2024	9% [5%, 14%]
63 / 539	Hellebo 2024	12% [9%, 15%]
51 / 422	Wondm 2024	12% [9%, 16%]
73 / 345	Miyashita 2024	21% [17%, 26%]
240 / 726	Kiarie 2023	33% [30%, 37%]
106 / 284	Mariye 2018	37% [32%, 43%]
122 / 320	Ayele 2019	38% [33%, 44%]
152 / 384	Emire 2022	40% [35%, 45%]
172 / 422	Mekonnen 2020	41% [36%, 46%]
80 / 194	Sorato 2016	41% [34%, 49%]
337 / 794	Simegn 2023	42% [39%, 46%]
262 / 600	Endale 2025	44% [40%, 48%]
149 / 328	Kindarara 2025	45% [40%, 51%]
34 / 74	Tarwirei 2013	46% [34%, 58%]
178 / 387	Bekele 2024	46% [41%, 51%]
266 / 570	MolalignTakele 2021	47% [43%, 51%]
99 / 207	Degefa 2020	48% [41%, 55%]
178 / 371	Jemal 2022	48% [43%, 53%]
170 / 354	Wolderufael 2021	48% [43%, 53%]
152 / 309	Kassahun 2016	49% [43%, 55%]
29 / 58	Gurmu 2023	50% [37%, 63%]
173 / 344	Zewdie 2022	50% [45%, 56%]
153 / 300	Berhe 2017	51% [45%, 57%]
201 / 371	Mekonnen 2021	54% [49%, 59%]
140 / 257	Gurmu 2018	54% [48%, 61%]
249 / 432	Eshete 2023	58% [53%, 62%]
244 / 411	Zemen 2025	59% [54%, 64%]
243 / 385	Tiruneh 2019	63% [58%, 68%]
257 / 403	Gulentie 2020	64% [59%, 68%]
	**Pooled**	**37% [30%, 46%] I² = 99% (k = 31)**

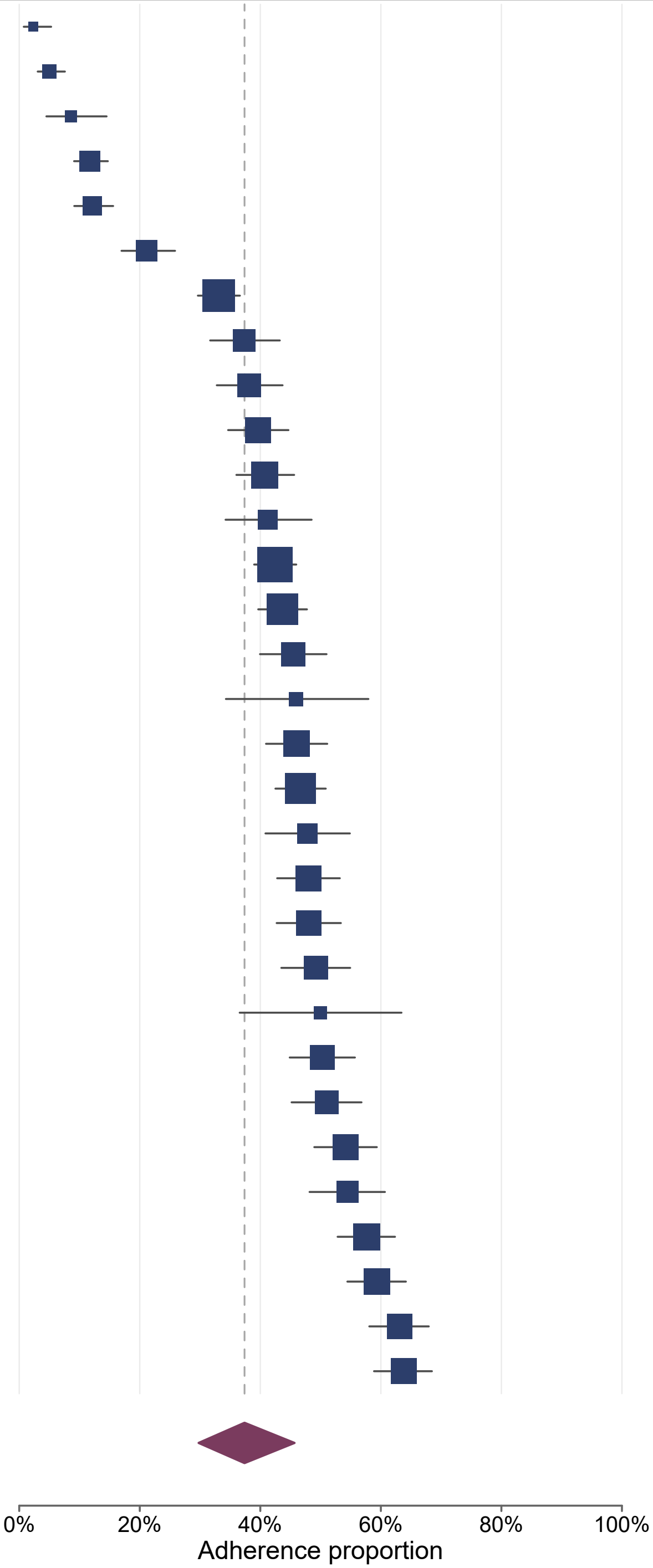

Squares: study estimates (size ∝ inverse SE). Horizontal lines: 95% CI (Clopper–Pearson). Diamond: pooled estimate (GLMM, logit transformation).

Appendix 3j. Overall hypertension self-care

Random-effects meta-analysis | k = 30 studies | Total N = 10,121 | Events = 3,655

Events / N	Study	Proportion [95% CI]
18 / 350	Tozivepi 2021	5% [3%, 8%]
90 / 762	Salawu 2024	12% [10%, 14%]
27 / 224	Kimani 2019	12% [8%, 17%]
63 / 330	Philbert 2023	19% [15%, 24%]
65 / 320	Gebremichael 2019	20% [16%, 25%]
96 / 416	Tibebu 2017	23% [19%, 27%]
71 / 301	Andualem 2020	24% [19%, 29%]
77 / 300	Maginga 2016	26% [21%, 31%]
57 / 210	Buda 2017	27% [21%, 34%]
109 / 370	Muhe 2025	29% [25%, 34%]
117 / 391	Hussen 2020	30% [25%, 35%]
129 / 398	Geremew 2023	32% [28%, 37%]
38 / 115	Wake 2021	33% [25%, 42%]
134 / 405	Tebelu 2023	33% [29%, 38%]
189 / 567	Miyashita 2024 (HTN)	33% [29%, 37%]
27 / 74	Mbaseege 2025	36% [26%, 48%]
157 / 405	Kebede 2022	39% [34%, 44%]
58 / 140	Odusola 2015	41% [33%, 50%]
51 / 120	Nashilongo 2017	42% [34%, 52%]
144 / 322	Abdulwahed 2020	45% [39%, 50%]
198 / 422	Melaku 2022	47% [42%, 52%]
219 / 450	Kebede 2024	49% [44%, 53%]
151 / 309	Ademe 2019	49% [43%, 55%]
235 / 480	Assefa 2023	49% [44%, 54%]
219 / 422	Abdisa 2022	52% [47%, 57%]
212 / 392	Gelaw 2021	54% [49%, 59%]
244 / 413	Gebresilase 2024	59% [54%, 64%]
228 / 384	WorkuKassahun 2020	59% [54%, 64%]
153 / 222	Fetensa 2019	69% [62%, 75%]
79 / 107	Omoronyia 2021	74% [64%, 82%]
	**Pooled**	**35% [29%, 42%] I² = 98% (k = 30)**

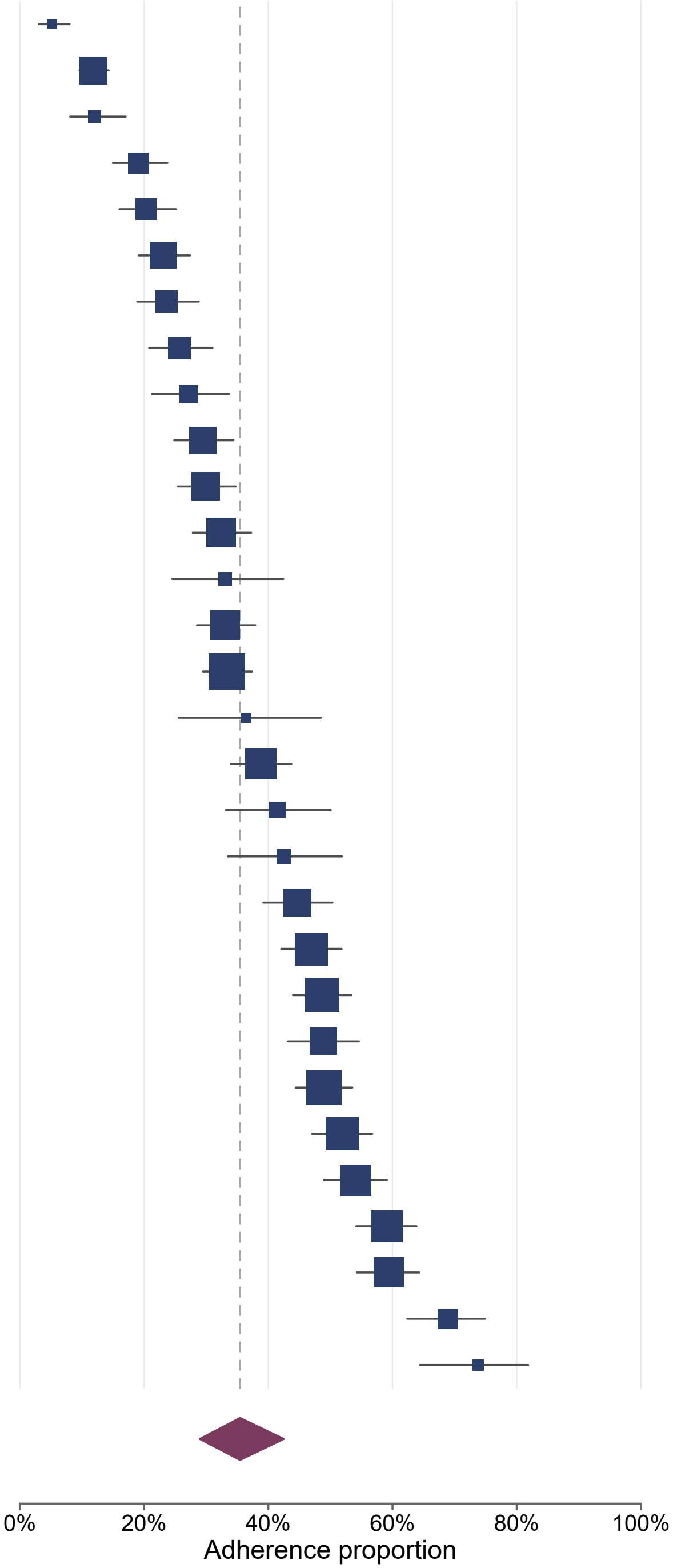

Squares: study estimates (size ∝ inverse SE). Horizontal lines: 95% CI (Clopper–Pearson). Diamond: pooled estimate (GLMM, logit transformation).
