# Supplementary material for: Adherence to medications, lifestyle advice, and self-monitoring for type 2 diabetes and hypertension in sub-Saharan Africa: A systematic review, meta-analysis, and interactive network analysis
Source: PLoS Med. 2026 Aug 3;23(8):e1005189. doi: 10.1371/journal.pmed.1005189 (PMC13460740; doi:10.1371/journal.pmed.1005189)
Supplement: Supplementary file 3 [file pmed.1005189.s004.pdf]

Appendix 4. Summary of subgroup results for adherence to medications

Adherence subgroup	No. of studies	Participants (N)	Pooled proportion (Random) % (95% CI)	Heterogeneity (I^2) %	P-value for heterogeneity
**Anti-diabetic medications (pooled adherence)**					
Mean age in years					
Age <50 years	08	2,283	70 (56–81)	97	< 0.001
Age ≥50 years	42	11,233	70 (62–78)	99	< 0.001
Percentage of females					
Females <50%	03	247	57 (30–81)	94	< 0.001
Females ≥50%	16	2,792	69 (55–79)	98	< 0.001
Rural vs urban residence					
Urban residence	39	8,602	66 (57–75)	98	< 0.001
Rural residence	03	1,011	57 (42–71)	95	< 0.001
Mixed residence	31	9,703	69 (59–78)	99	< 0.001
Geographical region					
West Africa	20	3,939	56 (46–65)	97	< 0.001
East Africa	45	13,200	70 (61–78)	99	< 0.001
Central/Southern Africa	08	2,177	76 (56–89)	99	< 0.001
Literature timepoint					
Year ≤2014	12	2,573	67 (49–80)	98	< 0.001
Year ≥2015	60	16,416	67 (59–74)	99	< 0.001
**Anti-hypertensive medications (pooled adherence)**					
Mean age in years					
Age <50 years	11	4,454	55 (38–71)	99	< 0.001
Age ≥50 years	59	21,502	48 (40–57)	99	< 0.001
Percentage of females					
Females <50%	21	7,946	48 (36–60)	99	< 0.001
Females ≥50%	58	20,310	50 (41–60)	99	< 0.001
Rural vs urban residence					
Urban residence	39	12,527	51 (40–62)	99	< 0.001
Rural residence	08	1,996	46 (26–68)	99	< 0.001
Mixed residence	40	16,549	51 (42–60)	99	< 0.001

Adherence subgroup	No. of studies	Participants (N)	Pooled proportion (Random) % (95% CI)	Heterogeneity (I^2) %	P-value for heterogeneity
Geographical region					
West Africa	28	11,258	43 (29–57)	99	< 0.001
East Africa	43	15,611	57 (49–65)	99	< 0.001
Central/Southern Africa	16	4,203	49 (34–63)	99	< 0.001
Literature timepoint					
Year ≤2014	15	3,852	51 (38–63)	98	< 0.001
Year ≥2015	72	27,220	51 (43–59)	99	< 0.001

Subgroup analyses of pooled adherence to anti-diabetic and anti-hypertensive medications. Results are presented as pooled proportions (random-effects generalized linear mixed models, GLMM) with 95% confidence intervals (CI). Heterogeneity was assessed using the I^2 statistic, with corresponding p-values for heterogeneity tests. Geographic region was established using the WHO AFRO classification.
