# Supplementary material for: Adherence to medications, lifestyle advice, and self-monitoring for type 2 diabetes and hypertension in sub-Saharan Africa: A systematic review, meta-analysis, and interactive network analysis
Source: PLoS Med. 2026 Aug 3;23(8):e1005189. doi: 10.1371/journal.pmed.1005189 (PMC13460740; doi:10.1371/journal.pmed.1005189)
Supplement: Supplementary file 4 [file pmed.1005189.s005.pdf]

Appendix 5. Summary of subgroup results for adherence to lifestyle recommendations

Adherence subgroup	No. of studies	Participants (N)	Pooled proportion (Random) % (95% CI)	Heterogeneity (I^2) %	P-value for heterogeneity
**Dietary recommendations (pooled adherence)**					
Mean age in years					
Age <50 years	03	1,392	46 (43–49)	0	0.077
Age ≥50 years	33	12,501	44 (37–51)	98	< 0.001
Percentage of females					
Females <50%	23	9,073	43 (34–52)	99	< 0.001
Females ≥50%	18	6,207	45 (37–54)	98	< 0.001
Rural vs urban residence					
Urban residence	20	6,670	47 (38–56)	98	< 0.001
Rural residence	02	810	35 (22–51)	95	< 0.001
Mixed residence	26	10,704	42 (35–49)	98	< 0.001
Geographical region					
West Africa	05	1,361	36 (19–59)	98	< 0.001
East Africa	41	16,028	45 (39–50)	98	< 0.001
Central/Southern Africa	02	795	43 (20–69)	98	< 0.001
Literature timepoint					
Year ≤2014	NA	NA	NA	NA	NA
Year ≥2015	47	17,898	43 (38–49)	98	< 0.001
**Physical activity recommendations (pooled adherence)**					
Mean age in years					
Age <50 years	11	4,404	40 (30–52)	98	< 0.001
Age ≥50 years	41	13,499	42 (37–48)	97	< 0.001
Percentage of females					
Females <50%	27	10,758	43 (36–50)	98	< 0.001
Females ≥50%	31	8,998	41 (34–48)	97	< 0.001
Rural vs urban residence					
Urban residence	24	6,962	38 (31–47)	98	< 0.001
Rural residence	03	884	42 (33–51)	83	< 0.001
Mixed residence	38	14,592	44 (37–50)	98	< 0.001

Adherence subgroup	No. of studies	Participants (N)	Pooled proportion (Random) % (95% CI)	Heterogeneity (I^2) %	P-value for heterogeneity
Geographical region					
West Africa	06	1,196	53 (37–69)	96	< 0.001
East Africa	51	18,423	42 (37–47)	98	< 0.001
Central/Southern Africa	08	2,819	30 (19–45)	98	< 0.001
Literature timepoint					
Year ≤2014	02	405	56 (38–73)	90	< 0.001
Year ≥2015	62	21,722	41 (36–46)	98	< 0.001
**Alcohol recommendations (pooled adherence)**					
Mean age in years					
Age <50 years	12	4,958	77 (67–85)	98	< 0.001
Age ≥50 years	47	17,782	85 (81–88)	97	< 0.001
Percentage of females					
Females <50%	23	8,947	81 (75–86)	98	< 0.001
Females ≥50%	47	17,126	87 (83–90)	97	< 0.001
Rural vs urban residence					
Urban residence	31	9,793	87 (84–90)	96	< 0.001
Rural residence	04	426	94 (86–98)	80	0.001
Mixed residence	37	16,327	82 (77–86)	98	< 0.001
Geographical region					
West Africa	11	5,617	88 (82–92)	96	< 0.001
East Africa	55	18,742	84 (81–87)	97	< 0.001
Central/Southern Africa	06	2,187	89 (70–97)	99	< 0.001
Literature timepoint					
Year ≤2014	04	722	63 (47–77)	93	< 0.001
Year ≥2015	67	25,726	86 (83–89)	98	< 0.001
**Smoking recommendations (pooled adherence)**					
Mean age in years					
Age <50 years	12	4,882	91 (86–94)	96	< 0.001
Age ≥50 years	59	23,487	96 (94–97)	96	< 0.001
Percentage of females					
Females <50%	26	10,294	92 (90–94)	92	< 0.001

Adherence subgroup	No. of studies	Participants (N)	Pooled proportion (Random) % (95% CI)	Heterogeneity (I^2) %	P-value for heterogeneity
Females ≥50%	55	21,280	96 (95–97)	96	< 0.001
Rural vs urban residence					
Urban residence	33	10,248	96 (95–97)	92	< 0.001
Rural residence	05	846	92 (84–96)	87	< 0.001
Mixed residence	44	20,891	94 (92–95)	97	< 0.001
Geographical region					
West Africa	16	8,770	97 (94–98)	96	< 0.001
East Africa	58	20,599	95 (93–96)	95	< 0.001
Central/Southern Africa	08	2,616	89 (80–94)	97	< 0.001
Literature timepoint					
Year ≤2014	08	1,600	95 (88–98)	94	< 0.001
Year ≥2015	74	30,385	95 (93–96)	96	< 0.001

Subgroup analyses of pooled adherence to dietary, physical activity, alcohol and smoking recommendations. Results are presented as pooled proportions (random-effects generalized linear mixed models, GLMM) with 95% confidence intervals (CI). Heterogeneity was assessed using the I^2 statistic, with corresponding p-values for heterogeneity tests. Geographic region was established using the WHO AFRO classification.
