# Supplementary material for: Adherence to medications, lifestyle advice, and self-monitoring for type 2 diabetes and hypertension in sub-Saharan Africa: A systematic review, meta-analysis, and interactive network analysis
Source: PLoS Med. 2026 Aug 3;23(8):e1005189. doi: 10.1371/journal.pmed.1005189 (PMC13460740; doi:10.1371/journal.pmed.1005189)
Supplement: Supplementary file 5 [file pmed.1005189.s006.pdf]

Appendix 6. Summary of subgroup results for adherence to self-monitoring

Adherence subgroup	No. of studies	Participants (N)	Pooled proportion (Random) % (95% CI)	Heterogeneity (I^2) %	P-value for heterogeneity
**Blood glucose self-monitoring (pooled adherence)**					
Mean age in years					
Age <50 years	06	2,268	15 (4–43)	99	< 0.001
Age ≥50 years	27	8,786	16 (9–24)	99	< 0.001
Percentage of females					
Females <50%	15	5,737	26 (17–39)	99	< 0.001
Females ≥50%	23	6,714	13 (7–23)	99	< 0.001
Rural vs urban residence					
Urban residence	16	4,321	21 (13–32)	98	< 0.001
Rural residence	NA	NA	NA	NA	NA
Mixed residence	22	7,776	15 (8–27)	99	< 0.001
Geographical region					
West Africa	06	1,245	15 (5–40)	99	< 0.001
East Africa	29	10,048	23 (16–33)	99	< 0.001
Central/Southern Africa	04	1,215	3 (1–14)	96	< 0.001
Literature timepoint					
Year ≤2014	05	1,414	16 (3–50)	99	< 0.001
Year ≥2015	34	11,094	19 (12–27)	99	< 0.001
**Blood pressure self-monitoring (pooled adherence)**					
Mean age in years					
Age <50 years	03	843	22 (4–64)	99	< 0.001
Age ≥50 years	03	908	32 (25–41)	85	< 0.001
Percentage of females					
Females <50%	04	1,236	18 (8–35)	97	< 0.001
Females ≥50%	03	837	42 (20–68)	98	< 0.001
Rural vs urban residence					
Urban residence	05	1,218	42 (27–58)	96	< 0.001
Rural residence	NA	NA	NA	NA	NA
Mixed residence	03	1,136	13 (6–24)	94	< 0.001

Adherence subgroup	No. of studies	Participants (N)	Pooled proportion (Random) % (95% CI)	Heterogeneity (I^2) %	P-value for heterogeneity
Geographical region					
West Africa	03	566	53 (36–70)	93	< 0.001
East Africa	05	1,788	17 (10–28)	96	< 0.001
Central/Southern Africa	NA	NA	NA	NA	NA
Literature timepoint					
Year ≤2014	NA	NA	NA	NA	NA
Year ≥2015	08	2,354	28 (16–45)	98	< 0.001

Subgroup analyses of pooled adherence to blood glucose and blood pressure self-monitoring. Results are presented as pooled proportions (random-effects generalized linear mixed models, GLMM) with 95% confidence intervals (CI). Heterogeneity was assessed using the I^2 statistic, with corresponding p-values for heterogeneity tests. Geographic region was established using the WHO AFRO classification.
