# Supplementary material for: Adherence to medications, lifestyle advice, and self-monitoring for type 2 diabetes and hypertension in sub-Saharan Africa: A systematic review, meta-analysis, and interactive network analysis
Source: PLoS Med. 2026 Aug 3;23(8):e1005189. doi: 10.1371/journal.pmed.1005189 (PMC13460740; doi:10.1371/journal.pmed.1005189)
Supplement: Supplementary file 6 [file pmed.1005189.s007.pdf]

Appendix 7. Summary of subgroup results for overall self-care adherence

Adherence subgroup	No. of studies	Participants (N)	Pooled proportion (Random) % (95% CI)	Heterogeneity (I^2) %	P-value for heterogeneity
**Overall diabetes self-care (pooled adherence)**					
Mean age in years					
Age <50 years	02	659	20 (3–69)	99	< 0.001
Age ≥50 years	10	3,027	42 (32–52)	96	< 0.001
Percentage of females					
Females <50%	11	4,334	51 (47–55)	85	< 0.001
Females ≥50%	10	3,287	26 (16–40)	98	< 0.001
Rural vs urban residence					
Urban residence	12	3,344	37 (24–53)	99	< 0.001
Rural residence	04	1,457	36 (26–48)	94	< 0.001
Mixed residence	15	6,549	37 (27–49)	99	< 0.001
Geographical region					
West Africa	NA	NA	NA	NA	NA
East Africa	26	9,790	43 (37–49)	97	< 0.001
Central/Southern Africa	05	1,560	14 (4–37)	98	< 0.001
Literature timepoint					
Year ≤2014	02	291	12 (1–64)	96	< 0.001
Year ≥2015	29	11,059	39 (32–47)	98	< 0.001
**Overall hypertension self-care (pooled adherence)**					
Mean age in years					
Age <50 years	05	1,299	40 (22–61)	98	< 0.001
Age ≥50 years	09	3,047	29 (18–42)	98	< 0.001
Percentage of females					
Females <50%	11	4,059	39 (32–45)	94	< 0.001
Females ≥50%	09	2,485	30 (17–47)	98	< 0.001
Rural vs urban residence					
Urban residence	18	5,488	36 (26–46)	98	< 0.001
Rural residence	03	781	35 (28–44)	1	0.197
Mixed residence	09	3,852	35 (26–45)	97	< 0.001
Geographical region					

Adherence subgroup	No. of studies	Participants (N)	Pooled proportion (Random) % (95% CI)	Heterogeneity (I^2) %	P-value for heterogeneity
West Africa	03	1,009	39 (13–72)	98	< 0.001
East Africa	25	8,642	37 (31–43)	97	< 0.001
Central/Southern Africa	02	470	17 (3–55)	97	< 0.001
Literature timepoint					
Year ≤2014	NA	NA	NA	NA	NA
Year ≥2015	30	10,121	35 (29–42)	98	< 0.001

Subgroup analyses of pooled overall self-care adherence in diabetes and hypertension. Results are presented as pooled proportions (random-effects generalized linear mixed models, GLMM) with 95% confidence intervals (CI). Heterogeneity was assessed using the I^2 statistic, with corresponding p-values for heterogeneity tests. Geographic region was established using the WHO AFRO classification.
