# Supplementary material for: Adherence to medications, lifestyle advice, and self-monitoring for type 2 diabetes and hypertension in sub-Saharan Africa: A systematic review, meta-analysis, and interactive network analysis
Source: PLoS Med. 2026 Aug 3;23(8):e1005189. doi: 10.1371/journal.pmed.1005189 (PMC13460740; doi:10.1371/journal.pmed.1005189)
Supplement: Supplementary file 8 [file pmed.1005189.s009.pdf]

Appendix 9. Sensitivity analysis: summary of pooled adherence among low risk-of-bias studies

Adherence Domain	Number of studies	Total participants	Pooled adherence (Random) % (95% CI)	I² (%)	P-value
Anti-diabetic medications	43	12,445	64 (55–73)	99	< 0.001
Anti-hypertensive medications	46	17,553	49 (40–58)	99	< 0.001
Dietary recommendations	40	15,106	47 (41–53)	98	< 0.001
Physical activity recommendations	50	17,556	42 (36–47)	98	< 0.001
Alcohol recommendations	46	16,337	85 (81–89)	98	< 0.001
Smoking recommendations	51	20,937	94 (93–96)	96	< 0.001
Blood glucose self-monitoring	21	7,239	19 (11–29)	99	< 0.001
Blood pressure self-monitoring	06	2,069	20 (12–32)	97	< 0.001
Overall diabetes self-care	22	8,707	40 (32–48)	98	< 0.001
Overall hypertension self-care	22	7,415	35 (28–41)	97	< 0.001

Sensitivity analysis restricted to studies judged at low risk of bias (Newcastle-Ottawa Scale low rating for observational studies; RoB 2 low risk for randomised trials; Appendix 13). Results are pooled proportions from random-effects logistic-normal generalized linear mixed models (GLMM) with 95% confidence intervals (CI). Heterogeneity was assessed using the I^2 statistic with the corresponding p-value (Q test). Estimates use the logit transformation.
