# Supplementary material for: Adherence to medications, lifestyle advice, and self-monitoring for type 2 diabetes and hypertension in sub-Saharan Africa: A systematic review, meta-analysis, and interactive network analysis
Source: PLoS Med. 2026 Aug 3;23(8):e1005189. doi: 10.1371/journal.pmed.1005189 (PMC13460740; doi:10.1371/journal.pmed.1005189)
Supplement: Supplementary file 9 [file pmed.1005189.s010.pdf]

Appendix 10a. Anti-diabetic medications — sensitivity analysis (low risk-of-bias studies)

Random-effects meta-analysis | k = 43 studies | Total N = 12,445 | Events = 7,478

Events / N	Study	Proportion [95% CI]
82 / 469	Kamuhabwa 2014	17% [14%, 21%]
30 / 155	Bizu 2016	19% [13%, 26%]
93 / 403	Sendekie 2022	23% [19%, 28%]
66 / 273	Mariye 2019	24% [19%, 30%]
22 / 89	Ikolaba 2023	25% [16%, 35%]
63 / 188	Kretchy 2020	34% [27%, 41%]
142 / 422	Wondm 2024	34% [29%, 38%]
136 / 400	Tefera 2020	34% [29%, 39%]
77 / 200	Bruce 2015	38% [32%, 46%]
158 / 404	Owolabi 2024	39% [34%, 44%]
131 / 289	Waari 2018	45% [39%, 51%]
141 / 305	Dimore 2023	46% [41%, 52%]
93 / 200	Murwanashyaka 2022	46% [39%, 54%]
86 / 176	Adisa 2013	49% [41%, 56%]
193 / 376	Lalam 2026	51% [46%, 56%]
103 / 188	Aje 2024	55% [47%, 62%]
80 / 146	Ali 2017	55% [46%, 63%]
169 / 305	Hadush 2024	55% [50%, 61%]
108 / 182	Tewabe 2018	59% [52%, 67%]
252 / 411	Zemen 2025	61% [56%, 66%]
186 / 300	Ibrahim 2021	62% [56%, 68%]
124 / 200	Adisa 2017	62% [55%, 69%]
157 / 248	Doya 2024	63% [57%, 69%]
148 / 228	Fekadu 2019	65% [58%, 71%]
265 / 396	Mutyambizi 2020	67% [62%, 72%]
137 / 196	Adegbola 2016	70% [63%, 76%]
562 / 794	Simegn 2023	71% [67%, 74%]
258 / 359	Tusa 2020	72% [67%, 76%]
105 / 145	Omotosho 2024	72% [64%, 80%]
268 / 357	Demoz 2020	75% [70%, 79%]
302 / 402	Zwane 2023	75% [71%, 79%]
247 / 327	AT Kassaw 2025	76% [71%, 80%]
202 / 267	Teklay 2013	76% [70%, 81%]
261 / 332	Edmealem 2020	79% [74%, 83%]
305 / 371	Mekonnen 2021	82% [78%, 86%]
289 / 348	Enikuomehin 2021	83% [79%, 87%]
251 / 300	Berhe 2017	84% [79%, 88%]
163 / 194	Sorato 2016	84% [78%, 89%]
279 / 330	Afaya 2020	85% [80%, 88%]
125 / 140	Bofu 2024	89% [83%, 94%]
311 / 320	Berhe 2012	97% [95%, 99%]
91 / 93	Gemeda 2022	98% [92%, 100%]
217 / 217	Degefa 2020	100% [98%, 100%]
	**Pooled**	**64% [55%, 73%] I² = 99% (k = 43)**

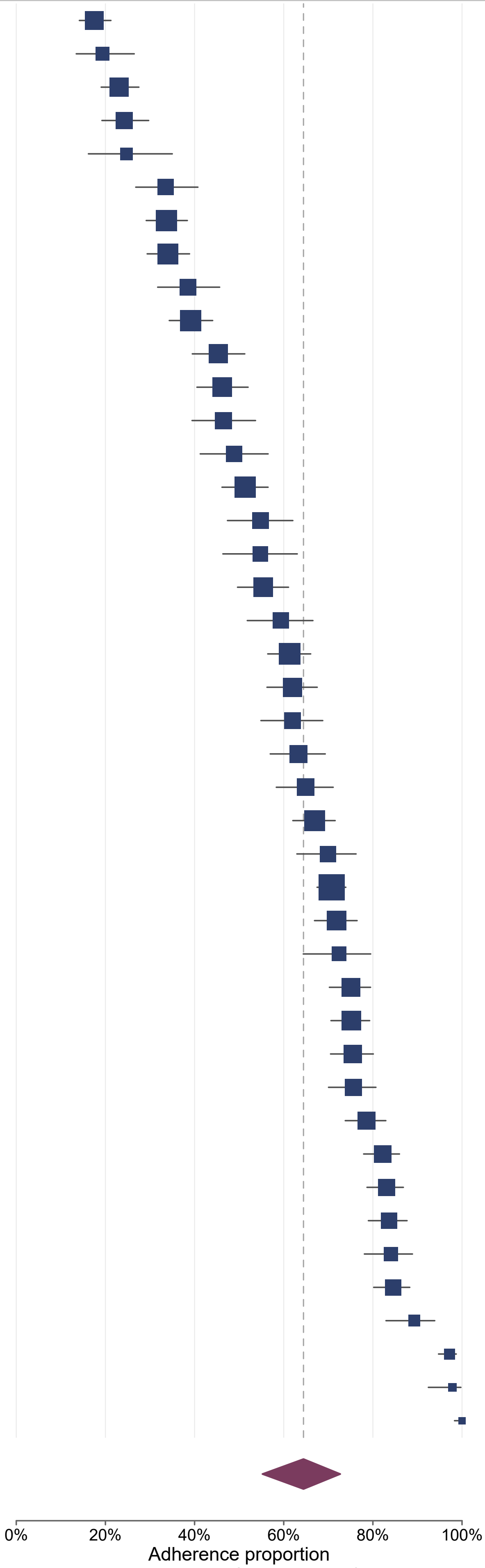

Squares: study estimates (size ∝ inverse SE). Horizontal lines: 95% CI (Clopper–Pearson). Diamond: pooled estimate (GLMM, logit transformation).

Appendix 10b. Anti-hypertensive medications — sensitivity analysis (low risk-of-bias studies)

Random-effects meta-analysis | k = 46 studies | Total N = 17,553 | Events = 8,751

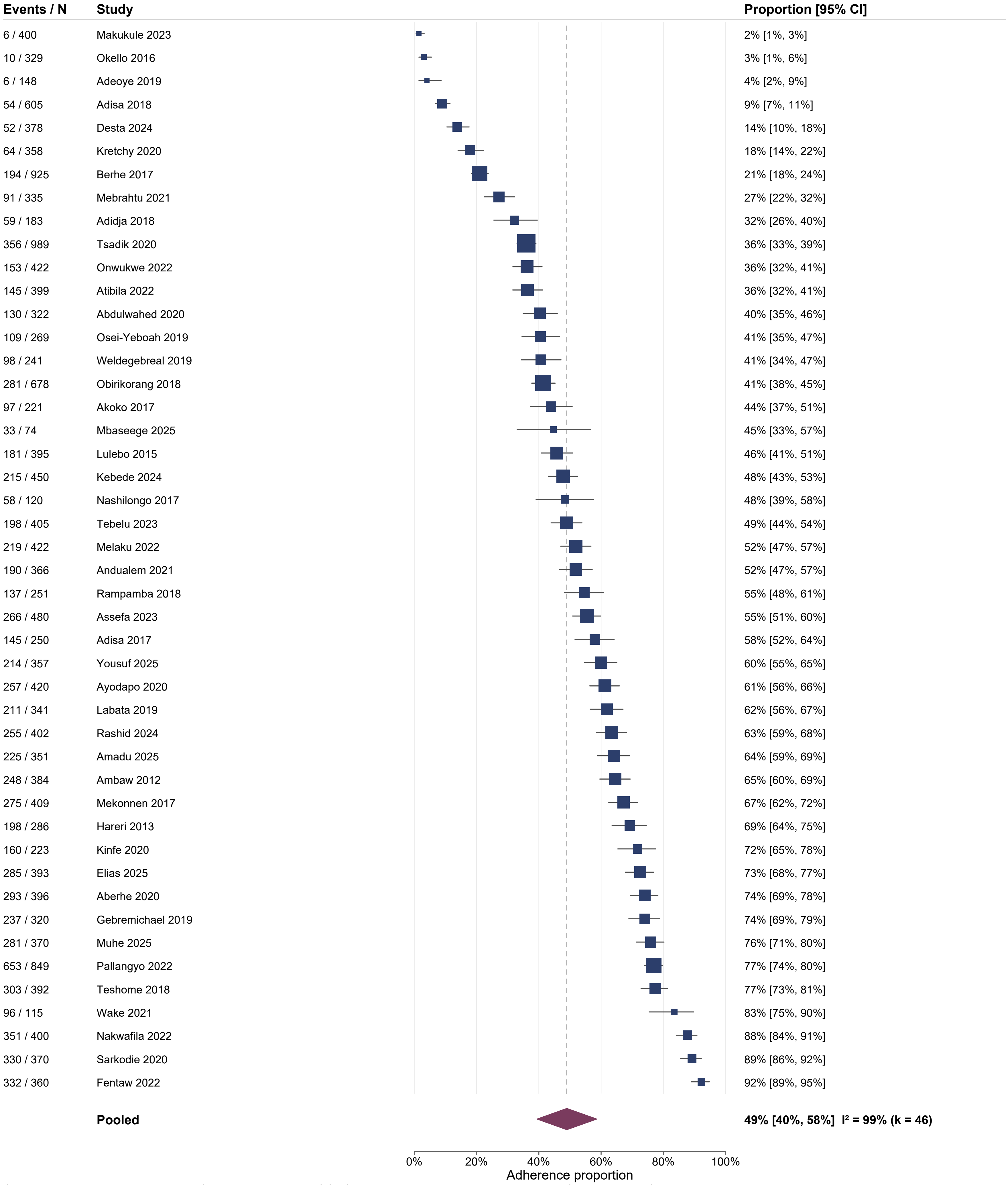

Squares: study estimates (size ∝ inverse SE). Horizontal lines: 95% CI (Clopper–Pearson). Diamond: pooled estimate (GLMM, logit transformation).

Appendix 10c. Dietary recommendations — sensitivity analysis (low risk-of-bias studies)

Random-effects meta-analysis | k = 40 studies | Total N = 15,106 | Events = 7,108

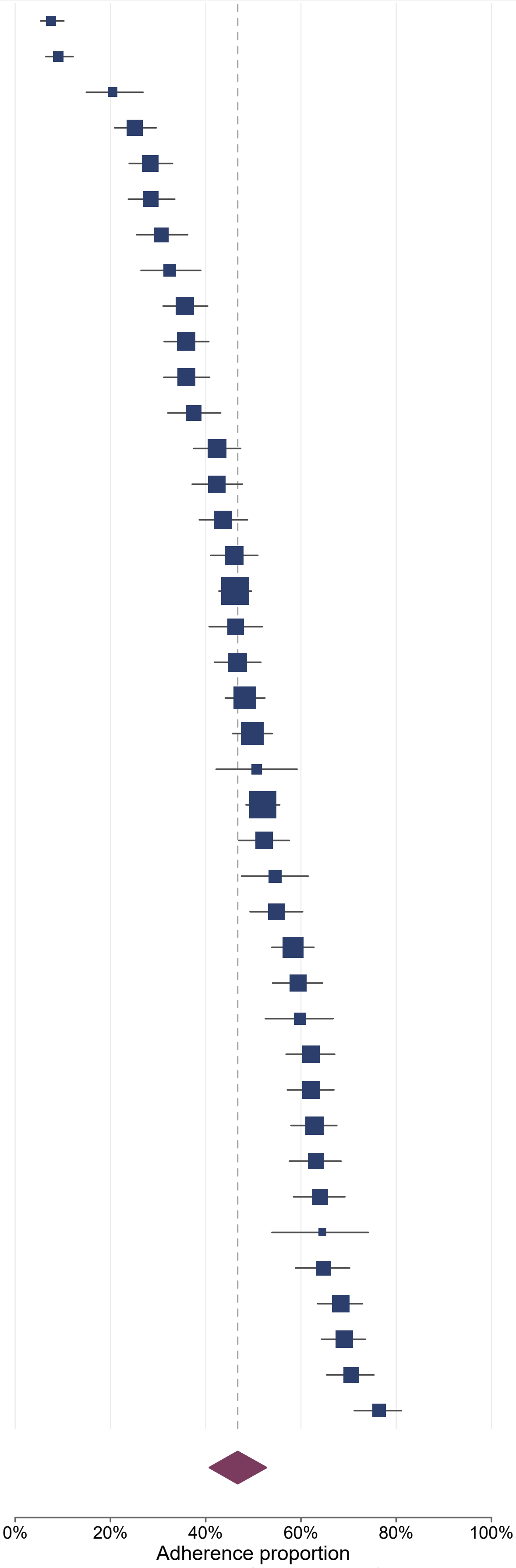

Events / N	Study	Proportion [95% CI]
36 / 480	Assefa 2023	8% [5%, 10%]
38 / 422	Melaku 2022	9% [6%, 12%]
39 / 191	Boshe 2021	20% [15%, 27%]
100 / 399	Owolabi 2024	25% [21%, 30%]
113 / 399	Abera 2024	28% [24%, 33%]
99 / 348	Enikuomehin 2021	28% [24%, 34%]
92 / 300	Berhe 2017	31% [25%, 36%]
74 / 228	Fekadu 2019	32% [26%, 39%]
150 / 421	Tirfessa 2023	36% [31%, 40%]
151 / 421	Abose 2024	36% [31%, 41%]
144 / 401	Demilew 2018	36% [31%, 41%]
115 / 307	Adem 2020	37% [32%, 43%]
172 / 406	Saliya 2024	42% [38%, 47%]
150 / 354	Jemal 2022	42% [37%, 48%]
169 / 387	Bekele 2024	44% [39%, 49%]
189 / 411	Zemen 2025	46% [41%, 51%]
388 / 840	Zeleke 2025	46% [43%, 50%]
149 / 322	Erkocho 2022	46% [41%, 52%]
197 / 422	Belay 2021	47% [42%, 52%]
278 / 576	Mekonnen 2021	48% [44%, 52%]
284 / 570	MolalignTakele 2021	50% [46%, 54%]
72 / 142	Wilson 2024	51% [42%, 59%]
413 / 794	Simegn 2023	52% [48%, 56%]
185 / 354	Wolderufael 2021	52% [47%, 58%]
113 / 207	Degefa 2020	55% [48%, 62%]
180 / 328	Zewdie 2022	55% [49%, 60%]
290 / 497	Belew 2025	58% [54%, 63%]
209 / 352	Worsa 2021	59% [54%, 65%]
116 / 194	Sorato 2016	60% [53%, 67%]
226 / 364	Muhe 2025	62% [57%, 67%]
246 / 396	Mutyambizi 2020	62% [57%, 67%]
255 / 406	Atinafu 2025	63% [58%, 68%]
202 / 320	Gebremichael 2019	63% [58%, 68%]
206 / 322	ZelekeNegera 2020	64% [58%, 69%]
60 / 93	Gemeda 2022	65% [54%, 74%]
185 / 286	Hareri 2013	65% [59%, 70%]
272 / 398	Gebermariam 2020	68% [64%, 73%]
279 / 404	Tibebu 2017	69% [64%, 74%]
242 / 343	Atosona 2024	71% [65%, 75%]
230 / 301	Andualem 2020	76% [71%, 81%]
	**Pooled**	**47% [41%, 53%] I^2 = 98% (k = 40)**

Squares: study estimates (size ∝ inverse SE). Horizontal lines: 95% CI (Clopper–Pearson). Diamond: pooled estimate (GLMM, logit transformation).

Appendix 10d. Physical activity recommendations — sensitivity analysis (low risk-of-bias studies)

Random-effects meta-analysis | k = 50 studies | Total N = 17,556 | Events = 7,543

Events / N	Study	Proportion [95% CI]
36 / 396	Mutyambizi 2020	9% [6%, 12%]
44 / 406	Saliya 2024	11% [8%, 14%]
25 / 191	Boshe 2021	13% [9%, 19%]
54 / 402	Zwane 2023	13% [10%, 17%]
33 / 205	Buda 2017	16% [11%, 22%]
83 / 480	Assefa 2023	17% [14%, 21%]
54 / 305	Dimore 2023	18% [14%, 22%]
31 / 140	Bofu 2024	22% [16%, 30%]
99 / 400	Makukule 2023	25% [21%, 29%]
105 / 422	Melaku 2022	25% [21%, 29%]
125 / 422	Abdisa 2022	30% [25%, 34%]
121 / 391	Hussen 2020	31% [26%, 36%]
110 / 350	Tozivepi 2021	31% [27%, 37%]
127 / 404	Tibebu 2017	31% [27%, 36%]
128 / 399	Owolabi 2024	32% [28%, 37%]
30 / 93	Gemeda 2022	32% [23%, 43%]
127 / 378	Desta 2024	34% [29%, 39%]
115 / 322	ZelekeNegera 2020	36% [30%, 41%]
81 / 207	Degefa 2020	39% [32%, 46%]
129 / 328	Zewdie 2022	39% [34%, 45%]
120 / 301	Andualem 2020	40% [34%, 46%]
340 / 840	Zeleke 2025	40% [37%, 44%]
135 / 330	Philbert 2023	41% [36%, 46%]
168 / 405	Tebelu 2023	41% [37%, 46%]
125 / 286	Hareri 2013	44% [38%, 50%]
176 / 398	Geremew 2023	44% [39%, 49%]
174 / 392	Teshome 2018	44% [39%, 49%]
153 / 341	Labata 2019	45% [40%, 50%]
124 / 276	Niriayo 2019	45% [39%, 51%]
77 / 170	Shiriyedeve 2019	45% [38%, 53%]
188 / 411	Zemen 2025	46% [41%, 51%]
158 / 320	Gebremichael 2019	49% [44%, 55%]
284 / 570	MolalignTakele 2021	50% [46%, 54%]
37 / 74	Mbaseege 2025	50% [38%, 62%]
98 / 194	Sorato 2016	51% [43%, 58%]
196 / 387	Bekele 2024	51% [46%, 56%]
181 / 354	Wolderufael 2021	51% [46%, 56%]
203 / 392	Gelaw 2021	52% [47%, 57%]
424 / 794	Simegn 2023	53% [50%, 57%]
300 / 497	Belew 2025	60% [56%, 65%]
262 / 422	Debalke 2022	62% [57%, 67%]
299 / 450	Kebede 2024	66% [62%, 71%]
222 / 332	Edmealem 2020	67% [62%, 72%]
214 / 320	Ayele 2019	67% [61%, 72%]
233 / 348	Enikuomehin 2021	67% [62%, 72%]
82 / 119	Oyewole 2014	69% [60%, 77%]
104 / 150	Osei-Yeboah 2019	69% [61%, 77%]
222 / 300	Berhe 2017	74% [69%, 79%]
247 / 322	Abdulwahed 2020	77% [72%, 81%]
340 / 420	Belay 2023	81% [77%, 85%]
	**Pooled**	**42% [36%, 47%] I² = 98% (k = 50)**

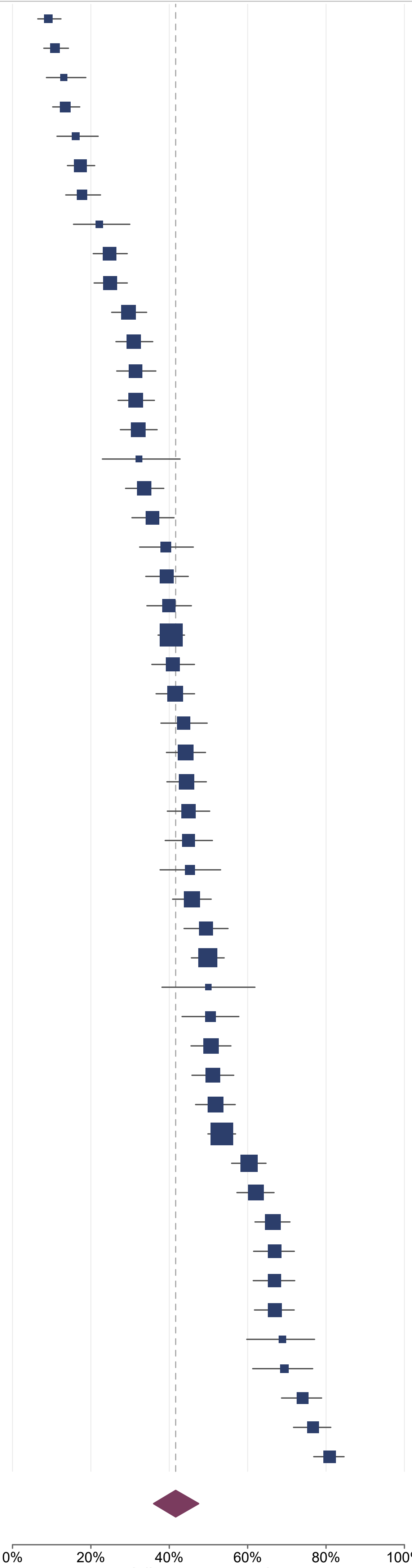

Squares: study estimates (size ∝ inverse SE). Horizontal lines: 95% CI (Clopper–Pearson). Diamond: pooled estimate (GLMM, logit transformation).

Appendix 10e. Alcohol recommendations — sensitivity analysis (low risk-of-bias studies)

Random-effects meta-analysis | k = 46 studies | Total N = 16,337 | Events = 13,060

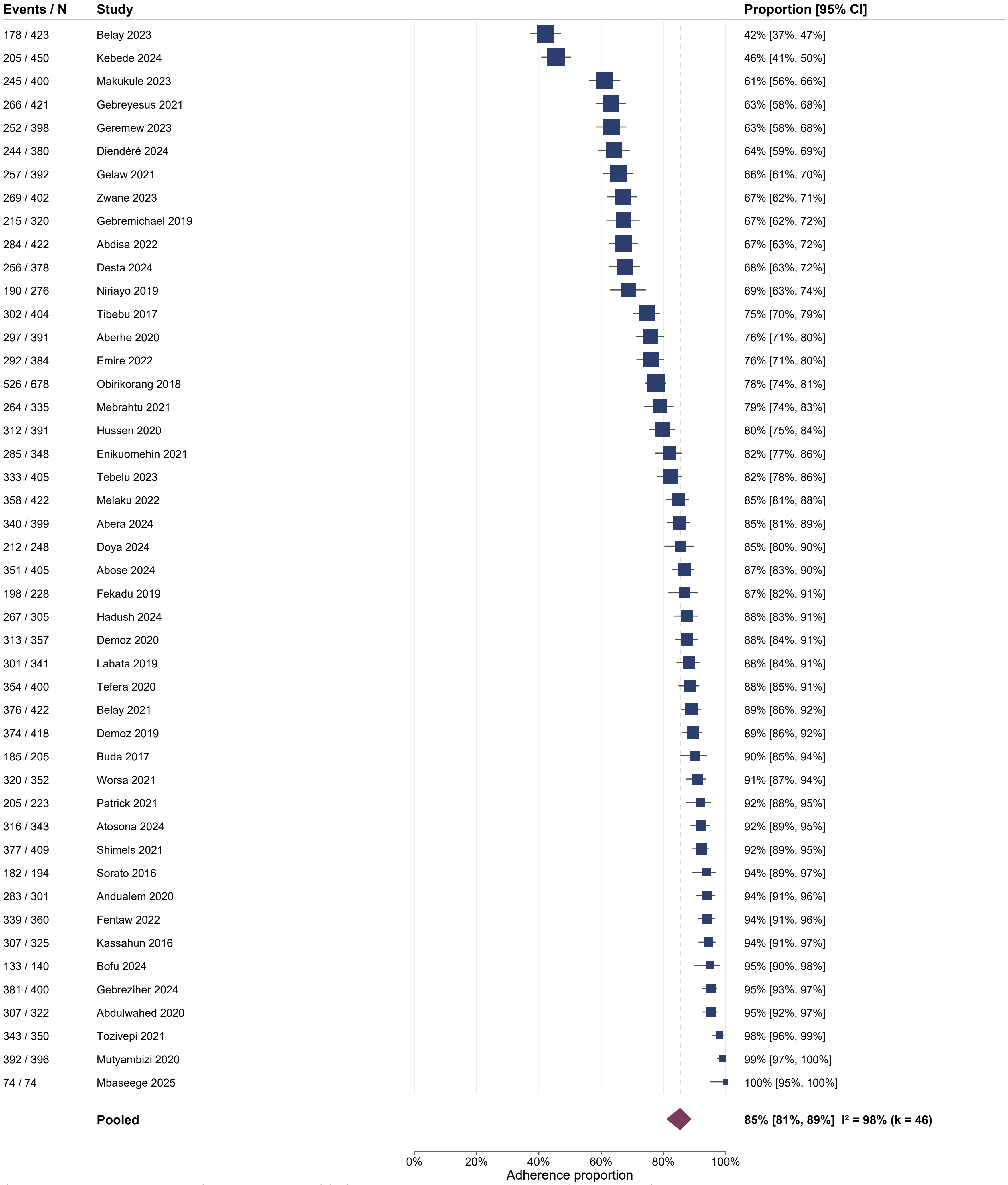

Squares: study estimates (size ∝ inverse SE). Horizontal lines: 95% CI (Clopper–Pearson). Diamond: pooled estimate (GLMM, logit transformation).

Appendix 10f. Smoking recommendations — sensitivity analysis (low risk-of-bias studies)

Random-effects meta-analysis | k = 51 studies | Total N = 20,937 | Events = 19,270

Events / N	Study	Proportion [95% CI]
265 / 402	Zwane 2023	66% [61%, 71%]
300 / 411	Zemen 2025	73% [68%, 77%]
320 / 391	Hussen 2020	82% [78%, 86%]
319 / 378	Desta 2024	84% [80%, 88%]
338 / 400	Makukule 2023	84% [81%, 88%]
337 / 396	Mutyambizi 2020	85% [81%, 88%]
362 / 422	Abdisa 2022	86% [82%, 89%]
357 / 416	Tibebu 2017	86% [82%, 89%]
277 / 322	ZelekeNegera 2020	86% [82%, 90%]
333 / 384	Emire 2022	87% [83%, 90%]
331 / 380	Diendéré 2024	87% [83%, 90%]
198 / 226	Jackson 2021	88% [83%, 92%]
350 / 399	Abera 2024	88% [84%, 91%]
350 / 398	Geremew 2023	88% [84%, 91%]
740 / 840	Zeleke 2025	88% [86%, 90%]
270 / 301	Andualem 2020	90% [86%, 93%]
248 / 276	Niriayo 2019	90% [86%, 93%]
67 / 74	Mbaseege 2025	91% [81%, 96%]
369 / 405	Tebelu 2023	91% [88%, 94%]
192 / 210	Buda 2017	91% [87%, 95%]
623 / 678	Obirikorang 2018	92% [90%, 94%]
322 / 348	Enikuomehin 2021	93% [89%, 95%]
900 / 968	Berhe 2017	93% [91%, 95%]
213 / 228	Fekadu 2019	93% [89%, 96%]
319 / 341	Labata 2019	94% [90%, 96%]
395 / 422	Melaku 2022	94% [91%, 96%]
287 / 305	Hadush 2024	94% [91%, 96%]
2086 / 2198	Cavagna 2021	95% [94%, 96%]
429 / 450	Kebede 2024	95% [93%, 97%]
382 / 398	Gebermariam 2020	96% [94%, 98%]
406 / 423	Belay 2023	96% [94%, 98%]
346 / 360	Fentaw 2022	96% [94%, 98%]
330 / 343	Atosona 2024	96% [94%, 98%]
323 / 335	Mebrahtu 2021	96% [94%, 98%]
386 / 400	Gebreziher 2024	96% [94%, 98%]
464 / 480	Assefa 2023	97% [95%, 98%]
339 / 350	Tozivepi 2021	97% [94%, 98%]
300 / 309	Kassahun 2016	97% [95%, 99%]
241 / 248	Doya 2024	97% [94%, 99%]
313 / 322	Abdulwahed 2020	97% [95%, 99%]
389 / 399	Gelaw 2021	97% [95%, 99%]
395 / 405	Abose 2024	98% [96%, 99%]
218 / 223	Patrick 2021	98% [95%, 99%]
414 / 422	Belay 2021	98% [96%, 99%]
314 / 320	Berhe 2012	98% [96%, 99%]
385 / 391	Aberhe 2020	98% [97%, 99%]
138 / 140	Bofu 2024	99% [95%, 100%]
387 / 392	Teshome 2018	99% [97%, 100%]
354 / 357	Demoz 2020	99% [98%, 100%]
355 / 357	Demoz 2019	99% [98%, 100%]
194 / 194	Sorato 2016	100% [98%, 100%]
	**Pooled**	**94% [93%, 96%] I² = 96% (k = 51)**

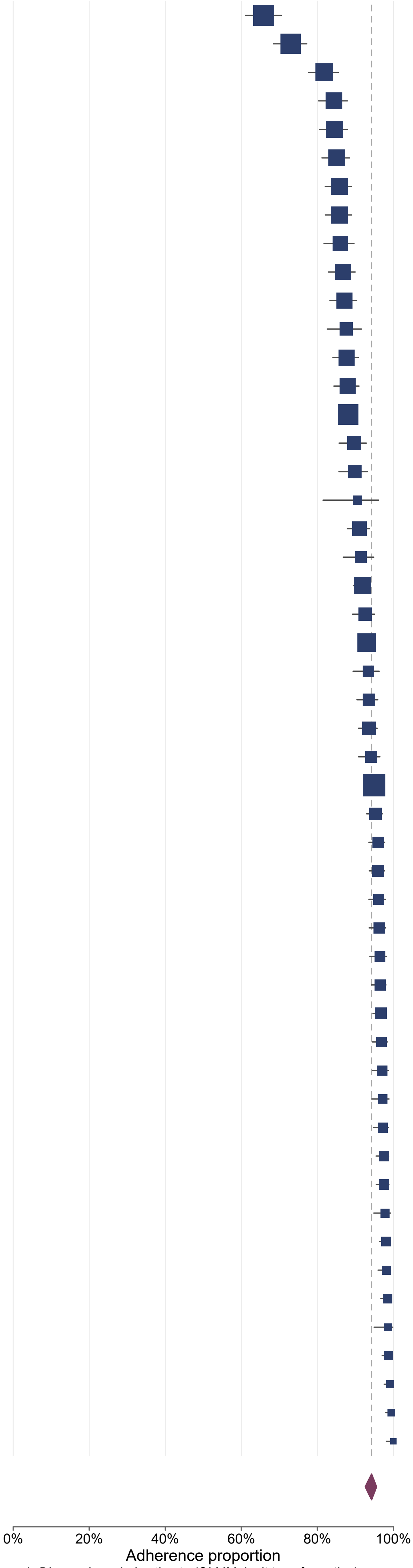

Squares: study estimates (size ∝ inverse SE). Horizontal lines: 95% CI (Clopper–Pearson). Diamond: pooled estimate (GLMM, logit transformation).

Appendix 10g. Blood glucose self-monitoring — sensitivity analysis (low risk-of-bias studies)

Random-effects meta-analysis | k = 21 studies | Total N = 7,239 | Events = 1,819

Events / N	Study	Proportion [95% CI]
8 / 402	Zwane 2023	2% [1%, 4%]
5 / 194	Sorato 2016	3% [1%, 6%]
13 / 422	Belay 2021	3% [2%, 5%]
16 / 469	Kamuhabwa 2014	3% [2%, 5%]
28 / 401	Demilew 2018	7% [5%, 10%]
24 / 320	Ayele 2019	8% [5%, 11%]
40 / 371	Mekonnen 2021	11% [8%, 14%]
19 / 140	Bofu 2024	14% [8%, 20%]
42 / 300	Berhe 2017	14% [10%, 18%]
50 / 320	Berhe 2012	16% [12%, 20%]
41 / 180	Legese 2023	23% [17%, 30%]
102 / 410	Mamo 2019	25% [21%, 29%]
156 / 600	Endale 2025	26% [23%, 30%]
61 / 217	Degefa 2020	28% [22%, 35%]
267 / 794	Simegn 2023	34% [30%, 37%]
32 / 93	Gemeda 2022	34% [25%, 45%]
84 / 226	Ewunetu 2025	37% [31%, 44%]
198 / 354	Wolderufael 2021	56% [51%, 61%]
229 / 387	Bekele 2024	59% [54%, 64%]
250 / 411	Zemen 2025	61% [56%, 66%]
154 / 228	Fekadu 2019	68% [61%, 74%]
	**Pooled**	**19% [11%, 29%] I^2 = 99% (k = 21)**

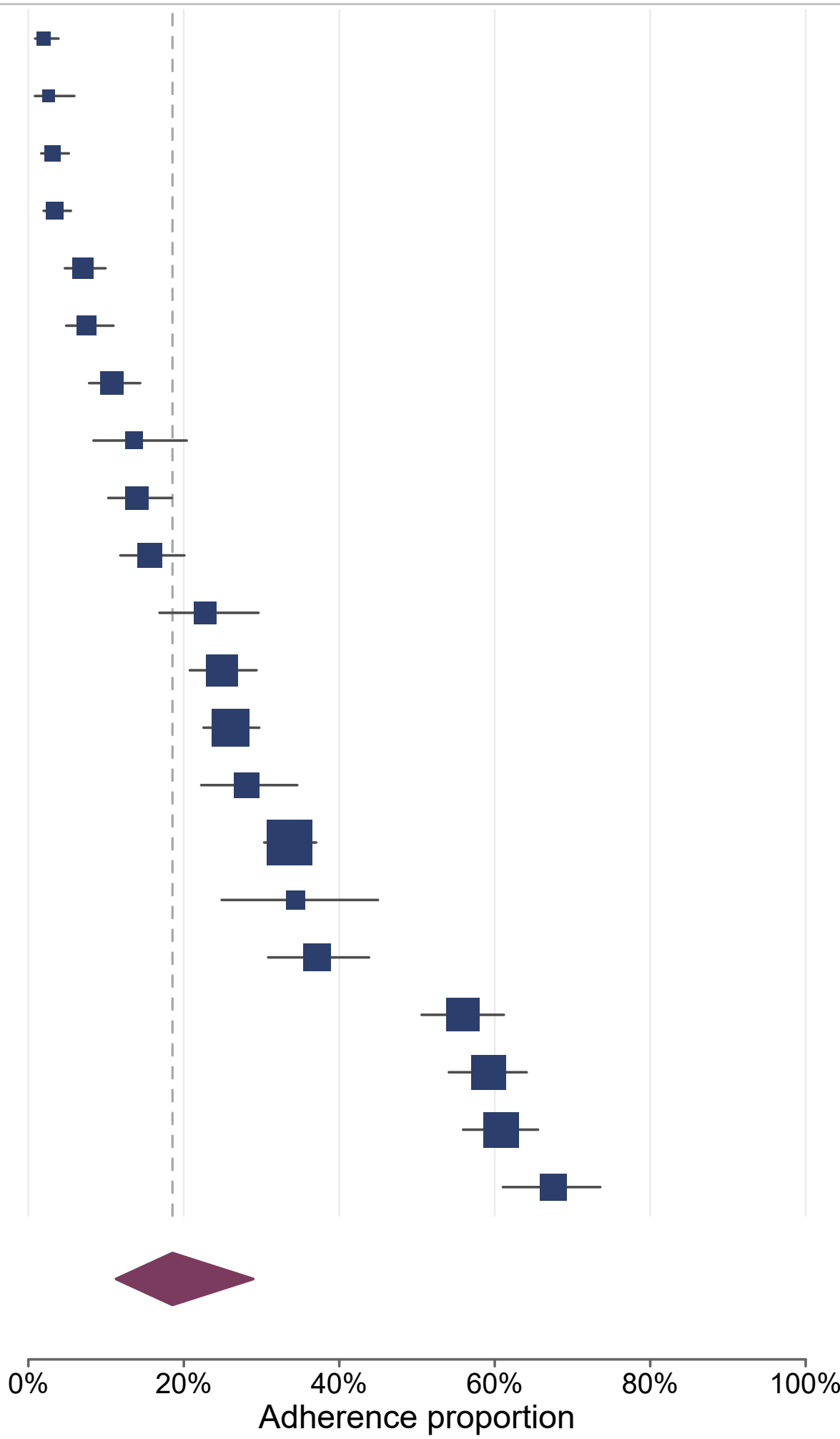

Squares: study estimates (size ∝ inverse SE). Horizontal lines: 95% CI (Clopper–Pearson). Diamond: pooled estimate (GLMM, logit transformation).

Appendix 10h. Blood pressure self-monitoring — sensitivity analysis (low risk-of-bias studies)

Random-effects meta-analysis | k = 6 studies | Total N = 2,069 | Events = 459

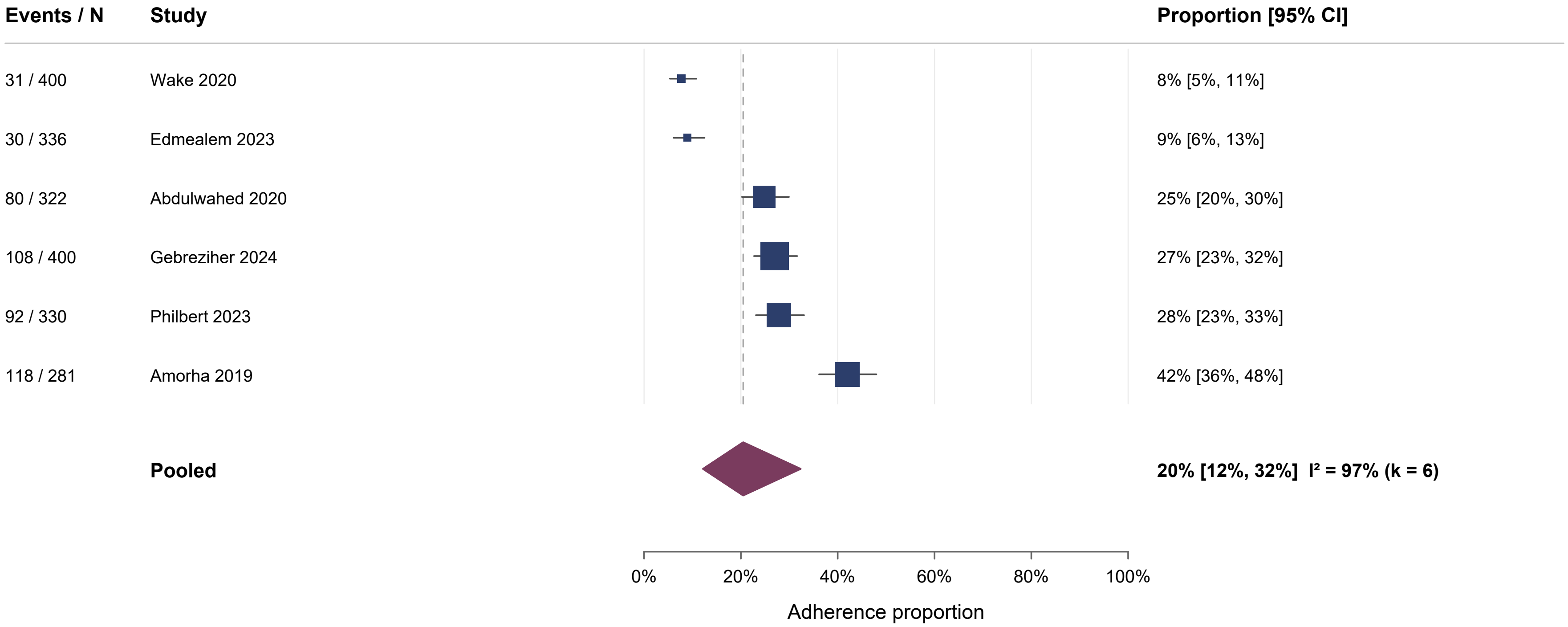

Squares: study estimates (size ∝ inverse SE). Horizontal lines: 95% CI (Clopper–Pearson). Diamond: pooled estimate (GLMM, logit transformation).

Appendix 10i. Overall diabetes self-care — sensitivity analysis (low risk-of-bias studies)

Random-effects meta-analysis | k = 22 studies | Total N = 8,707 | Events = 3,688

Events / N	Study	Proportion [95% CI]
20 / 402	Zwane 2023	5% [3%, 8%]
12 / 140	Bofu 2024	9% [5%, 14%]
51 / 422	Wondm 2024	12% [9%, 16%]
240 / 726	Kiarie 2023	33% [30%, 37%]
106 / 284	Mariye 2018	37% [32%, 43%]
122 / 320	Ayele 2019	38% [33%, 44%]
152 / 384	Emire 2022	40% [35%, 45%]
80 / 194	Sorato 2016	41% [34%, 49%]
337 / 794	Simegn 2023	42% [39%, 46%]
262 / 600	Endale 2025	44% [40%, 48%]
178 / 387	Bekele 2024	46% [41%, 51%]
266 / 570	MolalignTakele 2021	47% [43%, 51%]
99 / 207	Degefa 2020	48% [41%, 55%]
178 / 371	Jemal 2022	48% [43%, 53%]
170 / 354	Wolderufael 2021	48% [43%, 53%]
152 / 309	Kassahun 2016	49% [43%, 55%]
173 / 344	Zewdie 2022	50% [45%, 56%]
153 / 300	Berhe 2017	51% [45%, 57%]
201 / 371	Mekonnen 2021	54% [49%, 59%]
249 / 432	Eshete 2023	58% [53%, 62%]
244 / 411	Zemen 2025	59% [54%, 64%]
243 / 385	Tiruneh 2019	63% [58%, 68%]
	**Pooled**	**40% [32%, 48%] I² = 98% (k = 22)**

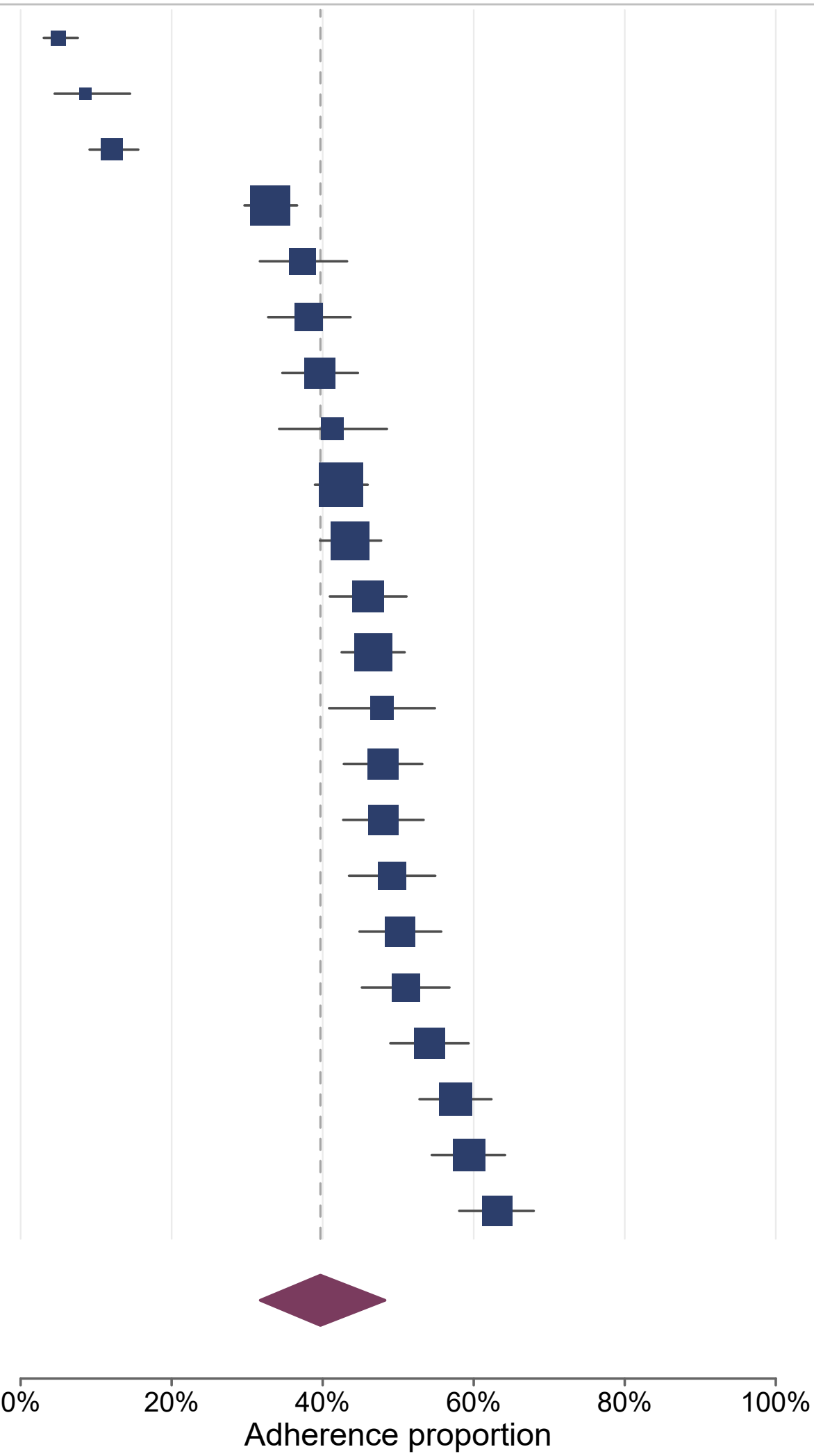

Squares: study estimates (size ∝ inverse SE). Horizontal lines: 95% CI (Clopper–Pearson). Diamond: pooled estimate (GLMM, logit transformation).

Appendix 10j. Overall hypertension self-care — sensitivity analysis (low risk-of-bias studies)

Random-effects meta-analysis | k = 22 studies | Total N = 7,415 | Events = 2,754

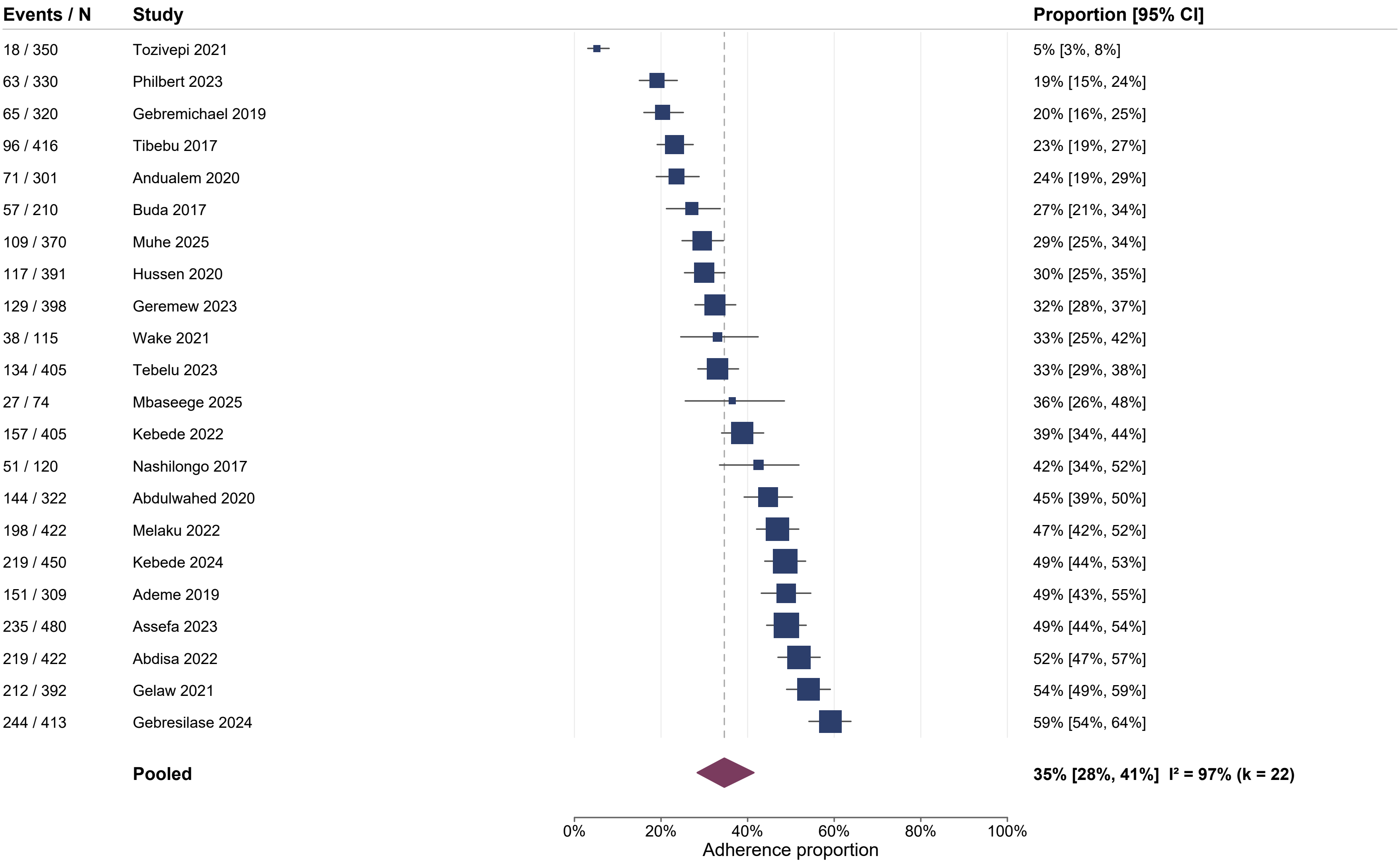

Squares: study estimates (size ∝ inverse SE). Horizontal lines: 95% CI (Clopper–Pearson). Diamond: pooled estimate (GLMM, logit transformation).
