# Supplementary material for: Adherence to medications, lifestyle advice, and self-monitoring for type 2 diabetes and hypertension in sub-Saharan Africa: A systematic review, meta-analysis, and interactive network analysis
Source: PLoS Med. 2026 Aug 3;23(8):e1005189. doi: 10.1371/journal.pmed.1005189 (PMC13460740; doi:10.1371/journal.pmed.1005189)
Supplement: Supplementary file 10 [file pmed.1005189.s011.pdf]

Appendix 11. Sensitivity analysis: summary of pooled adherence among studies using validated adherence tools

Adherence Domain	Number of studies	Total participants	Pooled adherence (Random) % (95% CI)	I^2 (%)	P-value
Anti-diabetic medications	56	14,976	60 (52–67)	99	< 0.001
Anti-hypertensive medications	54	18,635	46 (37–56)	99	< 0.001
Dietary recommendations	23	8,571	44 (34–54)	99	< 0.001
Physical activity recommendations	31	10,050	42 (36–49)	98	< 0.001
Alcohol recommendations	13	4,484	86 (76–92)	99	< 0.001
Smoking recommendations	00	0	NA	NA	NA
Blood glucose self-monitoring	10	3,457	26 (16–40)	98	< 0.001
Blood pressure self-monitoring	00	0	NA	NA	NA
Overall diabetes self-care	25	9,213	36 (28–46)	99	< 0.001
Overall hypertension self-care	26	8,812	36 (29–43)	98	< 0.001

Sensitivity analysis restricted to studies that used a validated adherence assessment tool (e.g., MMAS/Morisky, GMAS, SDSCA, H-SCALE). Results are pooled proportions from random-effects logistic-normal generalized linear mixed models (GLMM) with 95% confidence intervals (CI). Heterogeneity was assessed using the I^2 statistic with the corresponding p-value (Q test). Estimates use the logit transformation. NA indicates too few studies ($k < 2$) for pooling.
