# Supplementary material for: Adherence to medications, lifestyle advice, and self-monitoring for type 2 diabetes and hypertension in sub-Saharan Africa: A systematic review, meta-analysis, and interactive network analysis
Source: PLoS Med. 2026 Aug 3;23(8):e1005189. doi: 10.1371/journal.pmed.1005189 (PMC13460740; doi:10.1371/journal.pmed.1005189)
Supplement: Supplementary file 11 [file pmed.1005189.s012.pdf]

Appendix 12a. Anti-diabetic medications — sensitivity analysis (validated adherence tools)

Random-effects meta-analysis | k = 56 studies | Total N = 14,976 | Events = 8,571

Events / N	Study	Proportion [95% CI]
82 / 469	Kamuhabwa 2014	17% [14%, 21%]
30 / 155	Bizu 2016	19% [13%, 26%]
60 / 303	Jackson 2015	20% [15%, 25%]
93 / 403	Sendekie 2022	23% [19%, 28%]
66 / 273	Mariye 2019	24% [19%, 30%]
22 / 89	Ikolaba 2023	25% [16%, 35%]
66 / 226	Adisa 2016	29% [23%, 36%]
63 / 188	Kretchy 2020	34% [27%, 41%]
142 / 422	Wondm 2024	34% [29%, 38%]
136 / 400	Tefera 2020	34% [29%, 39%]
116 / 321	Araya 2020	36% [31%, 42%]
115 / 309	Kassahun 2016a (N=309)	37% [32%, 43%]
77 / 200	Bruce 2015	38% [32%, 46%]
158 / 404	Owolabi 2024	39% [34%, 44%]
82 / 200	Yusuff 2008	41% [34%, 48%]
32 / 75	Ojieabu 2017	43% [31%, 55%]
131 / 289	Waari 2018	45% [39%, 51%]
89 / 195	Aminde 2019	46% [39%, 53%]
141 / 305	Dimore 2023	46% [41%, 52%]
93 / 200	Murwanashyaka 2022	46% [39%, 54%]
86 / 176	Adisa 2013	49% [41%, 56%]
193 / 376	Lalam 2026	51% [46%, 56%]
91 / 172	Adisa 2014	53% [45%, 61%]
110 / 201	Ipingbemi 2021	55% [48%, 62%]
103 / 188	Aje 2024	55% [47%, 62%]
80 / 146	Ali 2017	55% [46%, 63%]
169 / 305	Hadush 2024	55% [50%, 61%]
108 / 182	Tewabe 2018	59% [52%, 67%]
69 / 114	Adisa 2011	61% [51%, 70%]
252 / 411	Zemen 2025	61% [56%, 66%]
118 / 191	Boshe 2021b (N=191)	62% [54%, 69%]
186 / 300	Ibrahim 2021	62% [56%, 68%]
124 / 200	Adisa 2017	62% [55%, 69%]
157 / 248	Doya 2024	63% [57%, 69%]
126 / 191	Boshe 2021a (N=191)	66% [59%, 73%]
265 / 396	Mutyambizi 2020	67% [62%, 72%]
125 / 186	Sefah 2025	67% [60%, 74%]
196 / 285	Kassahun 2016b (N=285)	69% [63%, 74%]
137 / 196	Adegbola 2016	70% [63%, 76%]
194 / 275	Ayele 2019b (N=275)	71% [65%, 76%]
562 / 794	Simegn 2023	71% [67%, 74%]
153 / 215	Rwegerera 2014	71% [65%, 77%]
258 / 359	Tusa 2020	72% [67%, 76%]
105 / 145	Omotosho 2024	72% [64%, 80%]
87 / 120	Pascal 2012	72% [64%, 80%]
268 / 357	Demoz 2020	75% [70%, 79%]
247 / 327	AT Kassaw 2025	76% [71%, 80%]
202 / 267	Teklay 2013	76% [70%, 81%]
67 / 87	Billoro 2022	77% [67%, 85%]
434 / 521	Bagonza 2015	83% [80%, 86%]
163 / 194	Sorato 2016	84% [78%, 89%]
279 / 330	Afaya 2020	85% [80%, 88%]
404 / 422	Bonger 2018	96% [93%, 97%]
351 / 363	Alenbalu 2024	97% [94%, 98%]
91 / 93	Gemeda 2022	98% [92%, 100%]
217 / 217	Degefa 2020	100% [98%, 100%]
	**Pooled**	**60% [52%, 67%] I² = 99% (k = 56)**

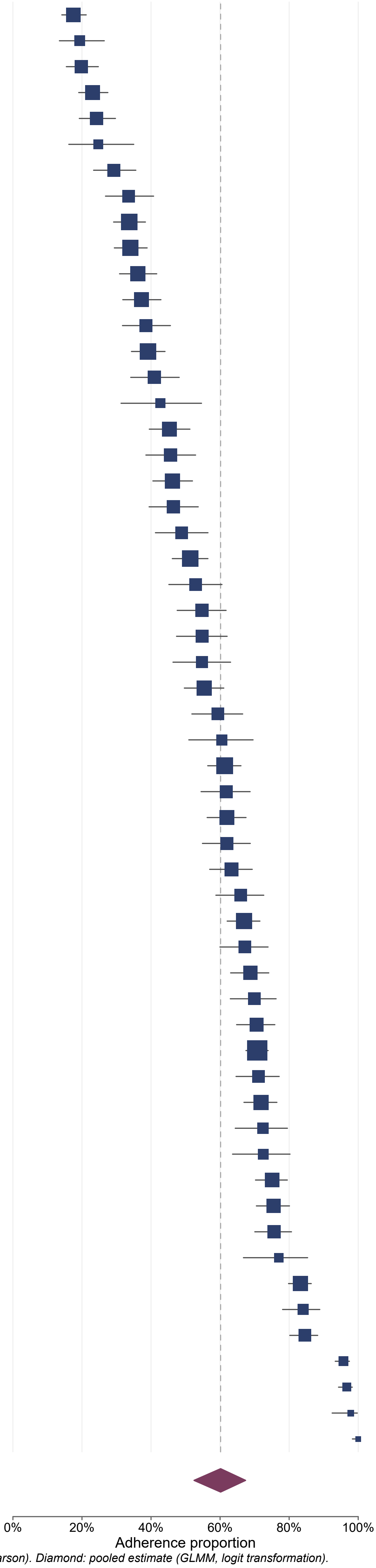

Squares: study estimates (size ∝ inverse SE). Horizontal lines: 95% CI (Clopper–Pearson). Diamond: pooled estimate (GLMM, logit transformation).

Appendix 12b. Anti-hypertensive medications — sensitivity analysis (validated adherence tools)

Random-effects meta-analysis | k = 54 studies | Total N = 18,635 | Events = 8,692

Events / N	Study	Proportion [95% CI]
5 / 500	Oluwole 2019	1% [0%, 2%]
6 / 400	Makukule 2023	2% [1%, 3%]
10 / 329	Okello 2016	3% [1%, 6%]
6 / 148	Adeoye 2019	4% [2%, 9%]
27 / 400	Kretchy 2014 (Validated scale)	7% [4%, 10%]
54 / 605	Adisa 2018	9% [7%, 11%]
52 / 378	Desta 2024	14% [10%, 18%]
64 / 358	Kretchy 2020	18% [14%, 22%]
194 / 925	Berhe 2017	21% [18%, 24%]
91 / 335	Mebrahtu 2021	27% [22%, 32%]
59 / 183	Adidja 2018	32% [26%, 40%]
119 / 357	Boima 2015	33% [28%, 38%]
356 / 989	Tsadik 2020	36% [33%, 39%]
153 / 422	Onwukwe 2022	36% [32%, 41%]
156 / 422	Abdisa 2022 (Validated scale)	37% [32%, 42%]
41 / 102	Mukora-Mutseyekwa 2013	40% [31%, 50%]
130 / 322	Abdulwahed 2020	40% [35%, 46%]
112 / 277	Attahiru 2023	40% [35%, 46%]
109 / 269	Osei-Yeboah 2019	41% [35%, 47%]
98 / 241	Weldegebreal 2019	41% [34%, 47%]
97 / 221	Akoko 2017	44% [37%, 51%]
33 / 74	Mbaseege 2025	45% [33%, 57%]
86 / 187	Adegoke 2022	46% [39%, 53%]
191 / 410	Simegn 2021	47% [42%, 52%]
215 / 450	Kebede 2024	48% [43%, 53%]
58 / 120	Nashilongo 2017	48% [39%, 58%]
198 / 405	Tebelu 2023	49% [44%, 54%]
212 / 422	Abdisa 2022 (H-SCALE)	50% [45%, 55%]
219 / 422	Melaku 2022	52% [47%, 57%]
190 / 366	Andualem 2021	52% [47%, 57%]
266 / 480	Assefa 2023	55% [51%, 60%]
168 / 300	Maginga 2016	56% [50%, 62%]
53 / 93	Xiong 2023	57% [46%, 67%]
145 / 250	Adisa 2017	58% [52%, 64%]
214 / 357	Yousuf 2025	60% [55%, 65%]
173 / 286	Asgedom 2016	60% [55%, 66%]
173 / 280	Asgedom 2018	62% [56%, 68%]
211 / 341	Labata 2019	62% [56%, 67%]
255 / 402	Rashid 2024	63% [59%, 68%]
225 / 351	Amadu 2025	64% [59%, 69%]
248 / 384	Ambaw 2012	65% [60%, 69%]
275 / 409	Mekonnen 2017	67% [62%, 72%]
160 / 223	Kinfe 2020	72% [65%, 78%]
285 / 393	Elias 2025	73% [68%, 77%]
293 / 396	Aberhe 2020	74% [69%, 78%]
237 / 320	Gebremichael 2019	74% [69%, 79%]
281 / 370	Muhe 2025	76% [71%, 80%]
86 / 112	Sibomana 2019	77% [68%, 84%]
303 / 392	Teshome 2018	77% [73%, 81%]
136 / 172	Berisa 2018	79% [72%, 85%]
96 / 115	Wake 2021	83% [75%, 90%]
351 / 400	Nakwafila 2022	88% [84%, 91%]
330 / 370	Sarkodie 2020	89% [86%, 92%]
387 / 400	Okunrinboye 2019	97% [95%, 98%]
	**Pooled**	**46% [37%, 56%] I² = 99% (k = 54)**

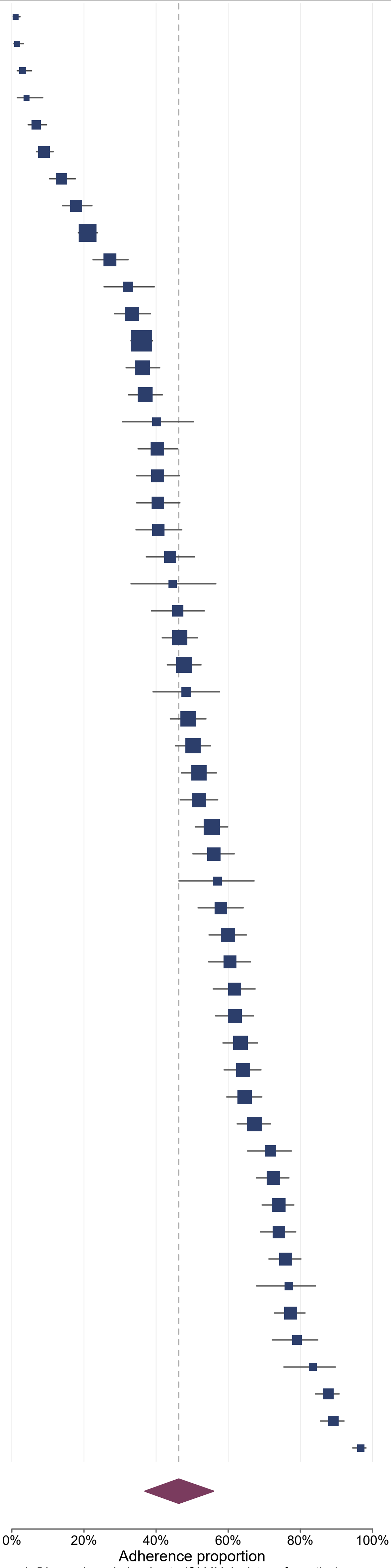

Squares: study estimates (size ∝ inverse SE). Horizontal lines: 95% CI (Clopper–Pearson). Diamond: pooled estimate (GLMM, logit transformation).

Appendix 12c. Dietary recommendations — sensitivity analysis (validated adherence tools)

Random-effects meta-analysis | k = 23 studies | Total N = 8,571 | Events = 3,787

Events / N	Study	Proportion [95% CI]
36 / 480	Assefa 2023	8% [5%, 10%]
38 / 422	Melaku 2022	9% [6%, 12%]
38 / 398	Duodu 2025	10% [7%, 13%]
82 / 320	Ayele 2018	26% [21%, 31%]
113 / 399	Abera 2024	28% [24%, 33%]
92 / 300	Berhe 2017	31% [25%, 36%]
215 / 600	Hailu 2025	36% [32%, 40%]
115 / 307	Adem 2020	37% [32%, 43%]
169 / 387	Bekele 2024	44% [39%, 49%]
284 / 570	MolalignTakele 2021	50% [46%, 54%]
72 / 142	Wilson 2024	51% [42%, 59%]
413 / 794	Simegn 2023	52% [48%, 56%]
185 / 354	Wolderufael 2021	52% [47%, 58%]
113 / 207	Degefa 2020	55% [48%, 62%]
180 / 328	Zewdie 2022	55% [49%, 60%]
290 / 497	Belew 2025	58% [54%, 63%]
116 / 194	Sorato 2016	60% [53%, 67%]
226 / 364	Muhe 2025	62% [57%, 67%]
246 / 396	Mutyambizi 2020	62% [57%, 67%]
202 / 320	Gebremichael 2019	63% [58%, 68%]
60 / 93	Gemeda 2022	65% [54%, 74%]
272 / 398	Gebermariam 2020	68% [64%, 73%]
230 / 301	Andualem 2020	76% [71%, 81%]
	**Pooled**	**44% [34%, 54%] I² = 99% (k = 23)**

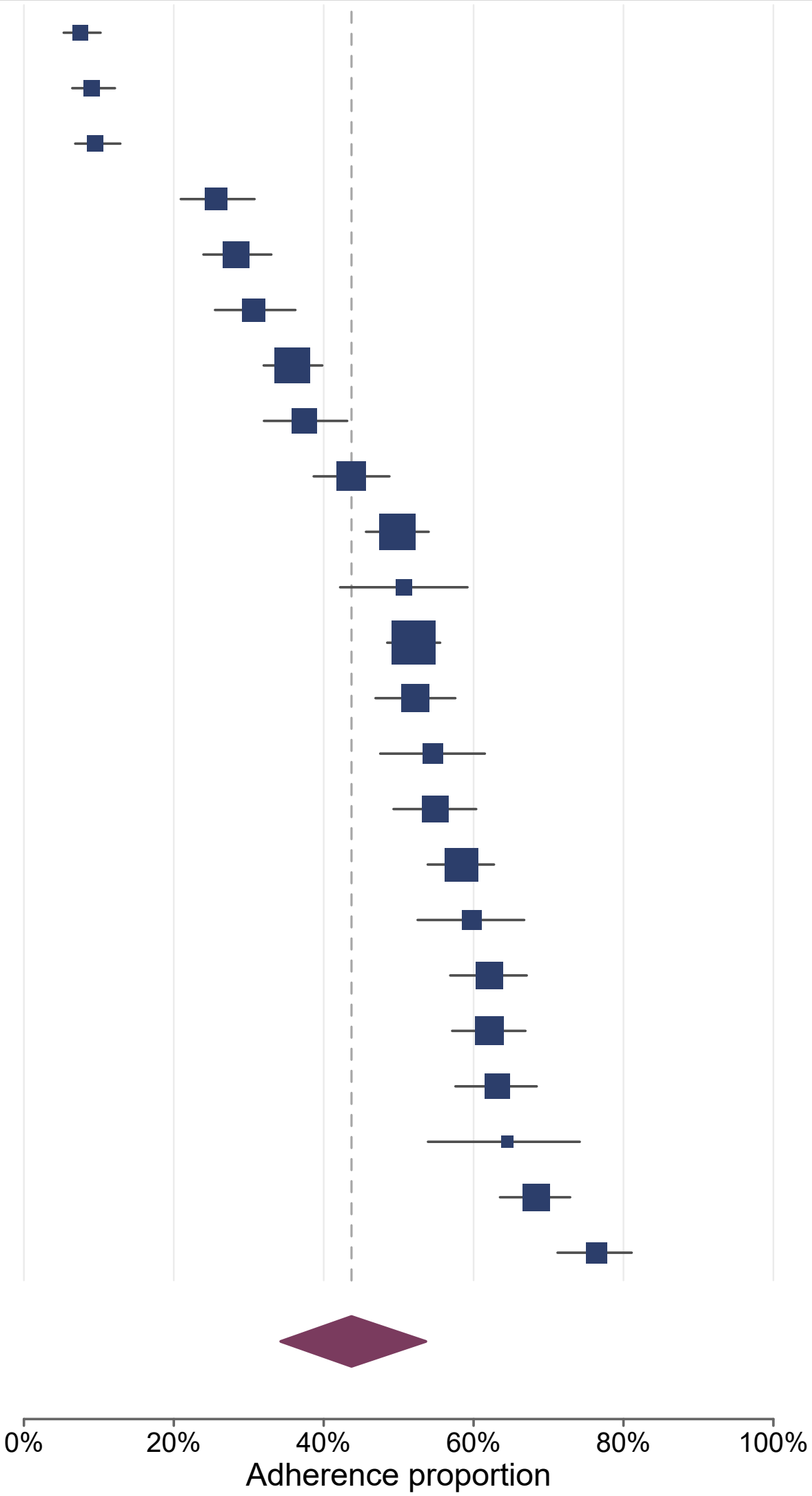

Squares: study estimates (size ∝ inverse SE). Horizontal lines: 95% CI (Clopper–Pearson). Diamond: pooled estimate (GLMM, logit transformation).

Appendix 12d. Physical activity recommendations — sensitivity analysis (validated adherence tools)

Random-effects meta-analysis | k = 31 studies | Total N = 10,050 | Events = 4,281

Events / N	Study	Proportion [95% CI]
36 / 396	Mutyambizi 2020	9% [6%, 12%]
83 / 480	Assefa 2023	17% [14%, 21%]
54 / 305	Dimore 2023	18% [14%, 22%]
68 / 314	Kitaw 2023	22% [17%, 27%]
31 / 140	Bofu 2024	22% [16%, 30%]
125 / 422	Abdisa 2022	30% [25%, 34%]
110 / 350	Tozivepi 2021	31% [27%, 37%]
127 / 378	Desta 2024	34% [29%, 39%]
120 / 328	Kindarara 2025	37% [31%, 42%]
122 / 320	Ayele 2018	38% [33%, 44%]
81 / 207	Degefa 2020	39% [32%, 46%]
161 / 403	Gulentie 2020	40% [35%, 45%]
39 / 97	Taylor 2023	40% [30%, 51%]
246 / 600	Hailu 2025	41% [37%, 45%]
168 / 405	Tebelu 2023	41% [37%, 46%]
125 / 286	Hareri 2013	44% [38%, 50%]
174 / 392	Teshome 2018	44% [39%, 49%]
153 / 341	Labata 2019	45% [40%, 50%]
124 / 276	Niriayo 2019	45% [39%, 51%]
64 / 141	Night 2023	45% [37%, 54%]
188 / 411	Zemen 2025	46% [41%, 51%]
194 / 419	Bonger 2018	46% [41%, 51%]
158 / 320	Gebremichael 2019	49% [44%, 55%]
196 / 387	Bekele 2024	51% [46%, 56%]
47 / 84	Banson 2023	56% [45%, 67%]
299 / 450	Kebede 2024	66% [62%, 71%]
222 / 332	Edmealem 2020	67% [62%, 72%]
82 / 119	Oyewole 2014	69% [60%, 77%]
104 / 150	Osei-Yeboah 2019	69% [61%, 77%]
267 / 374	Mbambo 2019	71% [67%, 76%]
313 / 423	Oluma 2020 [SURFACED]	74% [70%, 78%]
	**Pooled**	**42% [36%, 49%] I² = 98% (k = 31)**

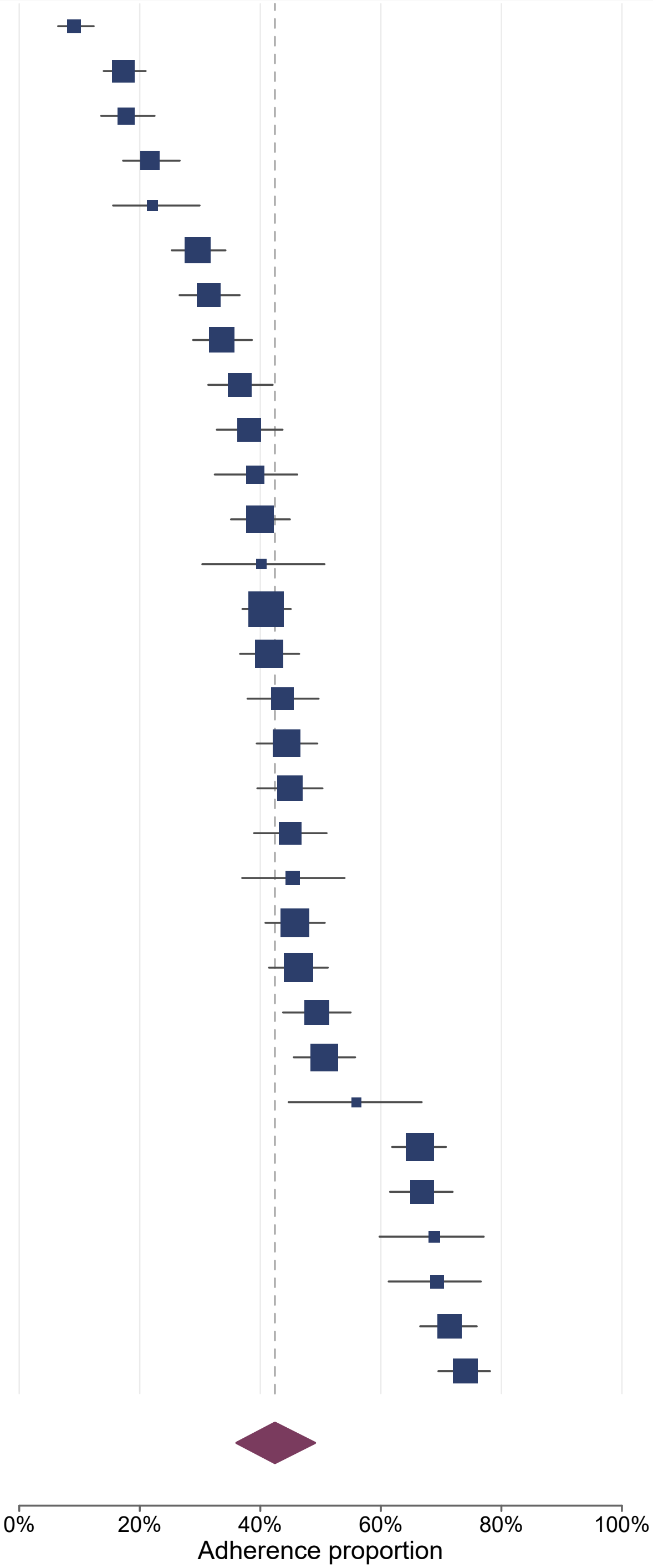

Squares: study estimates (size ∝ inverse SE). Horizontal lines: 95% CI (Clopper–Pearson). Diamond: pooled estimate (GLMM, logit transformation).

Appendix 12e. Alcohol recommendations — sensitivity analysis (validated adherence tools)

Random-effects meta-analysis | k = 13 studies | Total N = 4,484 | Events = 3,524

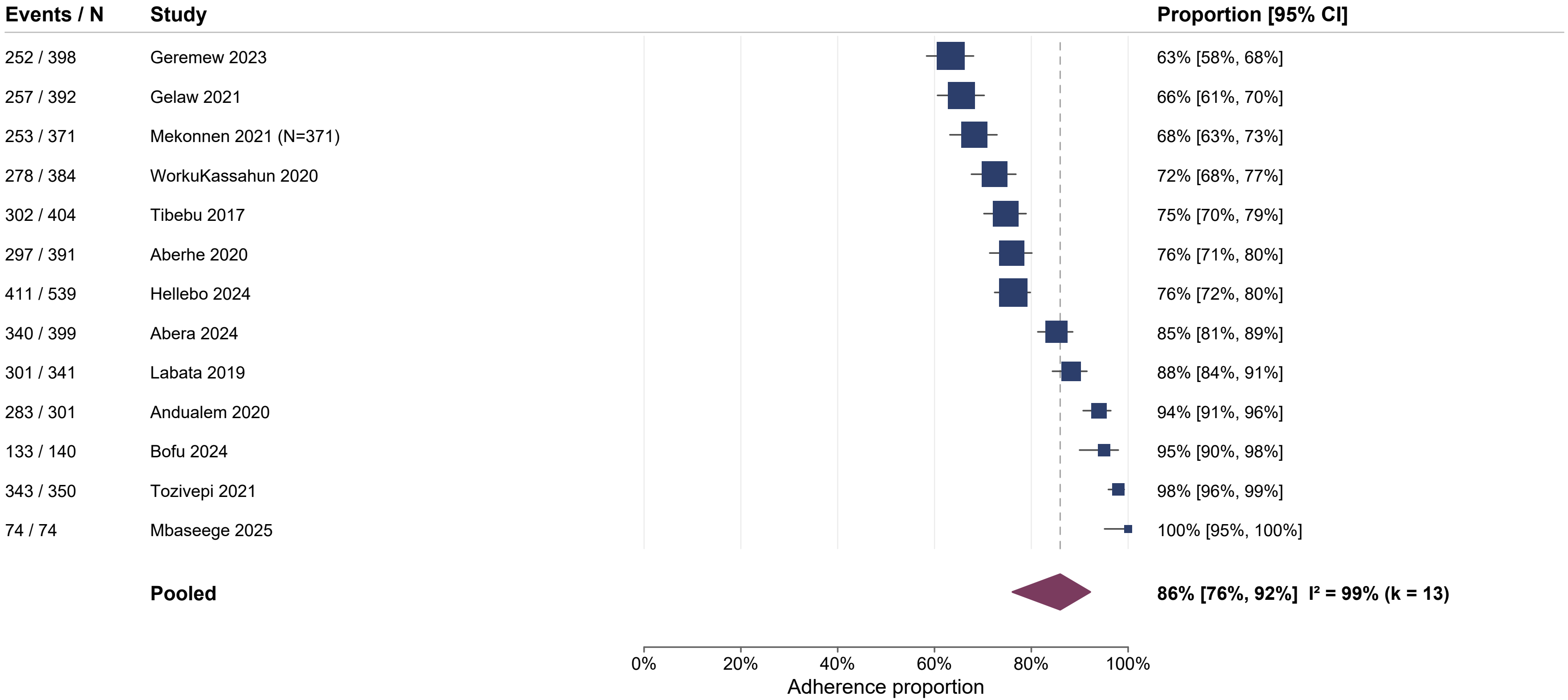

Squares: study estimates (size ∝ inverse SE). Horizontal lines: 95% CI (Clopper–Pearson). Diamond: pooled estimate (GLMM, logit transformation).

Appendix 12g. Blood glucose self-monitoring — sensitivity analysis (validated adherence tools)

Random-effects meta-analysis | k = 10 studies | Total N = 3,457 | Events = 1,089

Events / N	Study	Proportion [95% CI]
18 / 328	Kindarara 2025	5% [3%, 9%]
25 / 318	Ugwu 2018	8% [5%, 11%]
19 / 140	Bofu 2024	14% [8%, 20%]
156 / 600	Endale 2025	26% [23%, 30%]
61 / 217	Degefa 2020	28% [22%, 35%]
267 / 794	Simegn 2023	34% [30%, 37%]
32 / 93	Gemeda 2022	34% [25%, 45%]
84 / 226	Ewunetu 2025	37% [31%, 44%]
198 / 354	Wolderufael 2021	56% [51%, 61%]
229 / 387	Bekele 2024	59% [54%, 64%]
	**Pooled**	**26% [16%, 40%] I² = 98% (k = 10)**

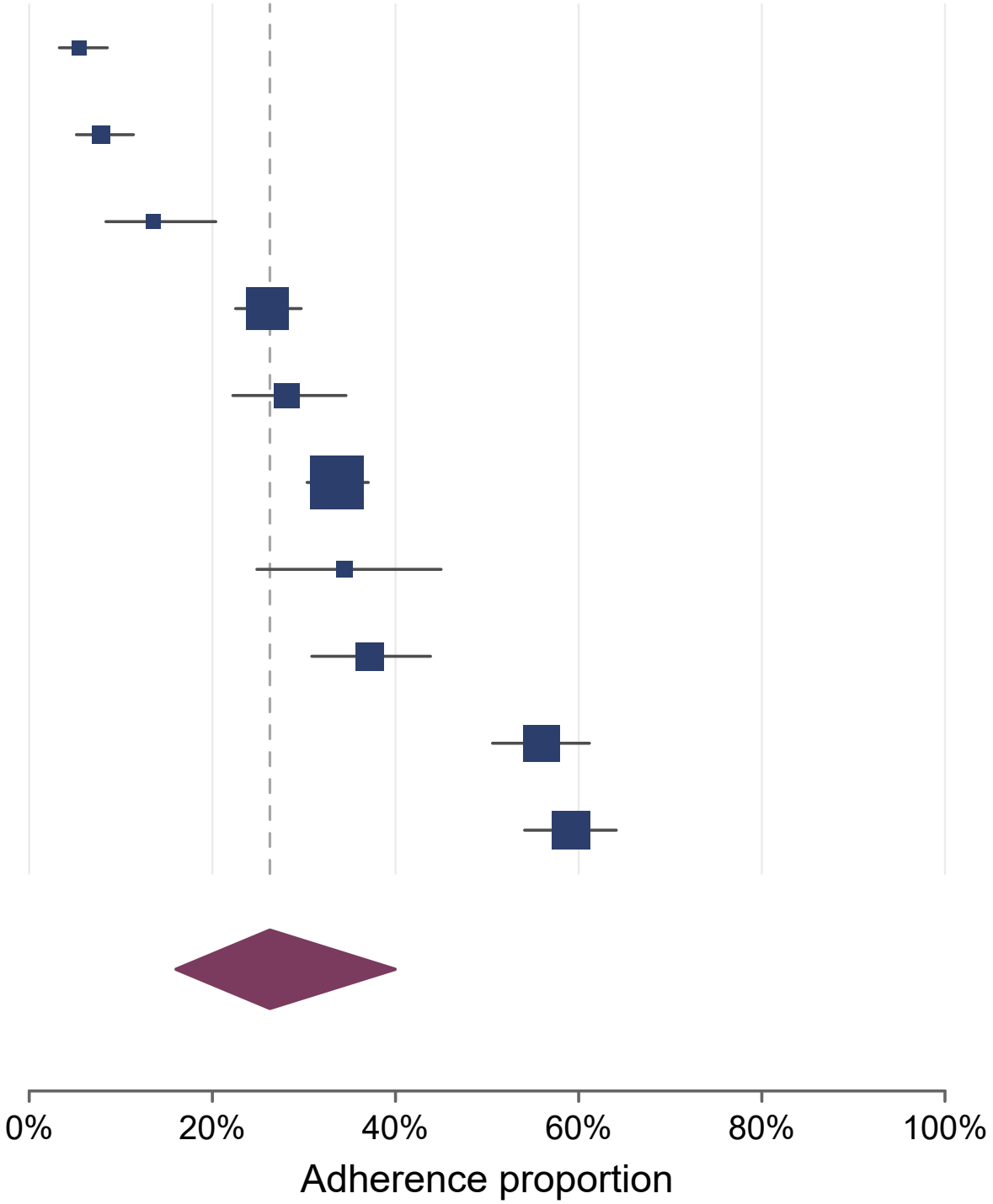

Squares: study estimates (size ∝ inverse SE). Horizontal lines: 95% CI (Clopper–Pearson). Diamond: pooled estimate (GLMM, logit transformation).

Appendix 12i. Overall diabetes self-care — sensitivity analysis (validated adherence tools)

Random-effects meta-analysis | k = 25 studies | Total N = 9,213 | Events = 3,734

Events / N	Study	Proportion [95% CI]
5 / 217	Okonta 2014	2% [1%, 5%]
20 / 402	Zwane 2023	5% [3%, 8%]
12 / 140	Bofu 2024	9% [5%, 14%]
51 / 422	Wondm 2024	12% [9%, 16%]
73 / 345	Miyashita 2024	21% [17%, 26%]
240 / 726	Kiarie 2023	33% [30%, 37%]
106 / 284	Mariye 2018	37% [32%, 43%]
122 / 320	Ayele 2019	38% [33%, 44%]
152 / 384	Emire 2022	40% [35%, 45%]
80 / 194	Sorato 2016	41% [34%, 49%]
337 / 794	Simegn 2023	42% [39%, 46%]
262 / 600	Endale 2025	44% [40%, 48%]
178 / 387	Bekele 2024	46% [41%, 51%]
266 / 570	MolalignTakele 2021	47% [43%, 51%]
99 / 207	Degefa 2020	48% [41%, 55%]
178 / 371	Jemal 2022	48% [43%, 53%]
170 / 354	Wolderufael 2021	48% [43%, 53%]
152 / 309	Kassahun 2016	49% [43%, 55%]
29 / 58	Gurmu 2023	50% [37%, 63%]
173 / 344	Zewdie 2022	50% [45%, 56%]
153 / 300	Berhe 2017	51% [45%, 57%]
140 / 257	Gurmu 2018	54% [48%, 61%]
249 / 432	Eshete 2023	58% [53%, 62%]
244 / 411	Zemen 2025	59% [54%, 64%]
243 / 385	Tiruneh 2019	63% [58%, 68%]
	**Pooled**	**36% [28%, 46%] I² = 99% (k = 25)**

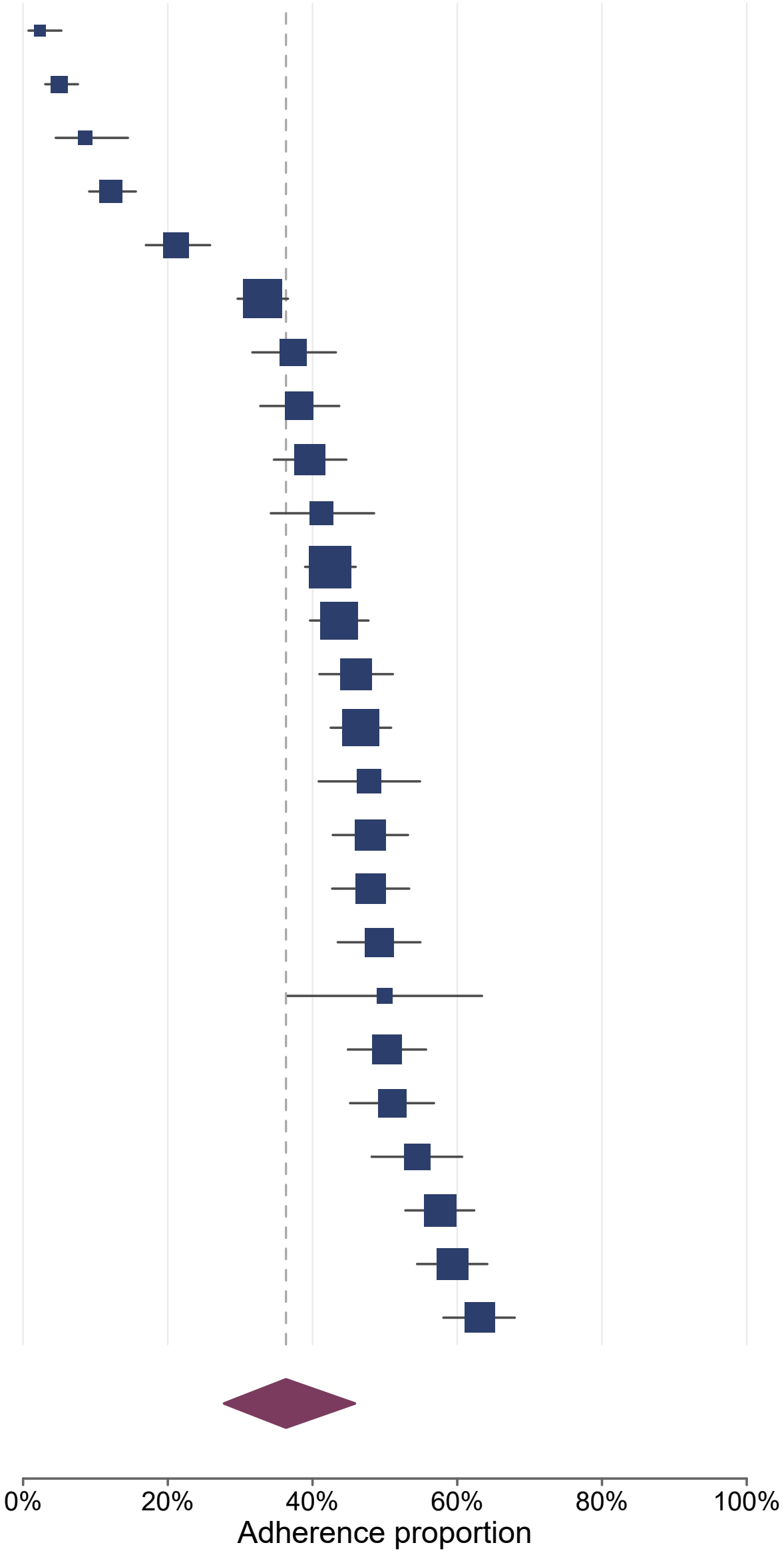

Squares: study estimates (size ∝ inverse SE). Horizontal lines: 95% CI (Clopper–Pearson). Diamond: pooled estimate (GLMM, logit transformation).

Appendix 12j. Overall hypertension self-care — sensitivity analysis (validated adherence tools)

Random-effects meta-analysis | k = 26 studies | Total N = 8,812 | Events = 3,351

Events / N	Study	Proportion [95% CI]
18 / 350	Tozivepi 2021	5% [3%, 8%]
27 / 224	Kimani 2019	12% [8%, 17%]
63 / 330	Philbert 2023	19% [15%, 24%]
65 / 320	Gebremichael 2019	20% [16%, 25%]
96 / 416	Tibebu 2017	23% [19%, 27%]
71 / 301	Andualem 2020	24% [19%, 29%]
57 / 210	Buda 2017	27% [21%, 34%]
109 / 370	Muhe 2025	29% [25%, 34%]
117 / 391	Hussen 2020	30% [25%, 35%]
129 / 398	Geremew 2023	32% [28%, 37%]
38 / 115	Wake 2021	33% [25%, 42%]
134 / 405	Tebelu 2023	33% [29%, 38%]
189 / 567	Miyashita 2024 (HTN)	33% [29%, 37%]
27 / 74	Mbaseege 2025	36% [26%, 48%]
157 / 405	Kebede 2022	39% [34%, 44%]
51 / 120	Nashilongo 2017	42% [34%, 52%]
144 / 322	Abdulwahed 2020	45% [39%, 50%]
198 / 422	Melaku 2022	47% [42%, 52%]
219 / 450	Kebede 2024	49% [44%, 53%]
151 / 309	Ademe 2019	49% [43%, 55%]
235 / 480	Assefa 2023	49% [44%, 54%]
219 / 422	Abdisa 2022	52% [47%, 57%]
212 / 392	Gelaw 2021	54% [49%, 59%]
244 / 413	Gebresilase 2024	59% [54%, 64%]
228 / 384	WorkuKassahun 2020	59% [54%, 64%]
153 / 222	Fetensa 2019	69% [62%, 75%]
	**Pooled**	**36% [29%, 43%] I² = 98% (k = 26)**

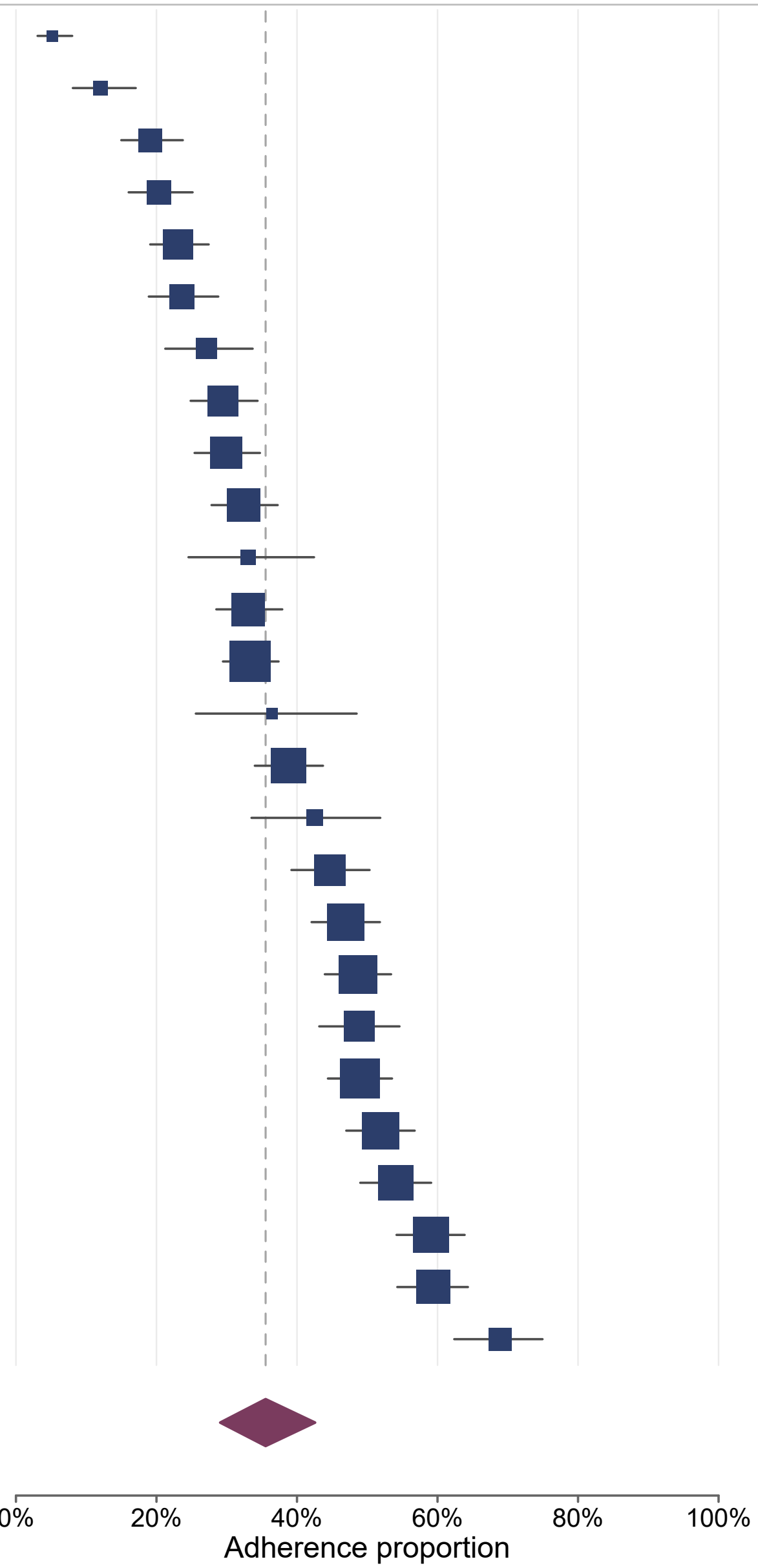

Squares: study estimates (size ∝ inverse SE). Horizontal lines: 95% CI (Clopper–Pearson). Diamond: pooled estimate (GLMM, logit transformation).
