# Supplementary material for: Adherence to medications, lifestyle advice, and self-monitoring for type 2 diabetes and hypertension in sub-Saharan Africa: A systematic review, meta-analysis, and interactive network analysis
Source: PLoS Med. 2026 Aug 3;23(8):e1005189. doi: 10.1371/journal.pmed.1005189 (PMC13460740; doi:10.1371/journal.pmed.1005189)
Supplement: Supplementary file 12 [file pmed.1005189.s014.pdf]

Appendix 14a. Risk of bias assessment for pooled observational studies

Adherence type	Low n (%)	Moderate n (%)	High n (%)	Total (n)
**Medication adherence**				
Anti-diabetes medication	49 (68.1)	20 (27.8)	3 (4.2)	72
Anti-hypertensive medication	49 (57.0)	33 (38.4)	4 (4.7)	86
**Lifestyle advice**				
Dietary recommendation	41 (85.4)	7 (14.6)	0 (0.0)	48
Physical activity recommendation	50 (76.9)	14 (21.5)	1 (1.5)	65
Alcohol recommendation	50 (71.4)	18 (25.7)	2 (2.9)	70
Tobacco smoking	54 (66.7)	22 (27.2)	5 (6.2)	81
**Self-monitoring**				
Self-monitoring for blood glucose	23 (59.0)	13 (33.3)	3 (7.7)	39
Self-monitoring for blood pressure	6 (75.0)	0 (0.0)	2 (25.0)	8
**Self-care practices (all three treatment pillars)**				
Self-care for diabetes (all three treatment pillars)	22 (71.0)	7 (22.6)	2 (6.5)	31
Self-care for hypertension (all three treatment pillars)	23 (79.3)	5 (17.2)	1 (3.4)	29

Summary restricted to the observational studies contributing to the pooled adherence meta-analyses (Appendix 3); percentages are of the rated observational studies in each domain. Risk of bias was assessed using the Newcastle–Ottawa Scale (NOS) and its adapted version for cross-sectional studies, evaluating selection, comparability, and outcome/exposure. Studies were classified as low (7–9 stars for cohort/case–control; 7–10 for cross-sectional), moderate (5–6 stars), or high (0–4 stars) risk of bias. Five pooled studies were matched to Appendix 13 records differing only by publication year or character encoding (Miyashita, Diendéré, Oluma); two without an Appendix 13 record (Feleke 2022, Shilole 2024) were provisionally classified from the published reports (both cross-sectional with random sampling) pending confirmation.

Appendix 14b. Risk of bias assessment for randomised controlled trials (RCTs)

Author and year	Adherence outcome	Risk of Bias 2 (RoB 2) assessment
Ojieabu 2017	Antidiabetic medication adherence	High risk
Odusola 2018	Antihypertensive medication adherence	High risk
Onyinye 2021	Self-care and medications for both diabetes and hypertension	High risk
Jackson 2021	Hypertension self-care and medications	High risk
Adeyemo 2013	Antihypertensive medication adherence	Low risk
Asante 2024	Diabetes self-care and medications	Some concerns
Owolabi 2020	Antidiabetic medication adherence	Some concerns

Author and year	Adherence outcome	Risk of Bias 2 (RoB 2) assessment
Akintola 2025	Antihypertensive medication adherence	Some concerns

Risk of bias was assessed using the revised Cochrane Risk of Bias 2 (RoB 2) tool, evaluating: (1) randomization process, (2) deviations from intended interventions, (3) missing outcome data, (4) measurement of the outcome, and (5) selection of the reported result. Overall risk of bias was categorised as low risk, some concerns, or high risk of bias.
