# Supplementary material for: Adherence to medications, lifestyle advice, and self-monitoring for type 2 diabetes and hypertension in sub-Saharan Africa: A systematic review, meta-analysis, and interactive network analysis
Source: PLoS Med. 2026 Aug 3;23(8):e1005189. doi: 10.1371/journal.pmed.1005189 (PMC13460740; doi:10.1371/journal.pmed.1005189)
Supplement: Supplementary file 13 [file pmed.1005189.s015.pdf]

Appendix 15. Assessment of publication bias — Egger's test for funnel plot asymmetry

Adherence Domain	Number of studies (k)	Total participants	Pooled adherence (Random) % (95% CI)	I^2 (%)	Egger's t-statistic	Degrees of freedom	P-value (Egger's test)
Anti-diabetic medications	73	19,316	67(60–73)	99	3.27	71	0.002
Anti-hypertensive medications	87	31,072	51(44–58)	99	1.10	85	0.273
Dietary recommendations	48	18,184	44(38–49)	98	-1.86	46	0.070
Physical activity recommendations	65	22,438	42(37–47)	98	-1.84	63	0.070
Alcohol recommendations	72	26,546	85(82–88)	98	4.77	70	< 0.001
Smoking recommendations	82	31,985	95(94–96)	96	8.54	80	< 0.001
Blood glucose self-monitoring	39	12,508	18(12–27)	99	-3.90	37	< 0.001
Blood pressure self-monitoring	8	2,354	28(16–45)	98	-0.24	6	0.818
Overall diabetes self-care	31	11,350	37(30–46)	99	-2.63	29	0.014
Overall hypertension self-care	30	10,121	35(29–42)	98	-1.72	28	0.097

Publication bias was assessed using Egger's regression test for funnel-plot asymmetry, applied to logit-transformed proportions with the standard error as predictor (inverse-variance random-effects model). A statistically significant test ($p < 0.05$) suggests small-study effects, which may indicate publication bias. Degrees of freedom equal $k - 2$. Egger's t-statistic and p-value are shown in red where the test is statistically significant ($p < 0.05$, indicating small-study effects) and in green where it is not. Pooled adherence proportions and I^2 are from the random-effects meta-analysis (GLMM, logit transformation), matching the primary analysis (Appendix 3).
