# Supplementary material for: Adherence to medications, lifestyle advice, and self-monitoring for type 2 diabetes and hypertension in sub-Saharan Africa: A systematic review, meta-analysis, and interactive network analysis
Source: PLoS Med. 2026 Aug 3;23(8):e1005189. doi: 10.1371/journal.pmed.1005189 (PMC13460740; doi:10.1371/journal.pmed.1005189)
Supplement: Supplementary file 14 [file pmed.1005189.s016.pdf]

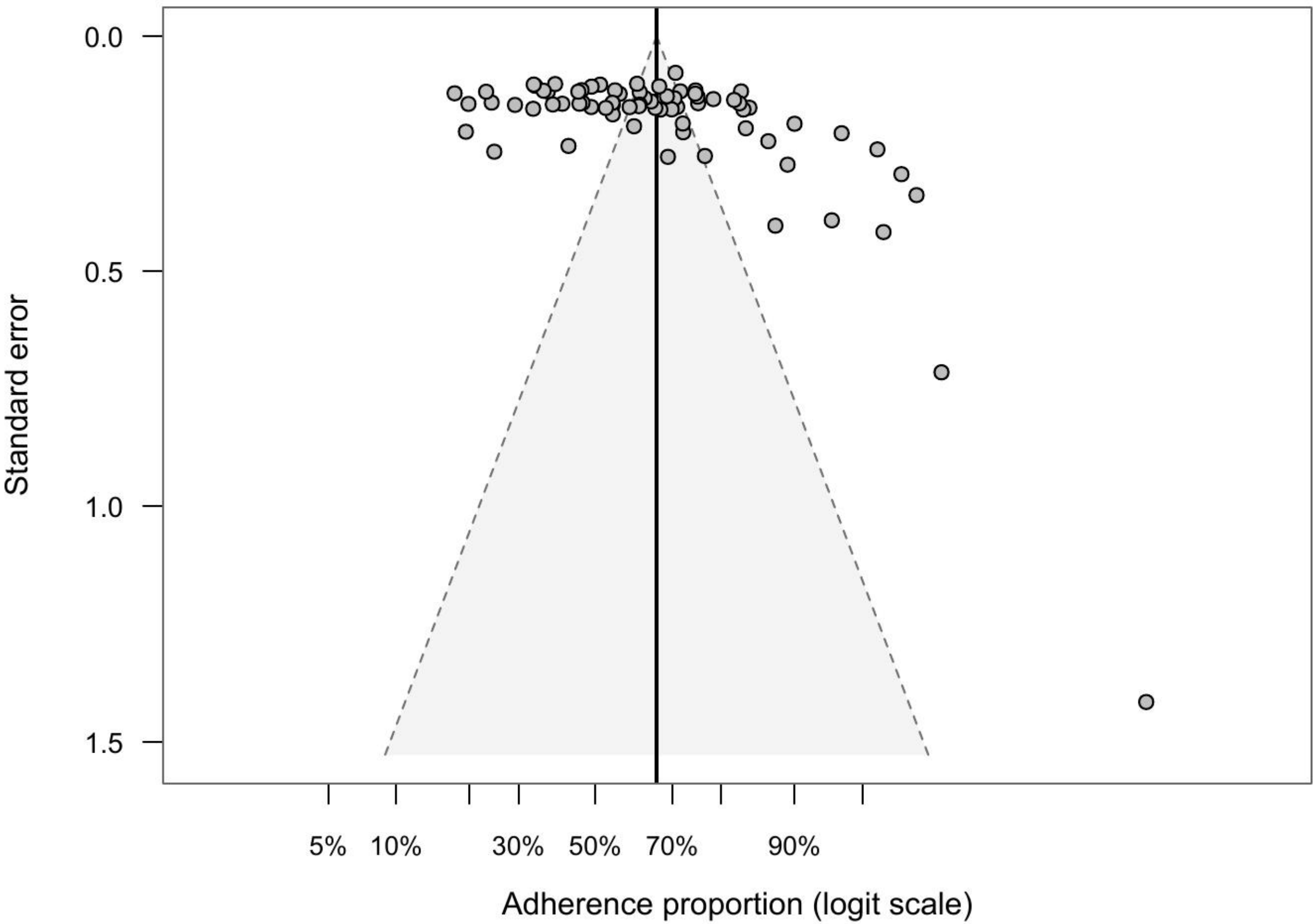

Random-effects meta-analysis | k = 73 studies | Total N = 19,316

Egger's test: t = 3.27, df = 71, p = 0.002

Appendix 16a. Anti-diabetic medications — funnel plot

Solid vertical line: pooled estimate (logit scale). Dashed lines: 95% pseudo-confidence region. Egger's regression test result is annotated above the plot.

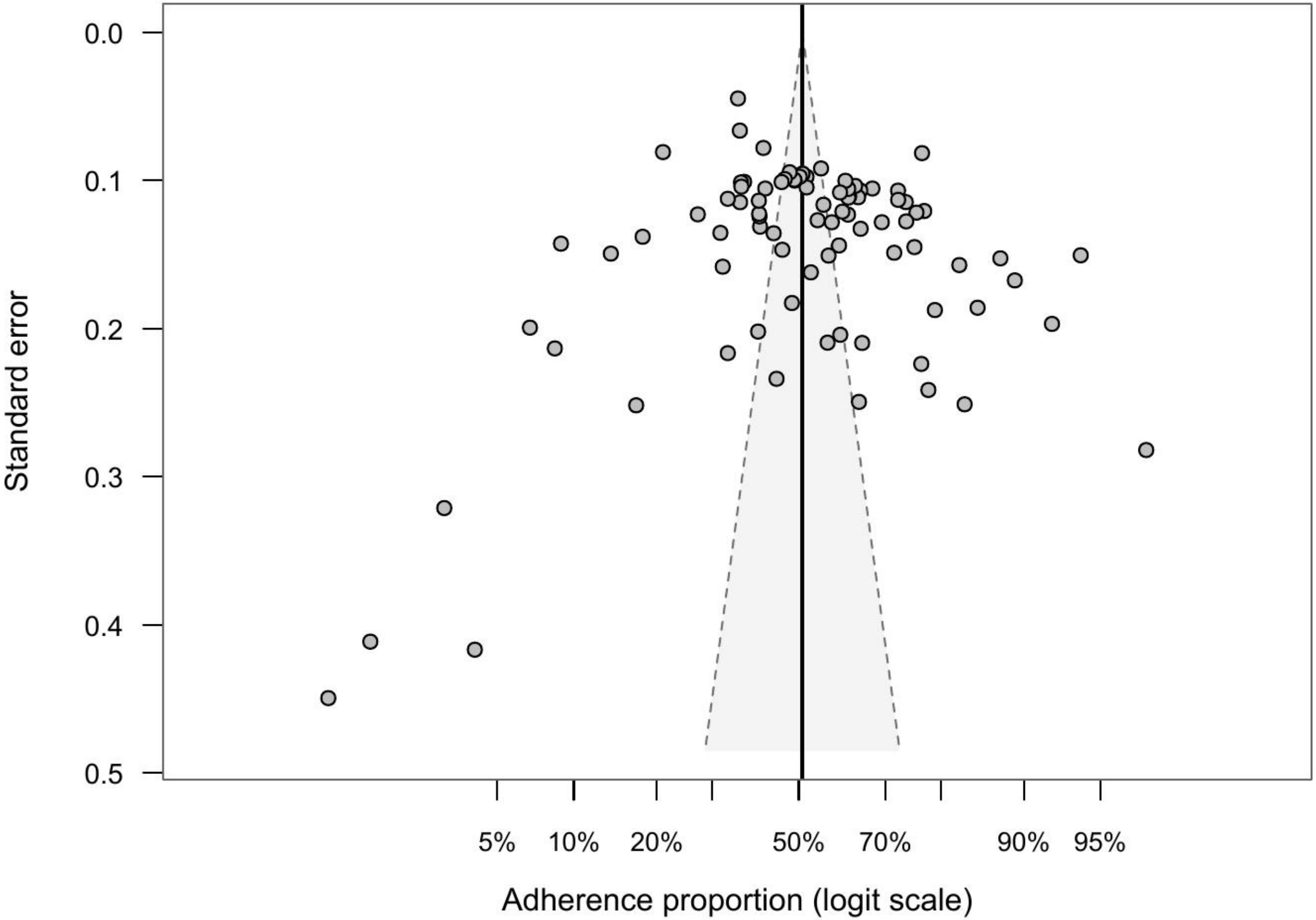

Random-effects meta-analysis | k = 87 studies | Total N = 31,072

Egger's test: t = 1.10, df = 85, p = 0.273

Appendix 16b. Anti-hypertensive medications — funnel plot

Solid vertical line: pooled estimate (logit scale). Dashed lines: 95% pseudo-confidence region. Egger's regression test result is annotated above the plot.

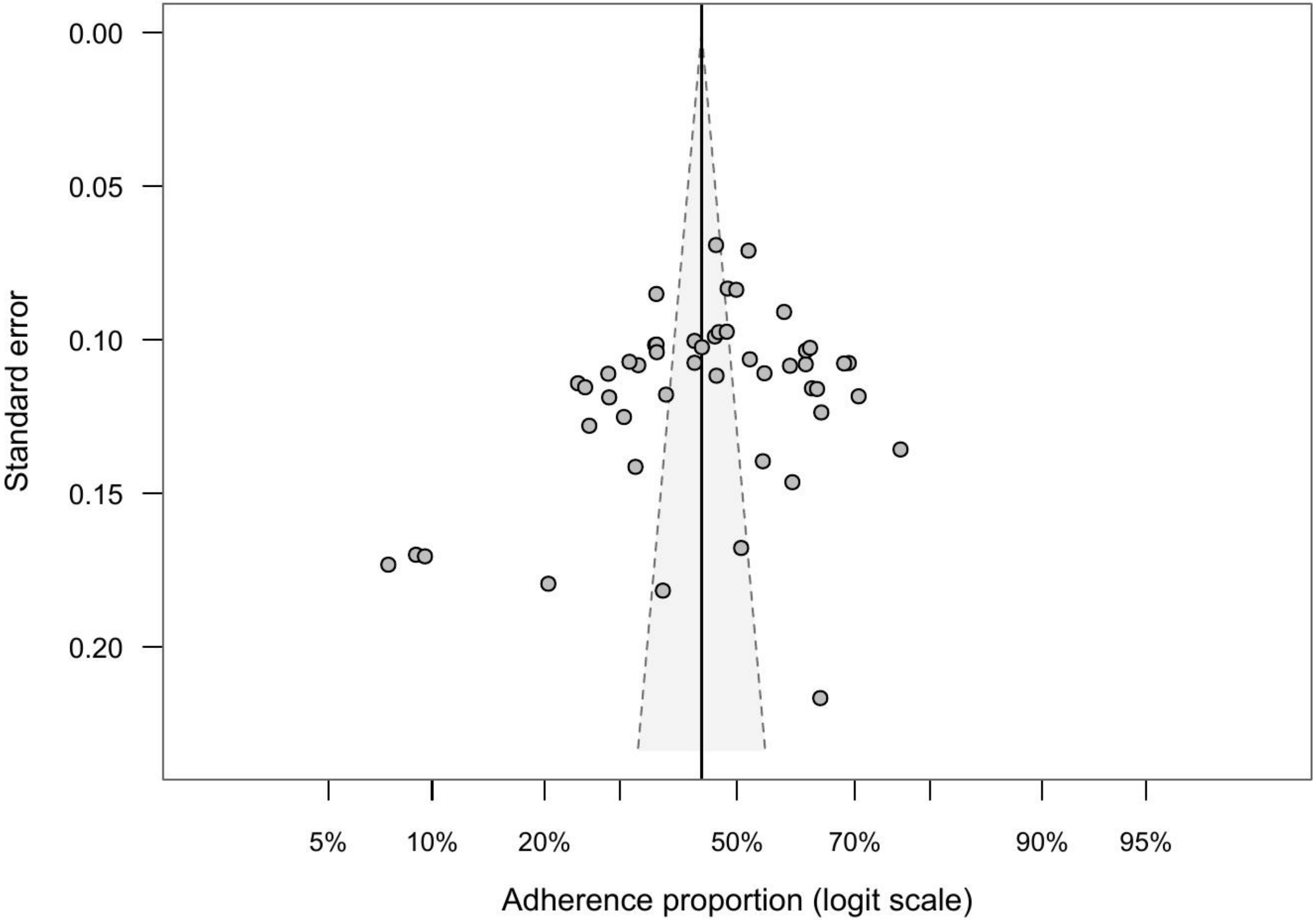

Random-effects meta-analysis | k = 48 studies | Total N = 18,184

Egger's test: t = -1.86, df = 46, p = 0.070

Appendix 16c. Dietary recommendations — funnel plot

Solid vertical line: pooled estimate (logit scale). Dashed lines: 95% pseudo-confidence region. Egger's regression test result is annotated above the plot.

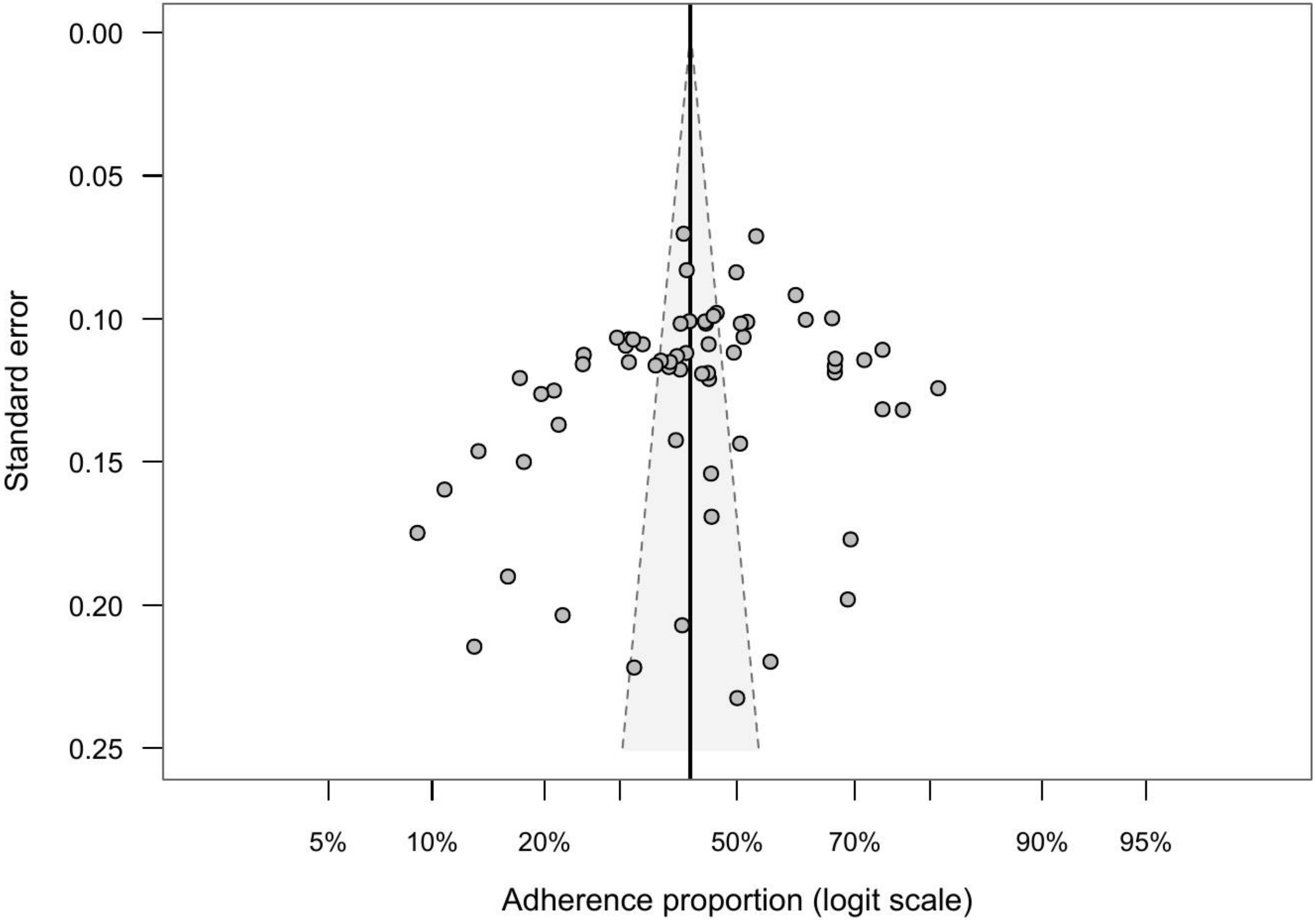

Random-effects meta-analysis | k = 65 studies | Total N = 22,438

Egger's test: t = -1.84, df = 63, p = 0.070

Appendix 16d. Physical activity recommendations — funnel plot

Solid vertical line: pooled estimate (logit scale). Dashed lines: 95% pseudo-confidence region. Egger's regression test result is annotated above the plot.

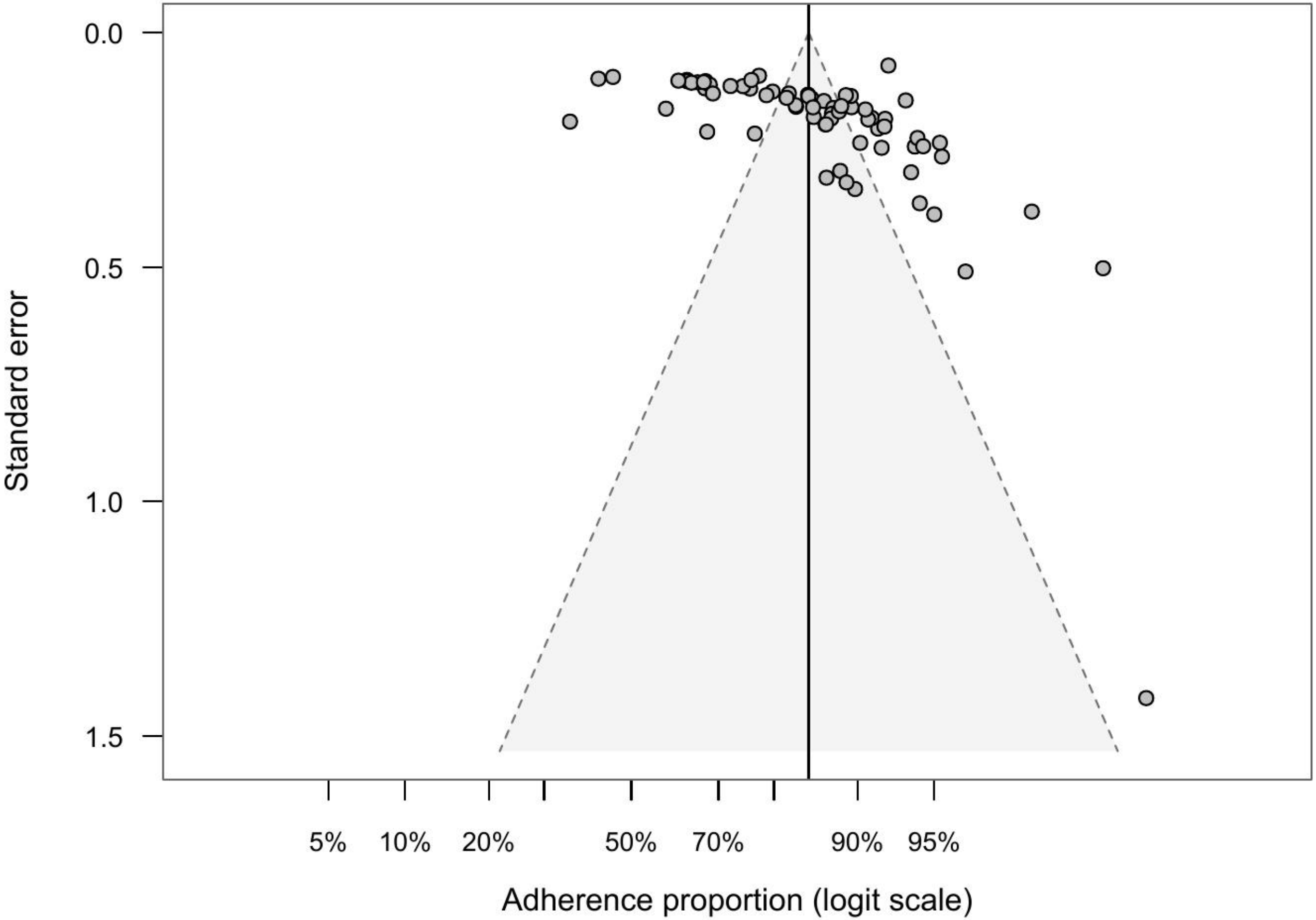

Random-effects meta-analysis | k = 72 studies | Total N = 26,546

Egger's test: t = 4.77, df = 70, p < 0.001

Appendix 16e. Alcohol recommendations — funnel plot

Solid vertical line: pooled estimate (logit scale). Dashed lines: 95% pseudo-confidence region. Egger's regression test result is annotated above the plot.

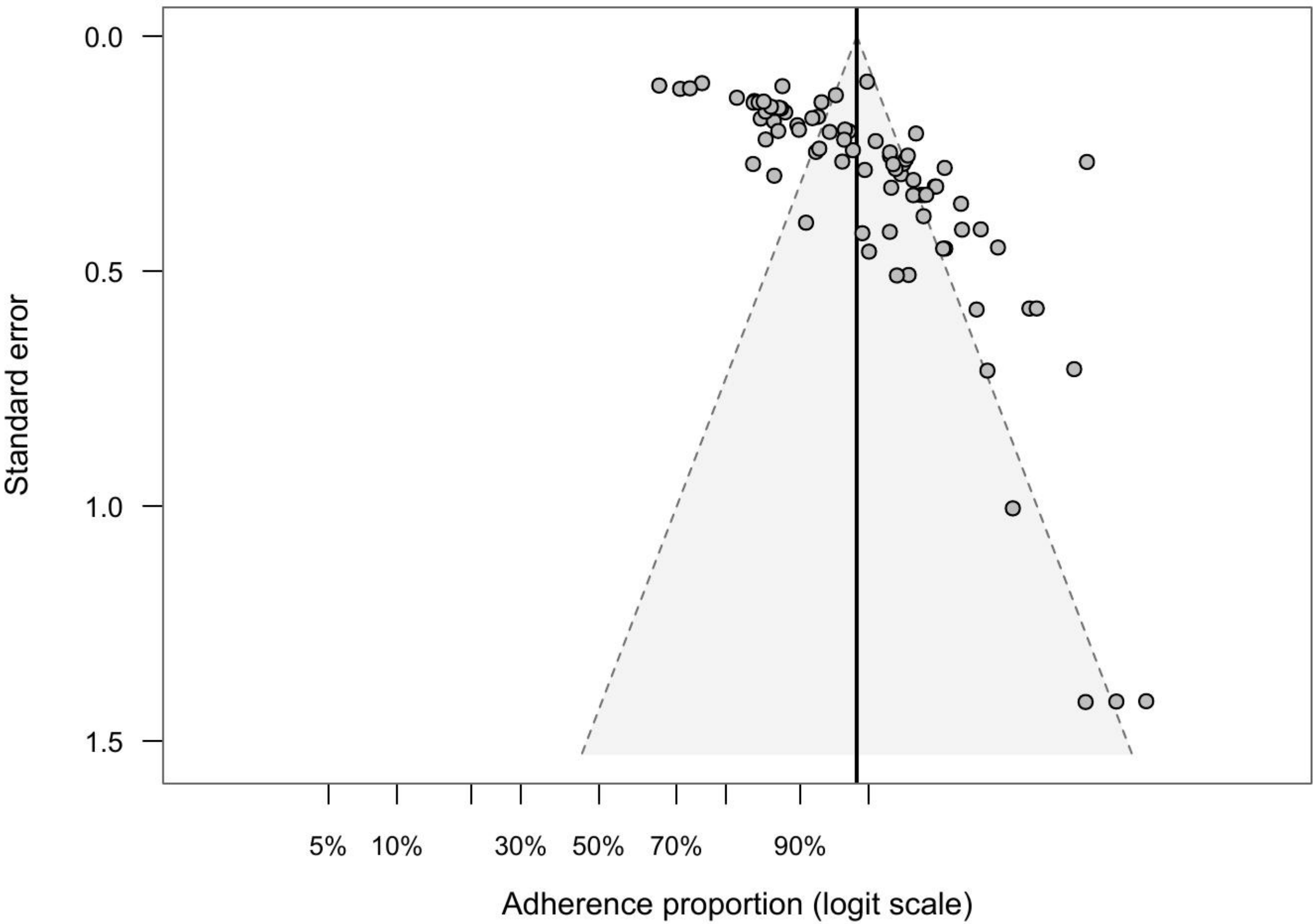

Random-effects meta-analysis | k = 82 studies | Total N = 31,985

Egger's test: t = 8.54, df = 80, p < 0.001

Appendix 16f. Smoking recommendations — funnel plot

Solid vertical line: pooled estimate (logit scale). Dashed lines: 95% pseudo-confidence region. Egger's regression test result is annotated above the plot.

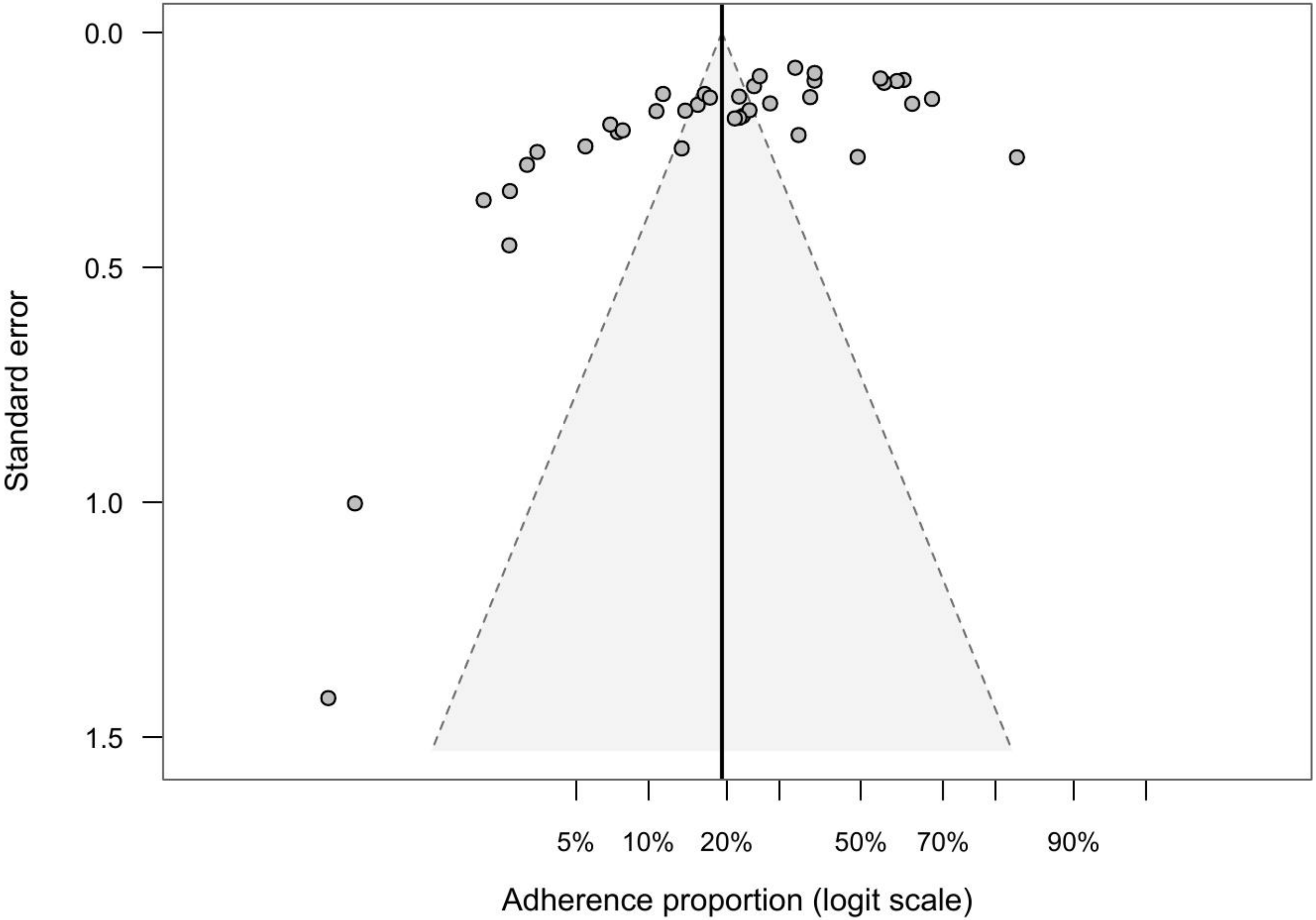

Random-effects meta-analysis | k = 39 studies | Total N = 12,508

Egger's test: t = -3.90, df = 37, p < 0.001

Appendix 16g. Blood glucose self-monitoring — funnel plot

Solid vertical line: pooled estimate (logit scale). Dashed lines: 95% pseudo-confidence region. Egger's regression test result is annotated above the plot.

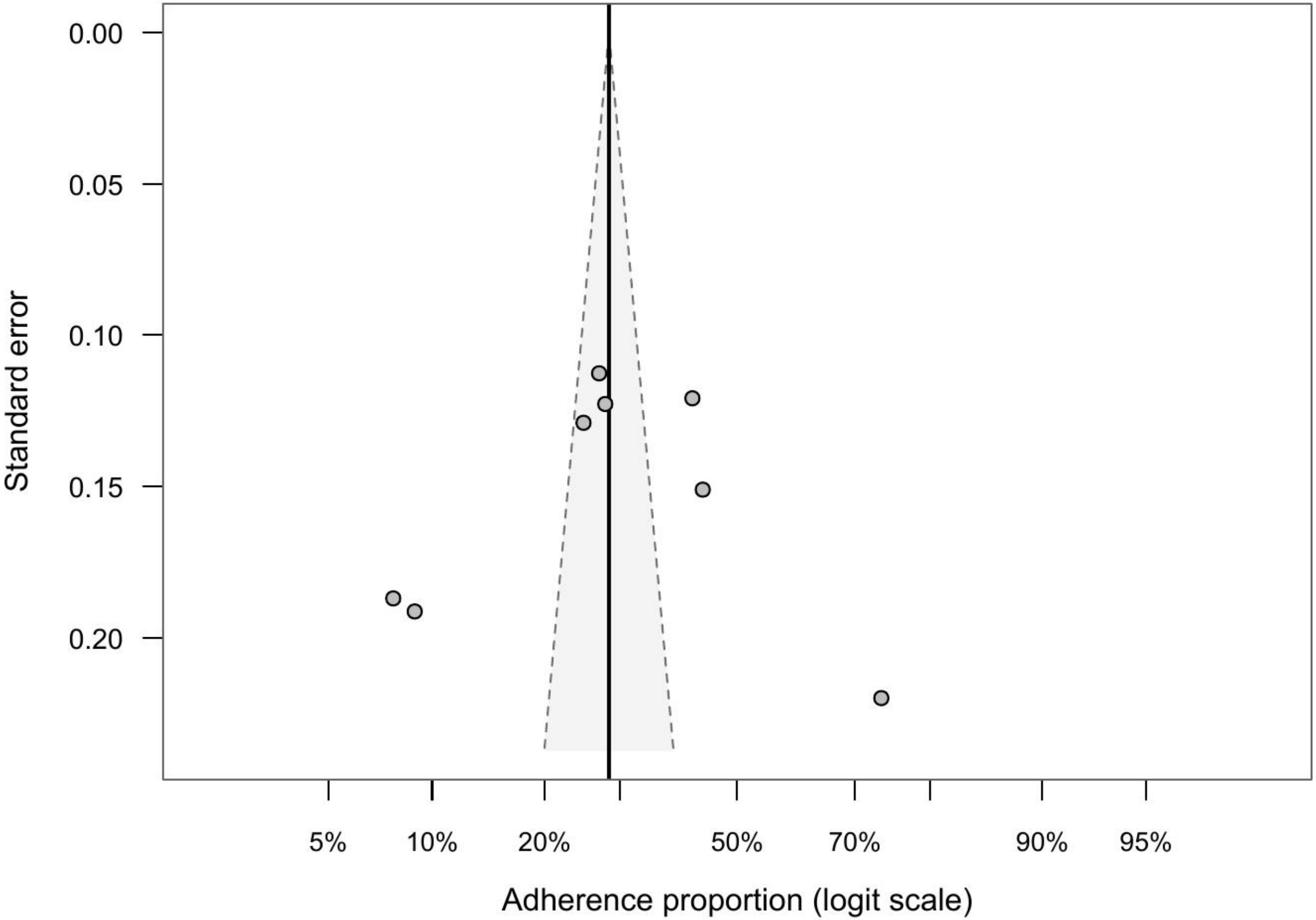

Random-effects meta-analysis | k = 8 studies | Total N = 2,354

Egger's test: t = -0.24, df = 6, p = 0.818

Appendix 16h. Blood pressure self-monitoring — funnel plot

Solid vertical line: pooled estimate (logit scale). Dashed lines: 95% pseudo-confidence region. Egger's regression test result is annotated above the plot.

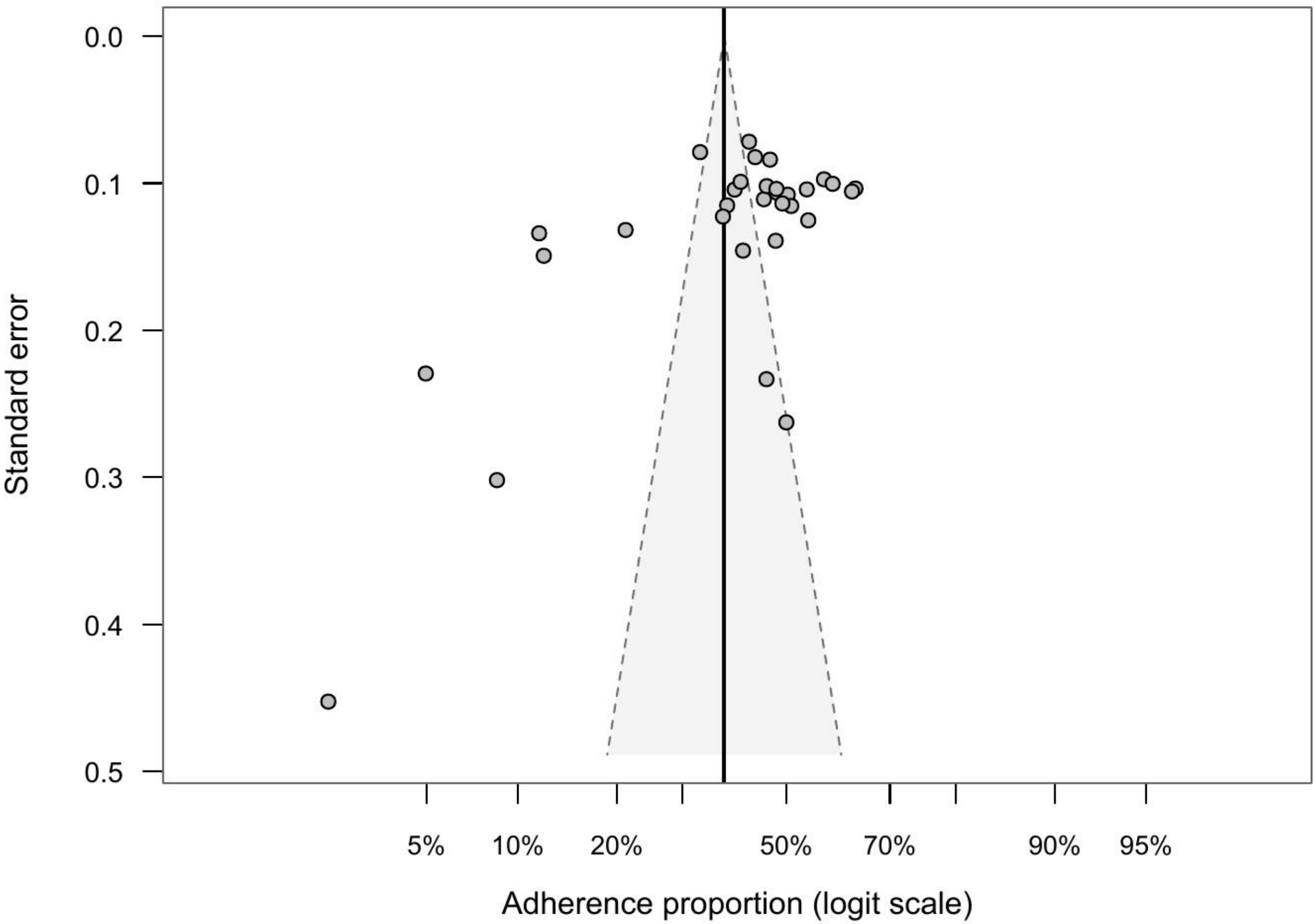

Random-effects meta-analysis | k = 31 studies | Total N = 11,350

Egger's test: t = -2.63, df = 29, p = 0.014

Appendix 16i. Overall diabetes self-care — funnel plot

Solid vertical line: pooled estimate (logit scale). Dashed lines: 95% pseudo-confidence region. Egger's regression test result is annotated above the plot.

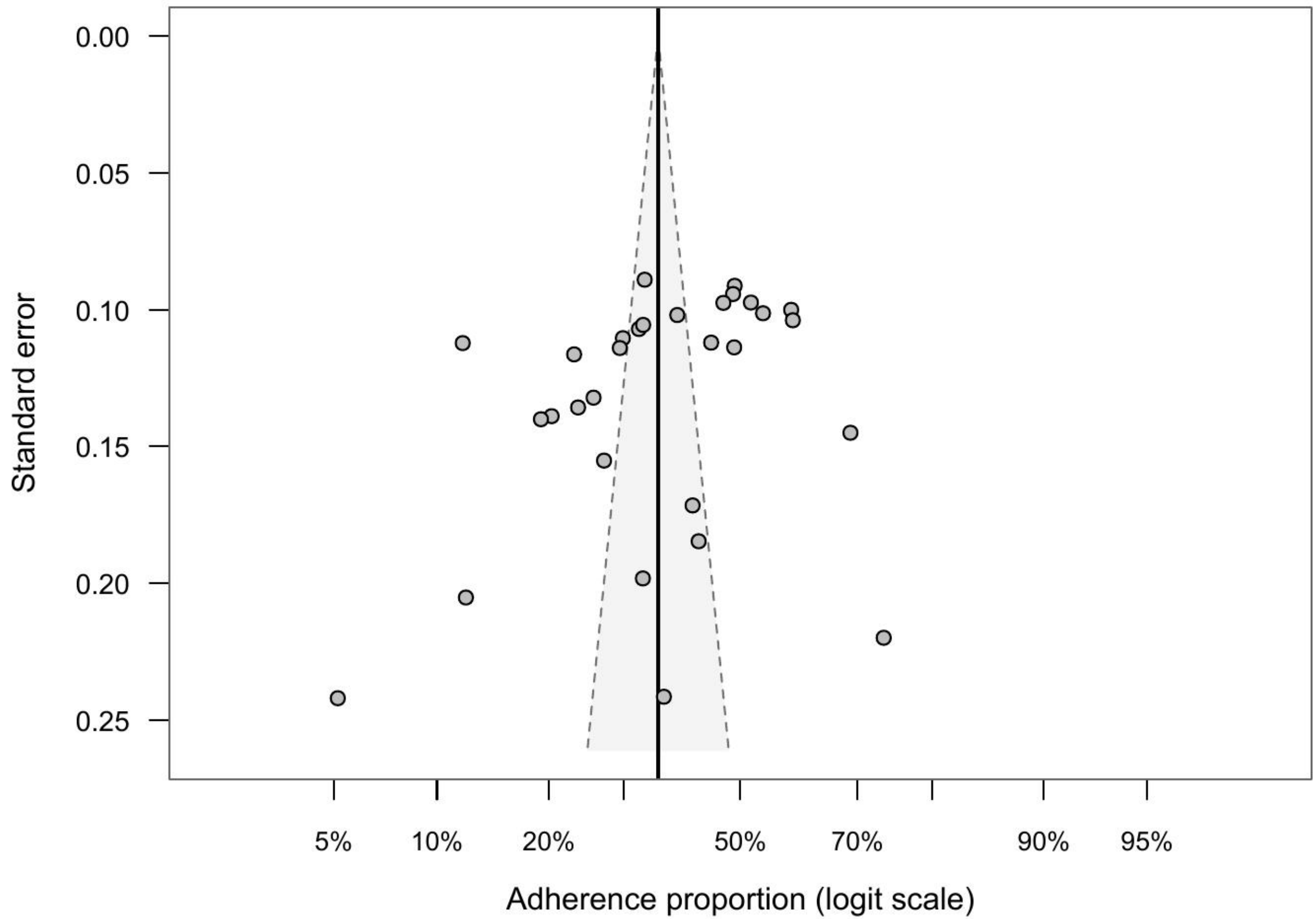

Random-effects meta-analysis | k = 30 studies | Total N = 10,121

Egger's test: t = -1.72, df = 28, p = 0.097

Appendix 16j. Overall hypertension self-care — funnel plot

Solid vertical line: pooled estimate (logit scale). Dashed lines: 95% pseudo-confidence region. Egger's regression test result is annotated above the plot.
